# Steroidal Regulation of Oviductal microRNAs Is Associated with microRNA-Processing in Beef Cows

**DOI:** 10.3390/ijms22020953

**Published:** 2021-01-19

**Authors:** Angela Maria Gonella-Diaza, Everton Lopes, Kauê Ribeiro da Silva, Ricardo Perecin Nociti, Gabriella Mamede Andrade, Jorge Eduardo Atuesta-Bustos, Juliano Coelho da Silveira, Flávio Vieira Meirelles, Mario Binelli

**Affiliations:** 1 North Florida Research and Education Center, Institute of Food and Agricultural Sciences, University of Florida, Marianna, FL 32446, USA; mario.binelli@ufl.edu; 2 Department of Animal Reproduction, School of Veterinary Medicine and Animal Science, University of São Paulo, 225, Avenida Duque de Caxias, Norte, Jardim, Elite, Pirassununga, SP 13635-900, Brazil; everton.lopes@anchieta.br (E.L.); silvakr26@gmail.com (K.R.d.S.); 3 Unianchieta, Av. Doutor Adoniro Ladeira, 94, (Km 55, 5 Rodovia Anhanguera), Jundiaí, SP 13210-795, Brazil; 4 Department of Veterinary Medicine, College of Animal Sciences and Food Engineering, University of São Paulo, Av. Duque de Caxias Norte, 225, Pirassununga, SP 13635-900, Brazil; rnociti@usp.br (R.P.N.); gabriellamamede@gmail.com (G.M.A.); julianodasilveira@usp.br (J.C.d.S.); meirellf@usp.br (F.V.M.); 5 College of Agricultural Science—Agrarian University Foundation of Colombia-UNIAGRARIA, Calle 170 No 54a-10, Bogotá 111166, Colombia; eduardoatuesta@gmail.com; 6 Department of Animal Sciences, University of Florida, PO Box 110910, Gainesville, FL 32611, USA

**Keywords:** ampulla, cattle, estradiol, isthmus, progesterone

## Abstract

Information on molecular mechanisms through which sex-steroids regulate oviductal function to support early embryo development is lacking. Here, we hypothesized that the periovulatory endocrine milieu affects the miRNA processing machinery and miRNA expression in bovine oviductal tissues. Growth of the preovulatory follicle was controlled to obtain cows that ovulated a small follicle (SF) and subsequently bore a small corpus luteum (CL; SF-SCL) or a large follicle (LF) and large CL (LF-LCL). These groups differed in the periovulatory plasmatic sex-steroid’s concentrations. Ampulla and isthmus samples were collected on day four of the estrous cycle. Abundance of DROSHA, DICER1, and AGO4 transcripts was greater in the ampulla than the isthmus. In the ampulla, transcription of these genes was greater for the SF-SCL group, while the opposite was observed in the isthmus. The expression of the 88 most abundant miRNAs and 14 miRNAs in the ampulla and 34 miRNAs in isthmus were differentially expressed between LF-LCL and SF-SCL groups. Integration of transcriptomic and miRNA data and molecular pathways enrichment showed that important pathways were inhibited in the SF-SCL group due to miRNA control. In conclusion, the endocrine milieu affects the miRNA expression in the bovine oviduct in a region-specific manner.

## 1. Introduction

The female sex-steroid hormones estradiol and progesterone play pivotal roles in the reproductive tract by programming and sustaining a proper environment for pregnancy establishment. Estradiol and progesterone modulate the abundance of molecules such as extracellular matrix proteins, growth factors, cytokines, and sex-steroids receptors. Such modulation is complex and follows spatiotemporal patterns that are not fully understood [1,2,3,4,5]. The oviduct is an organ that allows the temporary accommodation and traffic of gametes and embryos while providing specific environments for the physiological events that take place in the lumen, such as sperm capacitation, fertilization, and early embryo transport and development. Estradiol and progesterone play a crucial role in the control of these processes by the modification of the oviductal environment in a region-specific manner [6]. To advance the understanding of the role of the sex-steroids in oviductal function, we used an experimental model in which the growth of the preovulatory follicle (POF) was controlled to generate cows bearing a large POF and a subsequent large corpus luteum (LF-LCL) or a small POF and small CL (SF-SCL) [7,8]. Groups presented distinct preovulatory estradiol and post-ovulatory progesterone concentrations. Furthermore, such contrasting periovulatory endocrine milieu affected the oviductal transcriptome [9], oviductal morphology [6], and the oviductal extracellular matrix remodeling process [10], and these changes were specific to the ampulla or the isthmus regions. An intriguing finding was that although LF-LCL animals have a greater abundance of PGR and ERα in the oviduct, transcript abundance was similar for each gene in both groups. The disparity between transcript and protein abundances pointed towards regulation by post-transcriptional factors, such as microRNAs.

MicroRNAs (miRNAs) are short non-coding RNAs (19–24 nucleotides in length in their mature form) that are involved in controlling cellular function. For instance, they regulate protein translation by promoting messenger RNA (mRNA) degradation [11,12]. Based on computational predictions, it has been estimated that more than 60% of mammalian mRNAs could be targeted by at least one miRNA [13]. Similar to mRNAs, some miRNAs are differently expressed among tissues, developmental stages, and diseases.

Biogenesis of miRNAs is a complex process regulated in multiple cell compartments (see Bartel [14] for a review). Briefly, biogenesis starts with the transcription of a primary miRNA transcript (pri-miRNA) from a specific gene. This pri-miRNA is processed to a precursor miRNA (pre-miRNA, 70–100 nucleotides) by a protein complex orchestrated by DROSHA (a class 2 RNAse III-type enzyme). Pre-miRNAs are then transported from the nucleus to the cytoplasm through exportin-5 (XPO5). Next, DICER1 cleaves the pre-miRNA to the mature form of the miRNA. Mature miRNAs form an RNA-induced silencing complex (RISC) along with DICER1 and AGO proteins. RISC then binds target mRNA and regulates its translation by either cleaving or degrading the target transcript [14]. This regulatory mechanism mediated by miRNAs has brought a new layer of complexity to the study of cellular processes regulation and has been proved to be essential for the proper functioning of cells and organisms. For instance, female mice with deletion of DICER1 in somatic cells of the reproductive tract (DICER1-cKO) are sterile. These DICER1-cKO females have decreased ovulation rates, compromised oocyte and embryo integrity, and present defects in the oviducts and uterus [15]. Additionally, the miRNA biosynthesis and cellular processing machinery are also regulated by sex-steroids [11,16,17,18]. Indeed, it has been shown that sex-steroids regulate the protein expression of DICER1, DROSHA, XPO5, and Argonaute (AGO) proteins in human breast cancer [19,20] and endometrium [16].

Despite the critical role of miRNAs in the control of female reproductive function, regulation of miRNA population and miRNA processing machinery in the bovine oviduct by sex-steroids during the periovulatory period remain unknown. In the present study, we tested the hypotheses that the periovulatory endocrine milieu (1) affects the abundance of components of the miRNA processing machinery and (2) changes the miRNAs expression profile in bovine oviductal tissues.

## 2. Results

### 2.1. Animal Model

Results from the animal model used here have been reported previously [8,9,10,21]. Growth of the preovulatory follicle was controlled with the aim to obtain cows that ovulated a small follicle and subsequently bore a small corpus luteum (CL; SF-SCL group) or a large follicle and large CL (LF-LCL). The experimental model generated groups with distinct ovarian and endocrine characteristics. Specifically, follicle diameter and estradiol plasma concentration on D-1 and CL area and progesterone plasma concentration on D4 were 39%, 276%, 66%, and 75% greater in the LF-LCL compared to the SF-SCL group (*p* < 0.01), respectively.

### 2.2. RNA-Seq Analyses

A comprehensive analysis of the ampulla and isthmus transcriptome of cows in the LF-LCL and SF-SCL groups was reported previously [9,21]. In the ampulla region, a total of 692 genes showed differential expression, of which 325 and 367 were up-regulated in LF-LCL and SF-SCL samples, respectively. In the isthmus region, 590 genes showed differential expression, being 274 and 316 up-regulated in LF-LCL and SF-SCL samples, respectively. We used this dataset to perform the mRNA and miRNA integration analyses.

### 2.3. Abundance of miRNA Processing Pathway-Components

**Figure 1:**
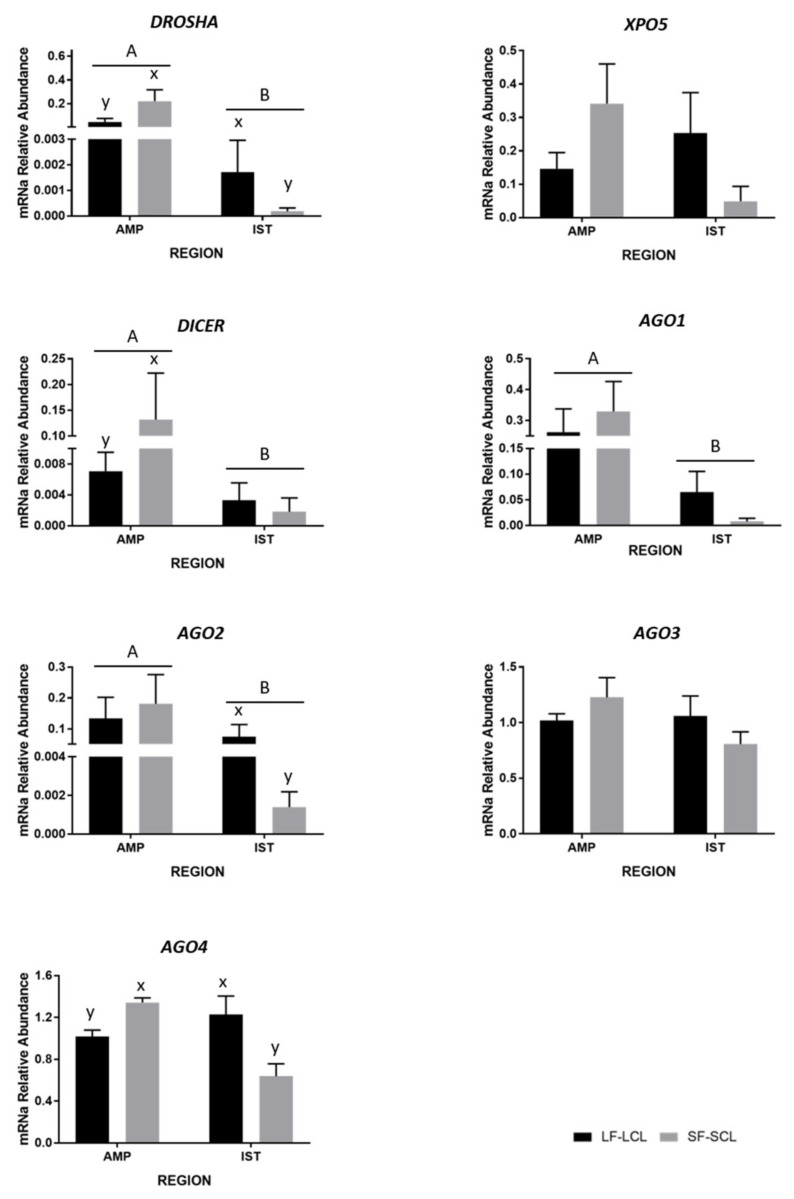
Relative levels of miRNA processing pathway-components. Mean ± SEM. Transcript abundance (*n* = 6 per group) of genes involved in the miRNA biosynthetic machinery, expression normalized to peptidylprolyl isomerase A (PPIA) and glyceraldehyde 3-phosphate dehydrogenase (GAPDH) in the ampulla (AMP) and isthmus (IST) ipsilateral to the CL from beef cows synchronized to ovulate a large (LF-LCL) or a small follicle (SF-SCL). Oviductal tissue samples were collected on day 4 of the estrous cycle. Differences between groups (x or y) and regions (A or B) are indicated when *p* value ≤ 0.05. Genes: Drosha ribonuclease III (DROSHA), Exportin 5 (XPO5), Dicer 1 ribonuclease III (DICER), RISC catalytic components: Argonaute 1, 2, 3, and 4 (AGO1, AGO2, AGO3, AGO4).

To investigate the impact of the two different endocrine environments on the abundance of the miRNAs in the oviduct, we measured the transcript abundance of the miRNA processing components using qPCR (Figure 1).

**Figure 2:**
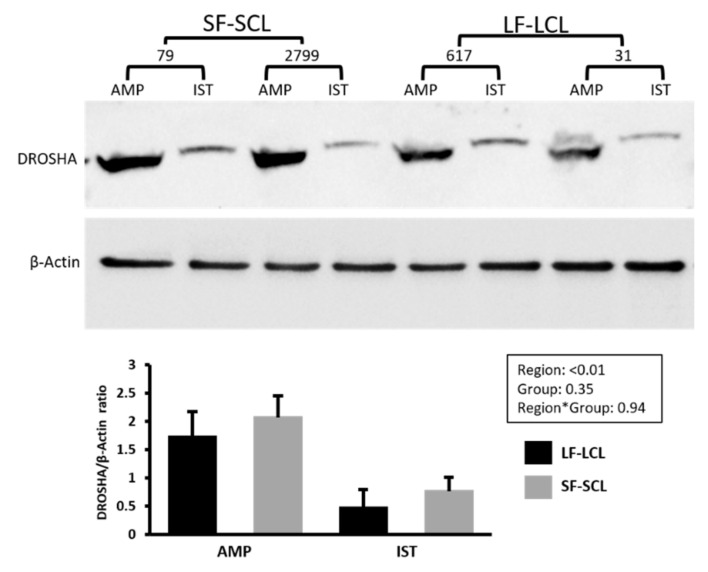
Protein levels of DROSHA from oviductal cells obtained from animals treated to ovulate a large or a small follicle. Quantification of Drosha ribonuclease III (DROSHA) protein expression (*n* = 4 per group) in the ampulla (AMP) and isthmus (IST) ipsilateral to the CL from beef cows synchronized to ovulate a large (LF-LCL) or a small follicle (SF-SCL) on day 4 of the estrous cycle. Bar graph shows the ratio of optical density of the bands corresponding to DROSHA and β-Actin.

DROSHA is the enzyme involved in processing pri-miRNA to pre-miRNA [11]. The expression level of DROSHA was ~100 times higher in the ampulla than in the isthmus. In the ampulla, SF-SCL cows presented a greater abundance of DROSHA while in the isthmus, LF-LCL cows presented a greater abundance (region x group interaction; *p* = 0.03). Then, we used Western blotting to measure DROSHA protein abundance (Figure 2). DROSHA protein was 2.99 times more abundant in the ampulla than in the isthmus. DROSHA protein was not affected by group (*p* = 0.35) or by the interaction group * region (0.94). 

XPO5 is a protein that exports pre-miRNAs from the nucleus to the cytoplasm [11], and its transcript was similarly abundant between groups and regions. DICER1 is an RNase responsible for pre-miRNAs cleavage, producing double-stranded miRNAs [22]. The abundance of DICER1 was ~16 times higher in the ampulla than in the isthmus. In addition, when comparing the groups, DICER1 was 18.7-fold greater in the ampulla of the SF-SCL group compared to the LF-LCL group, while abundance in the isthmus region was similar between groups (group*region interaction; *p* = 0.08). The AGO family plays a catalytic role in the RNA-induced silencing complex (RISC; Carthew e Sontheimer, 2009). Only AGO2 has a catalytic activity to cleave target mRNA, whereas AGO1, AGO3, and AGO4 are catalytically inactive. Their action is based on a non-cleavage gene silencing mechanism [23,24,25]. The expression of AGO1 and AGO2 were ~6 and ~4 times higher in the ampulla than in the isthmus. AGO1 transcript abundance was 8.3-fold greater in the ampulla than in the isthmus (*p* = 0.02), but similar between groups. The abundance of AGO2 transcripts was 4.2-fold greater in the ampulla than in the isthmus (*p* = 0.01), but similar between treatments. In the isthmus, abundance was greater in the LF-LCL than the SF-SCL group (70.2 fold; *p* < 0.01). AGO3 transcript abundance was not influenced by group or region. Finally, the abundance of AGO4 transcript was 2.1-fold greater in the ampulla than in the isthmus of the SF-SCL group and, conversely, 1.2-fold greater in the isthmus than ampulla of the LF-LCL group (region by group interaction; *p* = 0.03).

### 2.4. miRNA Abundance Profiles in Ampulla and Isthmus

**Figure 3:**
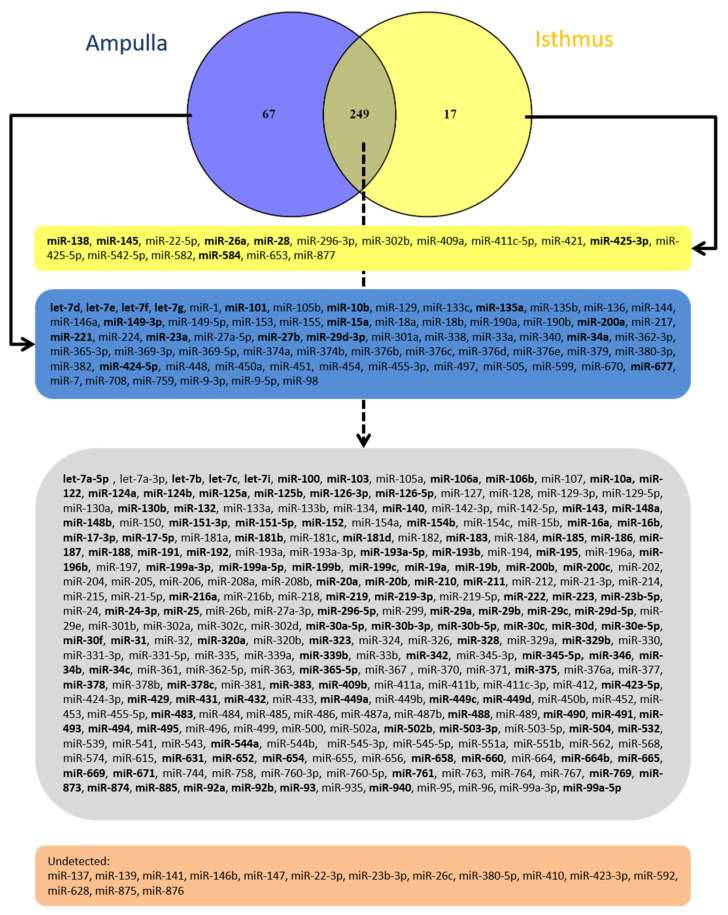
Venn diagram indicating the microRNAs detected in the ampulla and isthmus or undetected in both regions. miRNAs in bold font are those that were selected to be studied in phase 2.

Using qPCR, the abundance of 348 miRNA was evaluated in the ampulla and isthmus regions, using a pool of samples from 6 animals of each of the two groups (LF-LCL and SF-SCL). In the ampulla, 78, 146, and 92 miRNAs showed low (Cq ≥ 30), intermediate (Cq ≥ 25 to 30), and high (Cq < 25) abundance, respectively. In the isthmus, 144, 104, and 18 miRNAs showed low, intermediate, and high abundance (Supplementary Materials S1). Additionally, 32 and 82 miRNAs were not detected in the ampulla and isthmus, respectively (Figure 3). Moreover, 67 miRNAs were exclusively detected in the ampulla and 17 in the isthmus, while 249 miRNAs were detected in both regions. Eighty-eight miRNAs with the lowest Cq value (highest abundance) were chosen to be studied in individual samples. 

### 2.5. Selected miRNAs Abundance in LF-LCL and SF-SCL Groups 

**Figure 4:**
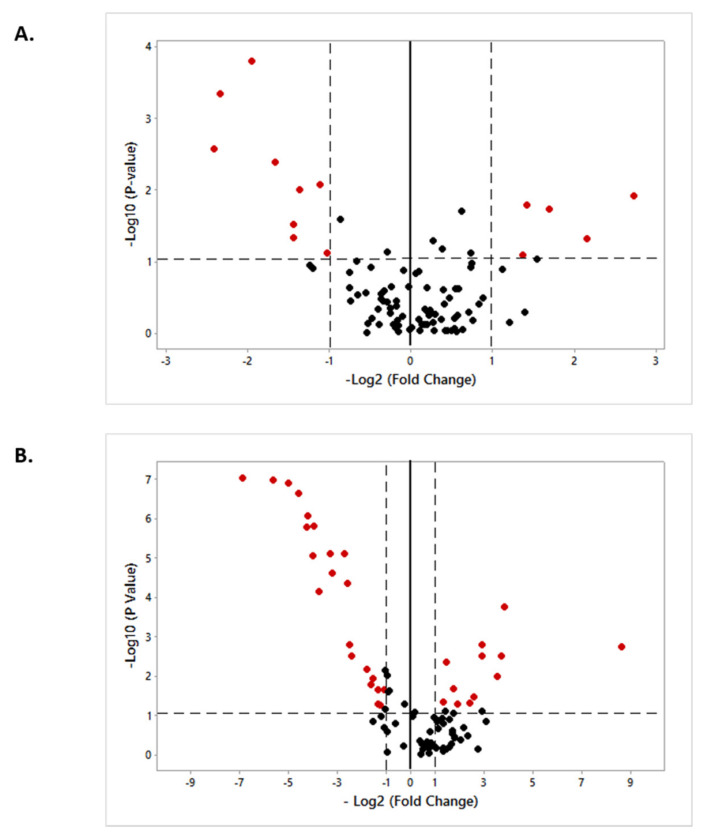
Volcano plot showing the ampulla (Panel (**A**); *n* = 6 samples per group) and isthmus (Panel (**B**); *n* = 6 samples per group) microRNA abundance of LF-LCL and SF-SCL groups, in terms of the differentially expressed microRNAs. SF-SCL/LF-LCL ratio was used to calculate-Log2 (Fold Change).

The abundance of the 88 selected miRNAs was evaluated using individual samples of the ampulla and isthmus. Differential abundance of the selected miRNAs between the LF-LCL and SF-SCL groups was evaluated in Volcano plots for the ampulla and isthmus (Figure 4). 

In the ampulla, 14 out of 88 miRNAs were differentially expressed (*p* ≤ 0.05) between groups (Table 1). In the LF-LCL animals, 5 miRNAs were up-regulated (miR-106b, miR-200b, miR-375, miR-92a, and miR-99a-5p) and 9 were down-regulated (let-7b, miR-106a, miR-181d, miR-30c, miR-339b, miR-378, miR-631, miR-92b, and miR-940).

In the isthmus, 34 out of 88 miRNAs were differentially expressed (*p* ≤ 0.05) between groups (Table 2). Of these differentially expressed miRNAs, 12 were up-regulated (miR-106a, miR-122, miR-192, miR-193b, miR-378, miR-383, miR-423-5p, miR-425-3p, miR-431, miR-654, miR-671, and miR-769) and 22 were down-regulated (miR-125b, miR-132, miR-138, miR-143, miR-154b, miR-17-3p, miR-17-5p, miR-181b, miR-188, miR-193a-5p, miR-196b, miR-199a-3p, miR-200b, miR-211, miR-219, miR-30b-5p, miR-30d, miR-345-5p, miR-409b, miR-432, miR-532, and miR-631) in the LF-LCL animals.

### 2.6. Integration of mRNAs and miRNAs Data 

**Figure 5:**
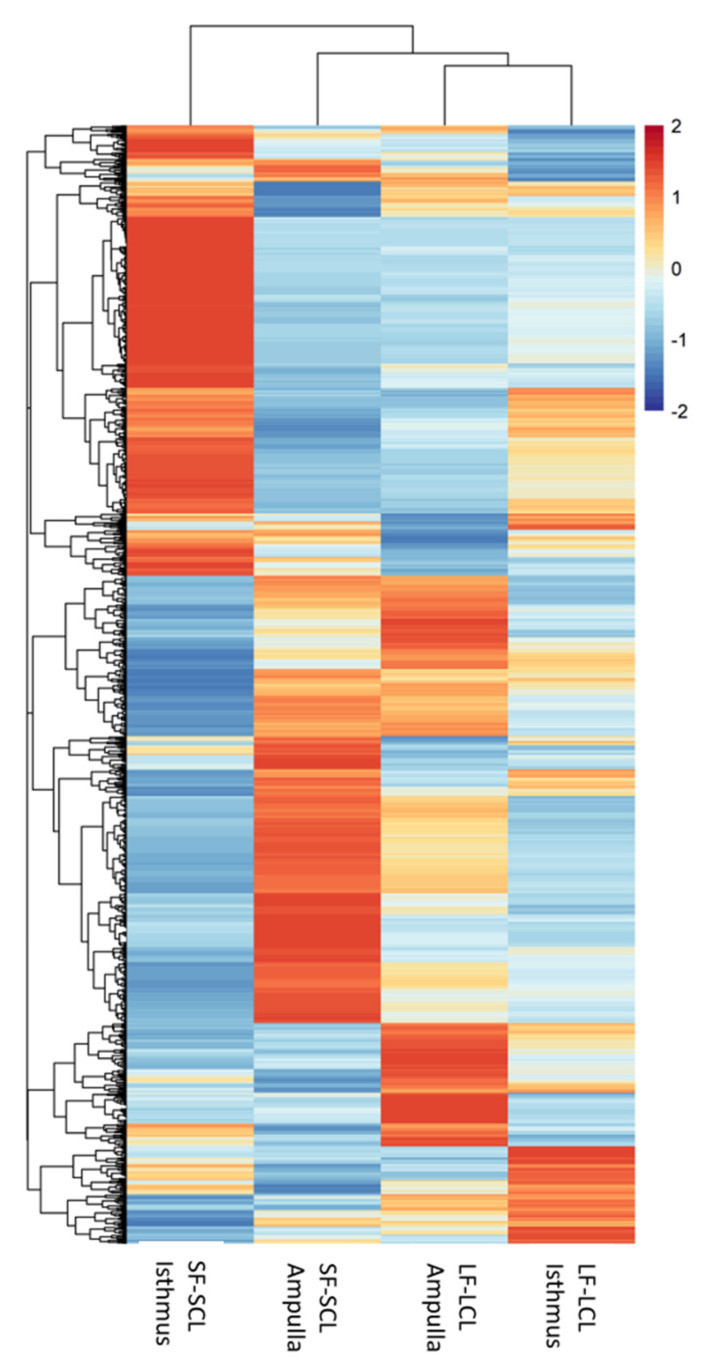
Clustering analysis of miRNA-mRNA interactome. Heat map constructed by clustering miRNA-mRNA interaction in ampulla and isthmus of the LF-LCL and SF-SCL groups (*n* = 6). Each row represents the adjusted gene expression of a mRNA predicted to be modulated by a miRNA.

We used an integration analysis of the transcriptomic data (Count numbers of the DEGs) and the miRNAs expression data (Cq values of the differentially expressed miRNAs) to obtain insights into the oviductal physiology of LF-LCL and SF-SCL groups. Using the CEMItool package, we identified 12 clusters of mRNAs (Supplementary Materials S2) and 8 of miRNAs (Supplementary Materials S3) that presented a specific expression pattern in each region and group. Next, a correlation between the expression levels of miRNA and their possible targets was conducted (Supplementary Materials S4), and 649 miRNA-mRNA correlations were founded. miR222 (correlated with 162 genes), miR191 (correlated with 92 genes), miR93b (correlated with 85 genes), miR186 (correlated with 49 genes), and let7b (correlated with 43 genes) were the miRNA associated with the downregulation of the greatest number of genes. While the transcripts that were correlated with several miRNAs were NPTXR, MAPK8IP1, ENSBTAG00000000688, MED27, and RBM4B. Figure 5 shows a heat map where every row represents the adjusted expression level of a mRNA predicted to be inhibited by a miRNA, and every column is a group/region. Interestingly, SF-SCL isthmus samples presented the most diverse clustering pattern, while both ampulla and isthmus samples of the LF-LCL group presented a more similar pattern. This means that the miRNA/mRNA correlation pattern was similar between the two oviductal regions of the LF-LCL animals, which were different from those found in both regions of the SF-SCL animals.

**Figure 6:**
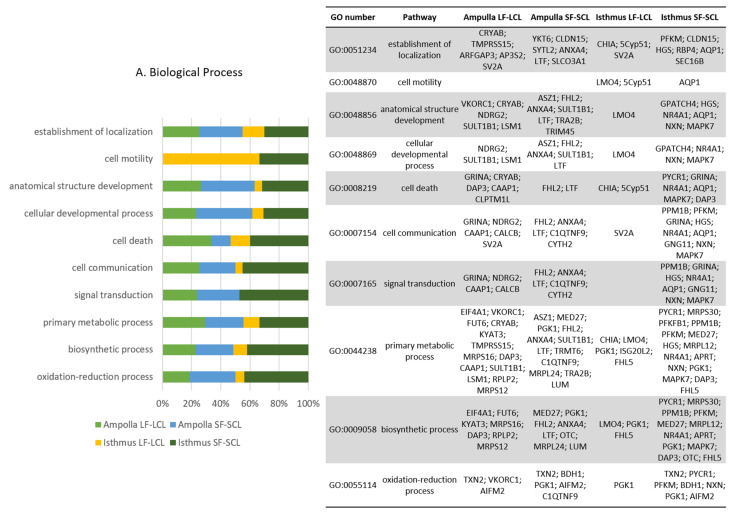

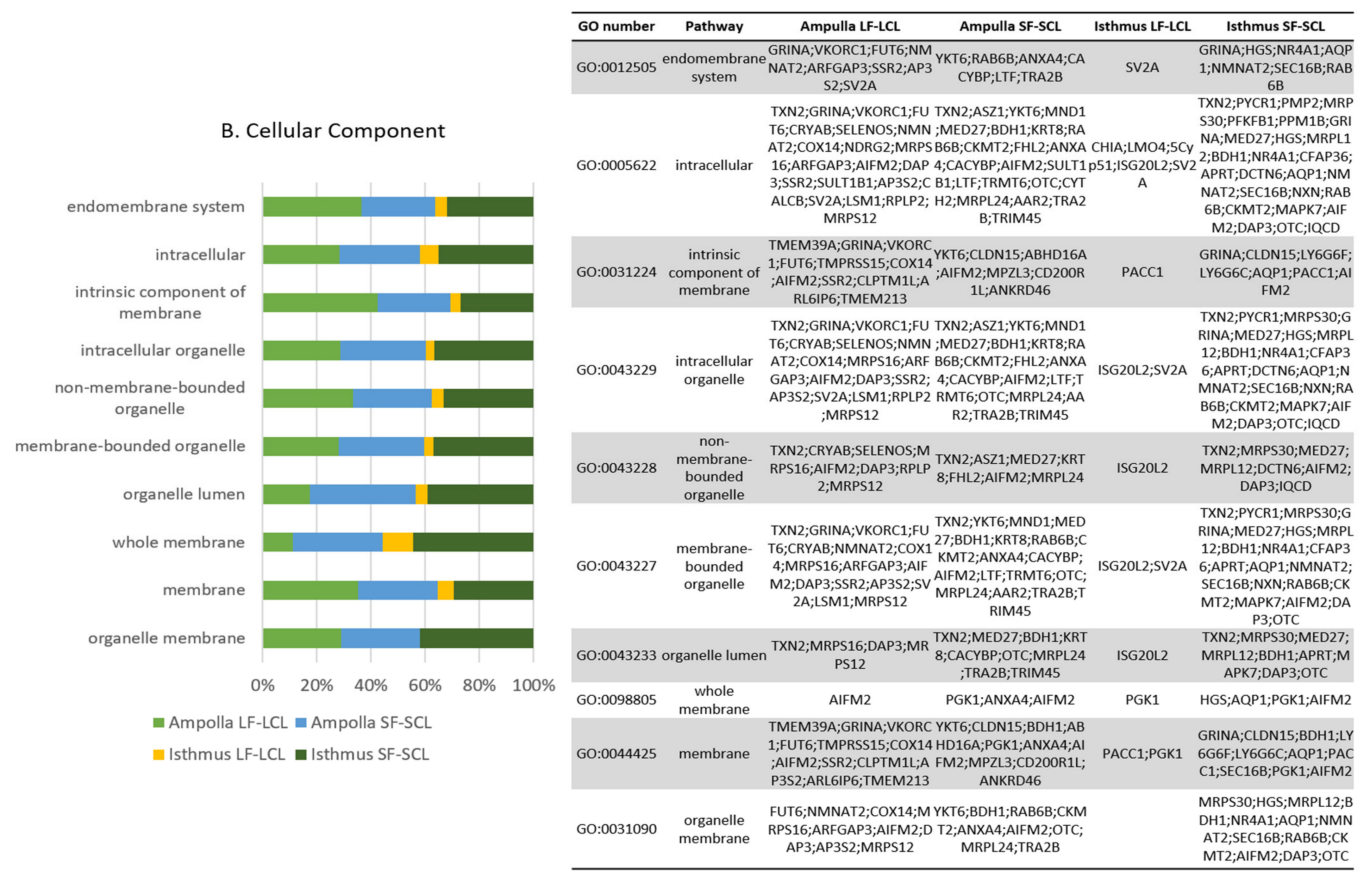

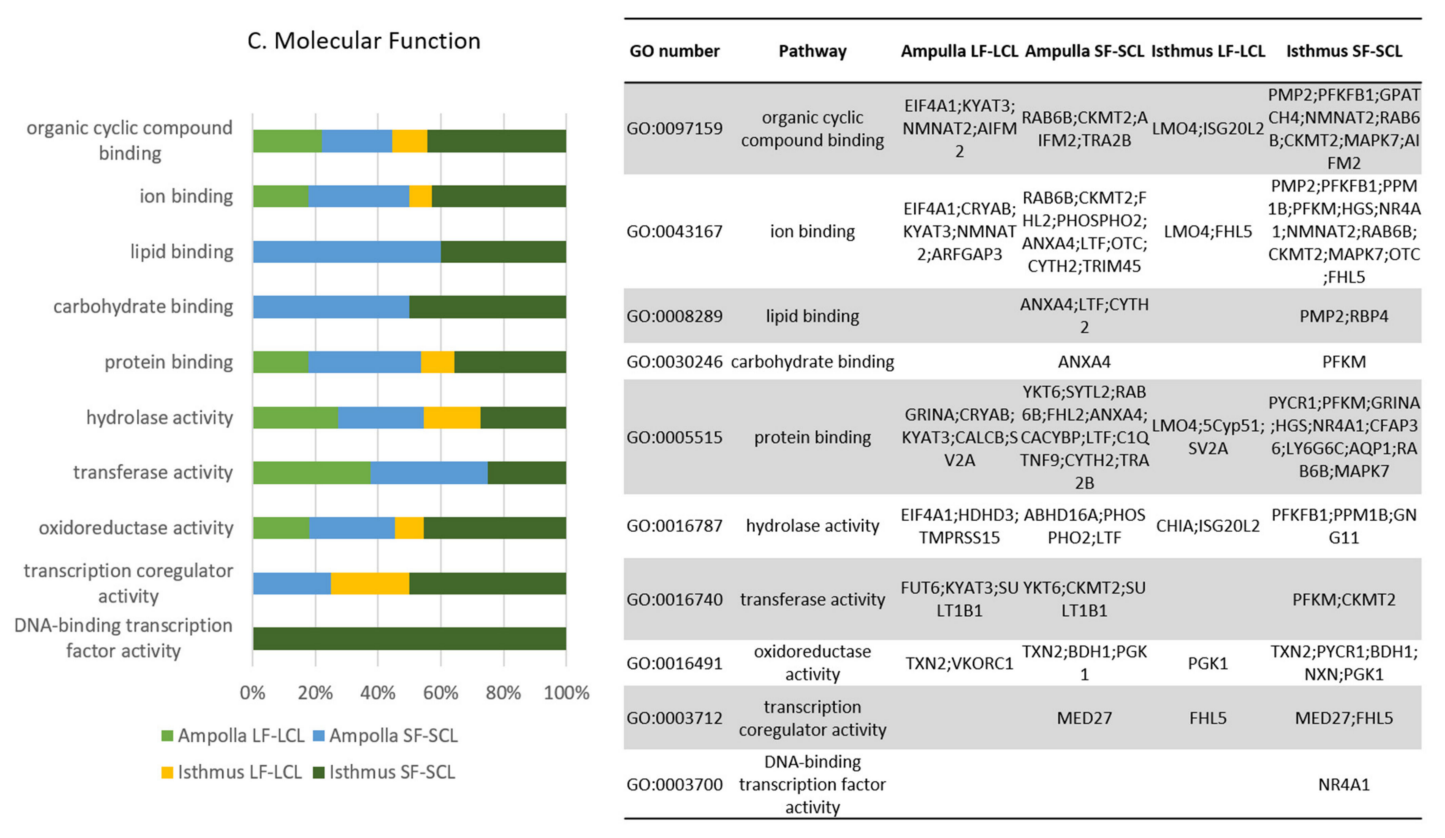
Gene Ontology (GO) analysis for the integration of miRNA/mRNA in ampulla and isthmus of the LF-LCL and SF-SCL groups. GO was run using as input all mRNA that were expressed in the transcriptomic analysis and predicted to be a target of the differentially expressed miRNAs. The GO terms were ranked according to P-adjusted and False Discovery Rate (FDR) values, and the top 10 significant GO terms for each process classification are shown. The Y axis shows the percentage of genes allocated on each pathway and on each group: (**A**) biological process, (**B**) cellular component and (**C**) molecular function.

Gene Ontology (GO) analyses were conducted using as input the genes that were predicted to be negatively regulated by the differentially expressed miRNAs (according to the miRNA-mRNA correlation analysis). The total of genes included in this analysis were 80, 32, 110, and 105 for the LF-LCL-ampulla, LF-LCL isthmus, SF-SCL-ampulla, and SF-SCL-isthmus, respectively. The GO analyses showed a series of differentially inhibited pathways in the regions and treatments, with the molecular function and biological process as the most and less affected processes classification, respectively. In the ampulla region, a total of 75 and 77 GO terms were inhibited in LF-LCL and SF-SCL animals, respectively. Moreover, in the isthmus, 59 and 81 terms were inhibited in the LF-LCL and the SF-SCL groups. Figure 6 shows the most inhibited pathways (according to their P-adjusted and FDR values; 10 pathways per processes classification are shown) and the target genes inside each of those pathways. Several pathways were commonly inhibited among the different regions/groups. However, the genes inhibited inside each pathway are not necessarily the same when comparing the different regions/groups. It is notable that the more significant proportion of inhibited genes belonged to the SF-SCL isthmus (Dark green), while the smaller proportion belonged to the LF-LCL isthmus (Yellow). 

**Figure 7:**
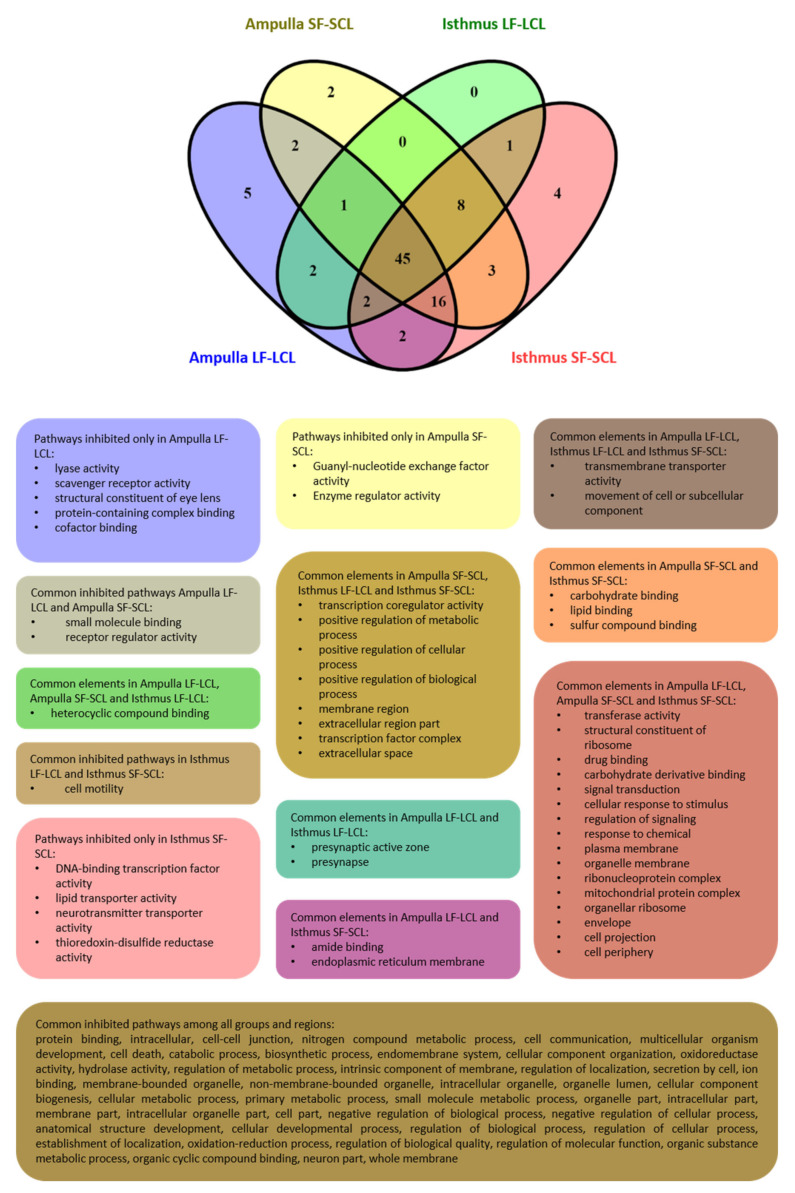
Venn diagram of the pathways modulated by miRNA-mRNA interactions. Venn diagram depicting differentially inhibited gene ontology pathways in the ampulla and isthmus of the LF-LCL and SF-SCL groups.

Because several pathways were commonly inhibited, a Venn diagram was created where the pathways modulated in each group and region were used as input (Figure 7). Presynaptic active zone and presynapse terms were commonly inhibited in both oviductal regions of the LF-LCL group. In contrast, lyase activity, scavenger receptor activity, structural constituent of eye lens, protein-containing complex binding, and cofactor binding terms were exclusively inhibited in this group’s ampulla. In the SF-SCL group, carbohydrate-binding, lipid binding, and sulfur compound binding terms were inhibited in ampulla and isthmus. Guanyl-nucleotide exchange factor activity and enzyme regulator activity were exclusively inhibited in the SF-SCL group’s ampulla, and DNA-binding transcription factor activity, lipid transporter activity, neurotransmitter transporter activity, and thioredoxin-disulfide reductase activity were exclusively inhibited in the isthmus of the same group. Some of the pathways were commonly inhibited in the ampulla (DNA-binding transcription factor activity; lipid transporter activity; neurotransmitter transporter activity; thioredoxin-disulfide reductase activity) or the isthmus (cell motility) of both groups.

## 3. Discussion

In cattle, lower concentrations of estradiol during proestrus and lower concentrations of progesterone during early diestrus dysregulate oviductal and endometrial function to result in decreased fertility [7,8,9,26]. In the oviduct, dysregulation is manifested through tissue, cellular and molecular alterations associated with altered patterns of transcription, that are regionally specific [9,10,21]. Because a key regulation of transcript abundance is via miRNAs, here we tested the hypothesis that the periovulatory sex-steroid milieu affects the miRNA processing machinery and the expression of specific miRNA levels in bovine oviductal tissues. In general, we determined that there was a greater abundance of components of the miRNA processing machinery in the ampulla than in the isthmus. Ovulation of small follicles up-regulated that machinery in the ampulla, but downregulated it in the isthmus. Consistently, such changes were associated with region-specific regulation of abundance of transcripts. There were pathways regulated by group independent of region. For example, pathways associated with enzyme regulatory activity, lipid transporter activity, and neurotransporter activity were exclusively modulated in the SF-SCL group. This is the first time that control of the miRNA-processing components mediated by sex-steroids has been shown in cattle, specifically in the female reproductive tract. The evidence presented here let us accept the hypothesis and conclude that the endocrine milieu affects the miRNAs expression in the bovine oviduct due to control of the miRNA processing machinery.

Disregarding the main effect of group and comparing the transcript abundance between ampulla and isthmus, DROSHA, DICER, AGO1, and AGO2 were as much as 100-fold more abundant in the ampulla than in the isthmus (Figure 1). These differences were consistent with the greater protein abundance of DROSHA also in the ampulla region (Figure 2). Furthermore, the miRNAs expression was different when the two oviductal regions were compared. The expression of 348 miRNAs was evaluated using a pool of samples of ampulla and isthmus. In the ampulla, 9% and 22% of miRNA were not detected or demonstrated low abundance. In contrast, in the isthmus, 23% and 41% of the miRNA were in those categories (Supplementary Materials S2). Furthermore, in the ampulla, 26% and 41% of miRNA had high or intermediate expression and in the isthmus, 5% and 30% presented these expression levels. Both, the lower abundance of miRNA and their processing pathway-components found in the isthmus could indicate that this mechanism involved with fine control of transcripts levels is more important in the ampulla than in the isthmus. This result is interesting because it could be related to the unique functions and processes that take place in each of these oviductal regions. Sperber et al. [27] studied the sensitivity of miRNA expression to DROSHA levels. They found that DROSHA levels vary among tissues and throughout cellular development, and that when DROSHA was knockdown, the expression of miRNA was reduced, specifically due to reduction of pri-miRNA. Additionally, when DICER was knockdown, the expression of miRNAs did not follow the same trend than the DROSHA knockdown. Interestingly, Feng et al. [28] showed that more conserved miRNAs are better DROSHA’s targets than latter-annotated miRNA. These agree with the present study. Here, we showed that in the ampulla region, where the abundance of DROSHA is greater, the expression of a more diverse pool of miRNAs is evident. In the isthmus, where lower amount of DROSHA is present, a less diverse population of miRNAs was found. Further studies should be conducted to establish the biological function of such intriguing region-specific mechanisms, and whether they control the specialized roles of the ampulla and isthmus. 

Sex-steroids regulate the expression of miRNA-processing pathway components in the oviduct of cattle. This is the first time that control of the miRNA-processing components mediated by sex-steroids has been shown in cattle, specifically in the female reproductive tract. Here, we reported an increased abundance of DROSHA, DICER1, and AGO4 in the ampulla of the SF-SCL group. In contrast, in the isthmus region, animals of the LF-LCL group presented an increased abundance of DROSHA, AGO2, and AGO4 (Figure 1). This indicates that the influence of the periovulatory endocrine milieu presents a region-specific pattern. There is clear evidence to support direct and indirect hormonal regulation of miRNA biogenesis. Specifically, estradiol and progesterone alter miRNA biogenesis, from transcription of components of the machinery to control of mature miRNA stability [16,29]. For example, it has been reported that ERα directly inhibits Drosha and that Exportin 5, Dicer1, Ago1, and Ago2 are low in ERα positive breast cancers [30]. Cochrane et al. [20] showed that miRNAs are differentially expressed in ERα+ and ERα− breast cancers (human cells) and that miRNAs control the expression and function of several genes such as ERα itself, DICER1, and some growth factor receptors. In addition, differential expression of DICER1, AGO3, and AGO4 was reported in the estrogen-dependent development of the chicken female reproductive tract and hen’s ovarian cancer, suggesting that the sex-steroid hormones play an essential role in the biosynthesis of miRNAs [31]. Collectively, based on research reported in other systems, it is reasonable to assume that the different periovulatory endocrine milieu mediated the differential expression of DROSHA, DICER1, and AGO4 between LF-LCL and SF-SCL groups. However, regulation is region specific and further research should be conducted to clarify mechanisms and implications for the control of oviductal function. 

Consistent with the results of the processing machinery, miRNA expression levels were also different when comparing the two groups. In the present study, 14 miRNAs in the ampulla and 34 miRNAs in isthmus were differentially expressed between LF-LCL and SF-SCL groups (Figure 4). Based on these results, a plausible interpretation is that miRNAs abundance is affected by the periovulatory endocrine milieu, due to an indirect effect of the sex-steroids in the miRNAs processing machinery. Interestingly, some of the differentially expressed miRNAs found here (miR-125b, miR-200b, miR-30d, miR-375, miR-92a) were also reported by Almiñana et al. [32] to be present in extracellular vesicles of the bovine oviductal fluid. This could suggest that some of the miRNAs that we found in the oviductal samples could be charged into extracellular vesicles and be released into the oviductal fluid to ultimately affect embryonic survival and development. Moreover, Fereshteh et al. [33] described the content of miRNAs in murine oviductosomes using next-generation sequencing in samples collected during proestrus/estrus (high circulant estradiol concentrations) or metestrus/diestrus (high circulant progesterone concentrations) stages. They described the presence of 272 miRNAs in the oviductosomes and, using an in vitro co-incubation model, they showed that oviductosomes could deliver miRNAs to sperm cells. Thus, it is possible that modulation of levels of miRNAs in extracellular vesicles and oviductosomes by sex-steroids could ultimately affect gametes, embryos, and fertility. This rationale is supported by our previous findings, which show that pregnancy per artificial insemination was greater in animals of the LF-LCL group [26]. 

The integration of miRNA and mRNA expression data shows a series of enriched pathways regulated by the differentially expressed miRNAs in ampulla and isthmus of LF-LCL or SF-SCL groups. These pathways critically control oviductal processes. For example, our predictions indicate that oxidoreduction and proliferation were dysregulated in the SF-SCL group in both ampulla and isthmus regions (Figure 6). Such functions are relevant for oviductal function, embryo survival and development, as well as fertility. The oxidoreduction process pathway was inhibited in the SF-SCL group. This process was under the control of miR-30c, miR-191, miR-186, and miR-222. Genes allocated in this pathway such as TXN2 (thioredoxin 2), BDH1 (3-hydroxybutyrate dehydrogenase 1), PGK1 (phosphoglycerate kinase 1), and AIFM2 (apoptosis-inducing factor mitochondria associated 2) were commonly inhibited in both regions, while C1QTNF9 (C1q and TNF related 9) was only inhibited in the ampulla and PYCR1 (pyrroline-5-carboxylate reductase 1), PFKM (phosphofructokinase), and NXN (nucleoredoxin) were only inhibited in the isthmus of the SF-SCL group. This result is related to our previous findings, in which the LF-LCL group presented enrichment of homeostasis-related pathways in a transcriptomic analysis [9]. In fact, NXN was up-regulated in the ampulla of the LF-LCL group. We propose that while homeostasis and oxidoreduction processes are enriched mechanisms in the LF-LCL group, they are inhibited in the SF-SCL group due to miRNA suppression. Oxidative stress is associated with impaired early development and fragmented embryos [34,35,36] and apoptosis induction of the oocyte and early embryo [37]. It is known that under the regulation of ovarian sex-steroids, oviductal cells may establish an adequate microenvironment to nourish gametes and early developing embryos by protecting from oxidative stress and potential embryotoxic effects of the oviductal mucosa innate immunity (Reviewed by Binelli et al. [38]). Moreover, when the intrauterine redox environment was analyzed in the past, cows from the LF-LCL group exhibited greater antioxidant activity than those of the SF-SCL group [39]. The SF-SCL group had lower endometrial catalase and glutathione peroxidase activity and greater lipid peroxidation and superoxide dismutase activity. Collectively, both the present data and our data on the endometrium support the notion that greater fertility associated with the LF-LCL phenotype is consistent with the inhibition of oxidoreduction mechanisms, which was less effective in the SF-SCL group, due to dysregulated miRNAs control. Future research to study this hypothesis is warranted.

Previously, we described that the SF-SCL group’s ampulla possesses less epithelial folds, less secretory cells, and less proliferating cells than LF-LCL animals [6]. In the present study, we found several genes inhibited in the ampulla and isthmus of the SF-SCL group, which could explain the lack of epithelial proliferation (Figure 6). MAPK7 was inhibited in the isthmus of the SF-SCL group by miR-191 and miR-222. This gene was allocated inside several pathways inhibited in this region/group (such as anatomical, multicellular organism development, regulation of biological process, etc.). In addition, HGS (hepatocyte growth factor-regulated tyrosine kinase substrate), ANX4 (Annexin A4), and SYTL2 (Synaptotagmin Like 2) are genes involved in exocytotic and secretory functions and were all predicted to be inhibited in the ampulla of the SF-SCL group. Finally, TRIM45 (Tripartite motif containing 45), a transcriptional repressor of the mitogen-activated protein kinase pathway, was also down-regulated. The miRNAs that were involved in this downregulation were miR-93, miR-191, miR-222, and miR-378. These collective changes relate to the limited oviductal epithelial development in cows of the SF-SCL group. We suggest that such inhibition could be mediated by miRNA regulatory mechanisms.

LTF (lactotransferrin) was predicted to be inhibited by miRNA-93 in the ampulla of the SF-SCL group. LTF is an important gene for the normal function of the female reproductive tract. LTF protein is one of the most important defense proteins present in epithelial tissues, which has a bacteriostatic activity depending on its ability to sequester iron necessary for bacterial growth and a bactericide activity through its binding to lipopolysaccharide (LPS). In our previous study [9], pathways related to immune function activation were up-regulated on the LF-LCL group’s transcriptome. Here, we found that LTF transcript abundance was inhibited in the SF-SCL group, associated with miRNA control. Further studies should be conducted to elucidate the role of miRNAs to regulate the immune function in the oviductal lumen and their relationship with embryo development.

In summary, our study evaluated the role of the periovulatory endocrine milieu in the control of the miRNA and their processing machinery. Animals of the SF-SCL and LF-LCL groups presented marked differences in the abundance of components of the miRNA processing machinery, the expression of specific miRNA, and an overall inhibition of mRNA targets. In addition, there was variation of the magnitude of miRNA regulation between oviductal regions. In general, ampulla presented a greater abundance of miRNA’s processing machinery that was related with a more diverse and abundant profile of miRNAs. The isthmus presented less abundance of components of the processing machinery and an overall smaller miRNA expression. The collective evidence let us accept the hypothesis and conclude that the endocrine milieu affects the miRNAs expression in the bovine oviduct due to direct control of the miRNA processing machinery. Although further studies are necessary to clarify miRNA’s roles in the oviductal function, it was clear that post-transcriptional control differs in animals subjected to different periovulatory sex-steroid milieus.

## 4. Materials and Methods

### 4.1. Animals, Reproductive Management, and Tissue Processing

All animal procedures were approved by the Ethics and Animal Handling Committee of the School of Veterinary Medicine and Animal Sciences of the University of São Paulo (CEUA/FMVZ-USP; protocol numbers 2281–2011 and 4293160916). The hormonal manipulations described here were validated and published in previous studies [7,8,9]. In summary, before starting the experiment, multiparous and non-lactating Nelore cows (*Bos indicus*) were selected, kept in grazing conditions (*Brachiaria brizantha* pasture), and supplemented with mineralized salt to fulfill their maintenance requirements. After gynecological examination, cows were selected according to three criteria: having no gross reproductive abnormalities, a body condition score between 3 and 4 (0, emaciated; 5, obese), and normal estrous cyclic activity (presence of a CL). A total of 56 cows were presynchronized by two intramuscular injections of prostaglandin F2 alpha analog (PGF; 0.5 mg of sodium cloprostenol; Sincrocio, Ouro Fino, Cravinhos, Brazil), 14 days apart. Ten days after the second PGF injection, cows that did not have a fresh and PGF-responsive CL (at least five days old) were removed from the experiment. The remaining cows were divided randomly to integrate one of two experimental groups, the large follicle-large CL group (LF-LCL; *n* = 20) or the small follicle, small CL group (SF-SCL; *n* = 21). All cows received a new intravaginal progesterone releasing device (1 g; Sincrogest, Ourofino) as well as 2 mg estradiol benzoate (EB; Sincrodiol, Ouro Fino Saúde Animal) injected intramuscularly (im). Only cows in the LF-LCL group received an intramuscular injection of PGF on the same day. Releasing devices were removed 8 or 8.5 days later in the LF-LCL and SF-SCL groups, respectively. All animals received two PGF injections 6 hours apart at progesterone device removal. To induce ovulation, a GnRH analog (1 μg of buserelin acetate; Sincroforte, Ouro Fino Saúde Animal) was administered (im) either 30 or 42 h after removal of the progesterone devices in SF-SCL groups and LF-LCL, respectively. The differential animal handling (PGF at progesterone device insertion and timing of device removal) during the synchronization protocol was designed to enable animals of the LF-LCL group: (1) to develop a new follicular wave under a low progesterone environment, and (2) to have more time to grow the preovulatory follicle during the proestrus. Throughout the experiment, animals were evaluated daily by transrectal ultrasonography to record follicular dynamics and luteal development. Animals were slaughtered on Day 4 (Day 0 = treatment with GnRH). Immediately after slaughter, the reproductive tract was transported on ice to the laboratory, and the oviduct ipsilateral to CL was dissected. Samples from ampulla and isthmus were frozen individually in liquid nitrogen and stored at −80 °C until RNA extraction.

### 4.2. RNA Extraction

Approximately 30 mg of each ampulla or isthmus tissue was macerated in liquid nitrogen using a stainless-steel apparatus and immediately homogenized in 200 µL of Trizol reagent (Invitrogen Life Technologies, Carlsbad, CA, USA). The homogenate was incubated for 5 min at room temperature to permit the complete dissociation of nucleoprotein complexes, and then, 128 µL of chloroform was added. The homogenate was shaken vigorously for 3 min and incubated again for 5 min at room temperature. After incubation, the homogenate was centrifuged at 12,000× *g* for 15 min at 4 °C. The aqueous phase was transferred to a fresh tube, 400 µL of isopropanol was added, and the tube was stored at −80 °C overnight. Total RNA was precipitated by centrifugation at 18,000× *g* for 8 min at 4 °C. The pellet was washed twice with 1 mL of 75% ethanol, dried under air at room temperature, and dissolved in 10 µL of diethylpyrocarbonate-treated water. Concentration and quality of total RNA were measured by a spectrophotometer (NanoDrop; Thermo Scientific, Wilmington, DE, USA).

### 4.3. mRNA Libraries, Sequencing, and Bioinformatics

Methods and general results from a transcriptomic analysis of tissues were published previously [9,21]. Briefly, ampulla and isthmus samples from three animals of each group were selected for individual RNA-Seq (please see details in the Statistical Analyses section). Only samples with a RIN ≥8 were considered suitable for RNAseq analysis (Agilent Bioanalyzer, Agilent Technologies, PA, USA). Libraries were generated according to the protocols developed by Illumina Technologies using 1 μg of total RNA as input (TruSeq, Illumina Technologies, SD, CA, USA). Briefly, polyA-selected RNA was cleaved, and fragments were used to generate the first-strand cDNA using SuperScript II reverse transcriptase and random hexamers. Next, RNaseH and DNA polymerase enzyme were used to generate the second cDNA strand, and immediately, adapter ligation and end-repair steps were performed. The resulting products were amplified using PCR, and cDNA libraries were then purified and validated using the Bioanalyzer 2100 (Agilent Technologies). The Illumina HiSeq 2000 (ampulla samples) and Illumina HiSeq 2500 (isthmus samples) platforms were used to perform the paired-end sequencing of 101-bp reads. The quality filtering was performed by seqyClean v1.3.12. (https://bitbucket.org/izhbannikov/seqyclean/get/stable.zip) using a minimum of 26 Phred quality vector and adaptor sequences from the Univec database (https://www.ncbi.nlm.nih.gov/tools/vecscreen/univec/) were used as guides to remove possible contaminants. For the next steps, only high-quality pair-ended sequences remained, and reads were mapped with Tophat v.2.0.8 [40] and Bowtie2 v2.1.0 [41] using the masked bovine genome (*Bos taurus*, UMD 3.1, NCBI). The mapping file was sorted using SAMTools v 0.1.18 [42], and read counts were obtained using the script from HTSeq-count v0.5.4p2 (http://htseq.readthedocs.io/en/release_0.9.1/). DESEq v1.12.1 [43] from R/bioconductor [44] was used to perform the differential expression analysis and, using the function estimate size factors, the normalized counts were obtained (baseMean values are the number of reads divided by the size factor or normalization constant). The standard deviation along the baseMean values was also calculated for each transcript. Only transcripts with an average of baseMean > five and mean greater than the standard deviation were analyzed to avoid artifacts caused by low expression profiles and high expression variance. The nbinom test function on DESeq was used to test for differential expression, and the threshold for evaluating significance was obtained by applying a *p* value of 0.05 FDR–Benjamini–Hochberg [45]. 

### 4.4. Reverse Transcription and qPCR of mRNA Molecules

Transcript abundance of miRNAs processing pathway-components was evaluated using qPCR (*n* = 6 animals per group; please see details in the Statistical Analyses section). After total RNA extraction, 500 ng of RNA were reverse transcribed (High Capacity cDNA Reverse Transcription Kit, Life Technologies) according to the manufacturer’s instructions. Briefly, RNA was incubated at 25 °C for 10 min, followed by incubation at 37 °C for 2 h and reverse transcriptase inactivation at 85 °C for 5 min and storage at −20 °C. The cDNA obtained was used for gene expression assays by qPCR. Step-One Plus (Life Technologies, Carlsbad, CA, USA) with SYBR Green Chemistry was used for transcript abundance analysis. Primers were designed based on GenBank Ref-Seq mRNA sequences of Drosha ribonuclease type III (DROSHA), Exportin 5 (XPO5), Dicer ribonuclease type III (DICER1), and AGO proteins (Argonaute RISC catalytic components [AGO1, AGO2, AGO3, and AGO4]). Sequences were masked to remove repetitive sequences with Repeat-Masker [46], and the masked sequences were used for primer design using the PrimerQuest software (IDT1, http://www.idtdna.com/primerquest/Home/Index). The primers’ characteristics were checked in Oligo Analyzer 3.1 software (IDT1, http://www.idtdna.com/analyzer/Applications/OligoAnalyzer/), while the specificity was compared by BLAST1 (NCBI, http://blast.ncbi.nlm.nih.gov). qPCR products from reactions containing designed primers were submitted to agarose gel electrophoresis and sequencing and identities were confirmed. Details of primers are provided in Table 3. LinRegPCR software (V2014.2; http://linregpcr.nl/) was employed to determine qPCR efficiency and Cq (quantification cycle) values per sample. Quantification was obtained after normalization of the target genes expression values (Cq values) by the geometric mean of the endogenous control expression values of cyclophilin A (PPIA) and glyceraldehyde- 3-phosphate dehydrogenase (GAPDH).

### 4.5. Protein Quantification by Western Blotting

Protein abundance of DROSHA, one of the components of the miRNAs processing pathway, was evaluated using Western blotting. About 20 mg of frozen ampulla and isthmus samples (n = 4 for each group; please see details in the Statistical Analyses section) were ground in liquid nitrogen using a mortar and pestle. Tissue homogenates were weighed and diluted (1 to 10 weight/volume) with cell lysis buffer (Cat no. ab152163, Abcam, Cambridge, UK) supplemented with 1:100 protease inhibitor (Cat. no. 80-6501-23, GE Healthcare; UK) and phenylmethylsulfonyl fluoride (PMSF, final concentration of 1 mM). Subsequently, the lysate was submitted to successive passages through hypodermic needles (21G) and then centrifuged at 10,000× g for 10 min at 4 °C. The supernatant was stored at −20 °C. After quantification of protein concentration in extracts (Pierce™ BCA Protein Assay Kit, Cat. Number: 23225; Thermo Scientific), 50 µg of protein from each extract were solubilized in loading buffer (10% SDS10, 50% glycerol, 1% bromophenol blue in 125 mM Tris HCl, and 10% b-mercaptoethanol) for 5 min at 100 °C, and subsequently separated in a 10% SDS/PAGE gel and transferred to a nitrocellulose blotting membrane (Amersham™ Protron™ 0.45 µm, Cat. no. 10600002, GE Life Sciences, Germany). Membranes were blocked in TBST (tris-buffered saline containing 0.1% of Tween-20) with 1% of bovine serum albumin (BSA Cat number A6003, Sigma_Aldrich St Louis, MO) for 2 h. Next, membranes were incubated for 90 m in a 1:1000 dilution of rabbit (polyclonal) anti-DROSHA antibody (Cat. Number IM0702, Imuny Biotechnology, Campinas, Brazil) or of monoclonal anti-b-actin peroxidase antibody produced in mouse (code A3854, Sigma Aldrich) for 1 h. Membranes were repeatedly washed in TBST and incubated with goat anti-rabbit IgG-HRP antibody (Cat. number SC-2004, Santa Cruz Biotechnology; diluted 1:10,000) for 90 min at room temperature. Bands were visualized utilizing the Clarity™ Western ECL Substrate (Cat. no. 170-5061, BioRad Laboratories Inc., USA) and the ChemiDoc MP imager (BioRad). Protein quantification was performed with Image Lab software version 4.01 (BioRad).

### 4.6. qPCR Analysis of miRNAs

The abundance of miRNAs was evaluated in two phases. First, the abundance of 348 miRNA, including three reference miRNAs (RNT43 snoRNA, Hm/Ms/Rt T1 snRNA, and miR-99b), was evaluated in the ampulla and isthmus regions, using a pool of samples from 6 animals of each of the two groups (LF-LCL and SF-SCL; please see details in the Statistical Analyses section). Stability of reference miRNAs was confirmed among groups and regions. miRNA abundance was evaluated using “Bovine Profiler Plates” as described previously in Da Silveira et al., 2017 [47]. Plates contained specific primers designed using mature miRNA sequences downloaded from the mirBase database (http://www.mirbase.org). Briefly, cDNA was synthesized from 200 ng of total RNA using the miScript Reverse Transcription Kit (Qiagen). Each reverse transcription (RT) reaction contained one μl of miScript Reverse Transcriptase Mix, 1 µL of 10× miScript Nucleics Mix, 2 µL of 5× miScript HiFlex Buffer, and RNase-free water, in order to complete 10 µL of total reaction volume. Tubes were incubated for 60 min at 37 °C, and then for 5 min at 95 °C to inactivate the enzyme and were placed on ice. Quantification of miRNAs was performed using the miScript SYBR Green PCR kit (Qiagen). PCR assays with specific primers for each miRNA and a universal reverse primer were performed in 10 μL reactions and set up with an automated pipetting station (Hamilton Microlab Starlet). Reactions without the addition of any specific primer were used as negative controls to verify no specific amplification. qPCR was conducted using the Step-One Plus (Life Technologies, Carlsbad, CA) PCR system with 96-well plates. The 10 μL PCR reaction contained 0.1 μL of RT product, 5 μL of 2× QuantiTect SYBR Green PCR Master Mix, 1 μL of 10× miScript Universal Primer, 1 μL of specific forward primer, and 2.9 μL of RNase-free water. The qPCR cycle conditions were as follows: 95 °C for 15 min, 45 cycles of 94 °C for 15 s, 55 °C for 30 s, and 70 °C for 15 s, followed by a melting curve analysis. Relative expression (ΔCq) was calculated from Cq values following normalization with the geometric mean of the reference miRNAs (RNT43 snoRNA, Hm/Ms/Rt T1 snRNA, and miR-99b), data was plotted as 2^−ΔCq^. 

In the second phase of the study, the abundance of 88 selected miRNAs was measured in individual ampulla and isthmus samples (*n* = 6 animals per group; please see details in the Statistical Analyses section) based on results from phase 1. In order to select miRNAs, the following criteria were established: (1) the Cq value (number of cycles it takes to detect a fluorescence signal above background) was ≤37 and (2) identification of a single (mature miRNA) or double (mature miRNA + pre-miRNA) peak in the melting curve. The 88 miRNAs with the lowest Cq values were chosen for the second phase. This number of miRNAs was chosen empirically, because they fit on the laboratory analytical pipeline. All the qPCR procedures were performed as described for step one. miRNA relative levels were calculated from Cq values after normalization with the geometric mean of reference miRNAs and represented using the 2^−ΔCq^ method.

### 4.7. microRNA-Targeted Transcript Modulation

Integration analysis of the transcriptomic data (Count numbers of the DEGs) and the miRNAs expression data (Cq values of the differentially expressed miRNAs) was carried out to obtain insights into the oviductal physiology. Initially, the bovine sequence of the differentially detected miRNAs and the human sequences (available in miRbase; Kozomara e Griffiths-Jones, 2014) were aligned using the pairwise sequence alignment algorithm of Clustal Omega (v. 1.2.3; http://www.ebi.ac.uk/Tools/msa/clustalo; Goujon et al., 2010; Mcwilliam et al., 2013) at EMBL-EBI. The results of these alignments can be found in (Supplementary Materials S5).

In silico predictions of miRNA-mRNA interactions are widely used; however, they do not consider the specific transcriptomic status, and the occurrence of false positives is potentially elevated. The integration of real miRNA and mRNA expression data into in silico target predictions is a more reliable method of prediction. In this study, we integrated the mRNA and miRNA using a custom R script. First, we combined miRNA data (Cq values) with RNAseq data (Count numbers) using variance stabilizing transformation. Then, a co-expression analysis was performed using CEMItool package [48] to identify clusters of genes and miRNAs specific to each region and group. 

Using the Agricolae package [49,50], a Kendall correlation was calculated to verify the relationship between miRNA and their possible targets. For graphical purposes and data consolidation, we used a z-transformation by sample, then we used the Pheatmap package [51] for data visualization with a correlation method for clusterization. Next, clusterProfiler package [52] was used to perform gene ontology (GO) and identification focusing on Biological Process, Molecular Function, and Cellular Component terms. Thus, we identified the pathways inhibited by the miRNAs’ direct action, meaning that these pathways have several genes differentially expressed in the RNAseq and that are predicted targets of the differentially expressed miRNAs. Next, we built a network analysis with igraph package [53] summarizing the relationships between miRNA, mRNA, and GO. Finally, using Venny software [54], a Venn diagram was constructed using the GO inhibited pathways list for each region (i.e., isthmus or ampulla) and group (LF-LCL or SF-SCL). The results were considered statistically significant for all analyses when the Benjamini–Hochberg test’s adjusted *p*-value was <0.05.

### 4.8. Statistical Analysis

The experiment was conducted as a completely randomized design. Animals were removed from the experiment if POF diameter on D0 was smaller than 8 mm, ovulation was detected at the D0 ultrasound examination or before (i.e., early ovulation), ovulation was detected at the D3 ultrasound examination (i.e., late ovulation), ovulation was not detected, and follicular or luteal cysts were detected at any moment during the experiment. According to these criteria, 13 animals of LF-LCL and eight animals of SF-SCL that ovulated within 24–36 h of GnRH injection were kept in the experiment. To select the samples for the different laboratory analyses, animals in each group (LF-LCL and SF-SCL) were ranked according to the following ovarian and endocrine variables: maximum diameter of the POF and estradiol concentration at D-1, CL area and progesterone concentrations at D4. Then, samples of the top-ranked animals in the LF-LCL group and the bottom-ranked animals in the SF-SCL group were selected for further laboratory analysis (qPCR for mRNA and miRNA: 6 animals per group; Western blotting: 4 animals per group; RNAseq analyses: 3 animals per group). 

All statistical analyses were performed using SAS computational software, version 9.3, for Microsoft Windows (SAS Institute Inc., Cary, NC, USA). Cow was considered the experimental unit. Data that were not normally distributed according to the Shapiro–Wilk test were transformed to natural logarithms. Concentrations of 17β- estradiol and progesterone, follicle diameter, CL area, transcript, and protein abundance of miRNAs processing pathway-components were analyzed by ANOVA using the MIXED procedure of SAS. The model included the fixed effects of group, region, and their interaction. A repeated statement was used due to repeated measures within each cow (i.e., ampulla and isthmus). In all instances, a probability of *p* ≤ 0.05 indicates a significant difference. 

## Figures and Tables

**Table 1:** Relative abundance of differentially expressed microRNAs in ampulla of LF-LCL and SF-SCL groups (*n* = 6). Fold changes were the ratio of the mean expression values of SF-SCL/LF-LCL.

MicroRNA	LF-LCL	SF-SCL	*p* Value	Log2 (Fold Change)
let-7b	0.271 ± 0.046	1.052 ± 0.634	≤0.01	1.955
miR-106a	0.008 ± 0.002	0.020 ± 0.009	0.01	1.362
miR-106b	0.047 ± 0.038	0.015 ± 0.009	0.02	−1.692
miR-181d	0.028 ± 0.010	0.088 ± 0.058	≤0.01	1.668
miR-200b	0.193 ± 0.078	0.159 ± 0.025	0.04	−0.273
miR-30c	0.026 ± 0.005	0.056 ± 0.034	≤0.01	1.114
miR-339b	0.164 ± 0.053	0.301 ± 0.261	0.02	0.870
miR-375	0.241 ± 0.085	0.036 ± 0.013	0.01	−2.732
miR-378	0.046 ± 0.015	0.125 ± 0.107	0.03	1.448
miR-631	5.740 ± 2.225	15.586 ± 8.933	0.04	1.441
miR-92a	0.142 ± 0.068	0.032 ± 0.003	0.04	−2.150
miR-92b	0.521 ± 0.250	2.785 ± 2.683	≤0.01	2.417
miR-940	0.928 ± 0.414	4.714 ± 4.399	≤0.01	2.346
miR-99a-5p	0.200 ± 0.079	0.130 ± 0.020	0.02	−0.623

**Table 2:** Relative abundance of differentially expressed microRNAs in the isthmus of LF-LCL and SF-SCL groups (*n* = 10). Fold changes were the ratio of the mean expression values of SF-SCL/LF-LCL.

MicroRNA	LF-LCL	SF-SCL	*p* Value	Log2 (Fold Change)
miR-106a	0.038 ± 0.018	0.011 ± 0.004	0.02	−1.818
miR-122	1.838 ± 1.363	0.244 ± 0.153	≤0.01	−2.910
miR-125b	0.036 ± 0.007	0.239 ± 0.202	≤0.01	2.736
miR-132	0.393 ± 0.126	9.506 ± 9.013	≤0.01	4.598
miR-138	0.406 ± 0.144	13.037 ± 11.970	≤0.01	5.005
miR-143	0.317 ± 0.174	15.701 ± 15.104	≤0.01	5.628
miR-154b	0.297 ± 0.097	5.649 ± 5.003	≤0.01	4.247
miR-17-3p	0.022 ± 0.009	0.119 ± 0.096	≤0.01	2.454
miR-17-5p	0.147 ± 0.101	0.466 ± 0.425	0.02	1.661
miR-181b	0.017 ± 0.005	0.272 ± 0.215	≤0.01	3.961
miR-188	0.186 ± 0.049	0.372 ± 0.273	0.01	0.999
miR-192	0.230 ± 0.037	0.084 ± 0.002	≤0.01	−1.457
miR-193a-5p	0.111 ± 0.049	0.289 ± 0.192	0.02	1.375
miR-193b	0.050 ± 0.022	0.004 ± 0.002	≤0.01	−3.686
miR-196b	0.032 ± 0.011	0.296 ±0.253	≤0.01	3.222
miR-199a-3p	0.007 ± 0.002	0.822 ± 0.818	≤0.01	6.885
miR-200b	0.019 ± 0.009	0.307 ± 0.256	≤0.01	4.032
miR-211	0.048 ± 0.028	0.168 ± 0.152	≤0.01	1.818
miR-219	0.060 ± 0.019	0.590 ± 0.555	≤0.01	3.295
miR-30b-5p	0.028 ± 0.012	0.374 ± 0.365	≤0.01	3.754
miR-30d	0.119 ± 0.031	0.734 ± 0.587	≤0.01	2.620
miR-345-5p	0.597 ± 0.257	11.439 ± 11.292	≤0.01	4.260
miR-378	0.184 ± 0.068	0.025 ± 0.010	≤0.01	−2.892
miR-383	0.324 ± 0.151	0.130 ± 0.027	0.04	−1.318
miR-409b	0.021 ± 0.006	0.039 ± 0.032	0.02	0.925
miR-423-5p	0.210 ± 0.052	0.024 ± 0.013	0.04	−3.149
miR-425-3p	0.639 ± 0.180	0.109 ± 0.053	0.03	−2.556
miR-431	0.086 ± 0.036	0.026 ± 0.008	0.02	−1.743
miR-432	0.153 ± 0.038	0.327 ± 0.301	0.02	1.099
miR-532	0.352 ± 0.086	0.647 ± 0.593	0.02	0.877
miR-631	105.937 ± 28.771	316.714 ± 131.871	0.01	1.580
miR-654	0.386 ± 0.220	0.034 ± 0.019	0.01	−3.509
miR-671	0.023 ± 0.022	0.000 ± 0.000	≤0.01	−8.618
miR-769	1.896 ± 1.746	0.134 ± 0.112	≤0.01	−3.824

**Table 3:** Primer sequences and amplicon characteristics of transcripts from members of the miRNA processing machinery.

GenBank ID	Gene	Primer Sequence (5′–3′) Forward/Reverse	Amplicon Length (bp)	PCR Efficiency
XM_005196186.2	Drosha ribonuclease type III (DROSHA)	AAGGCAGTGCATGTCACAGAA GCTGGGAGGTTCGTATTGGT	170	94.35
NM_203359.1	Dicer ribonuclease type III (DICER1)	TCACGATCAACACGGCCATT TTGGGGGACCAACAATGGAG	177	101.11
NM_001205899.1	Argonaute RISC catalytic component 1 (AGO1)	ATTGATGTCTCAGCCACTGCCT CTTGATCTCCTTGGTGAAGCGTAC	140	91.92
NM_205794.1	Argonaute RISC catalytic component 2 (AGO2)	TTACAAGTCGGACAGGAGCAGA AGTCGCTCTGATCATGGTTGAG	121	103.09
NM_001001133.1	Argonaute RISC catalytic component 3 (AGO3)	GGGCAGTTCAGGCAGGTATTA GTCTGTGTCCACCGTCGTT	197	109.25
XM_002686552.4	Argonaute RISC catalytic component 4 (AGO4)	CATCAGTCTGTGAGACCTGCCAT TTGACACGCTGGGAGTCTGTTAG	163	97.63
XM_002697301.3	Exportin 5 (XPO5)	AGGCTACATTGACTGGGTGC TCCAACTTGCCTTTCCTGCT	150	94.32

## Data Availability

The data that support the findings of this study are available from the corresponding author upon reasonable request.

## Supplementary Materials

Supplementary materials can be found at https://www.mdpi.com/1422-0067/22/2/953/s1.
