# Supplementary material for: Steroidal Regulation of Oviductal microRNAs Is Associated with microRNA-Processing in Beef Cows
Source: Int J Mol Sci. 2021 Jan 19;22(2):953. doi: 10.3390/ijms22020953 (PMC7835783; doi:10.3390/ijms22020953)
Supplement: Supplementary file 1 [file ijms-22-00953-s001.zip › Supplementary Material 1.docx]

**Supplemental Material 1.** MicroRNAs detected (Cq ≤ 37) in ampulla and isthmus from LF-LCL and SF-SCL cows.

+ = when the Cq value is ≥ 30; ++ = when the Cq value is ranging from ≥ 25 to 30; +++ = when the Cq value in < 25.

| microRNA | Cq Values |  | Cq Values |
| --- | --- | --- | --- |
| Detected only in Ampulla | | Detected only in Isthmus | |
| let-7d | +++ | miR-138 | ++ |
| let-7e | +++ | miR-145 | ++ |
| let-7f | +++ | miR-22-5p | + |
| let-7g | +++ | miR-26a | ++ |
| miR-1 | ++ | miR-28 | ++ |
| miR-101 | +++ | miR-296-3p | ++ |
| miR-105b | ++ | miR-302b | + |
| miR-10b | +++ | miR-409a | + |
| miR-129 | ++ | miR-411c-5p | + |
| miR-133c | + | miR-421 | + |
| miR-135a | +++ | miR-425-3p | ++ |
| miR-135b | ++ | miR-425-5p | + |
| miR-136 | ++ | miR-542-5p | ++ |
| miR-144 | + | miR-582 | + |
| miR-146a | + | miR-584 | ++ |
| miR-149-3p | +++ | miR-653 | + |
| miR-149-5p | ++ | miR-877 | ++ |
| miR-153 | + |  |  |
| miR-155 | + |  |  |
| miR-15a | +++ | Not detected in ampulla or isthmus | |
| miR-18a | ++ |  |  |
| miR-18b | + | miR-137 | |
| miR-190a | + | miR-139 | |
| miR-190b | ++ | miR-141 | |
| miR-200a | +++ | miR-146b | |
| miR-217 | + | miR-147 | |
| miR-221 | +++ | miR-22-3p | |
| miR-224 | ++ | miR-23b-3p | |
| miR-23a | +++ | miR-26c | |
| miR-27a-5p | +++ | miR-380-5p | |
| miR-27b | +++ | miR-410 | |
| miR-29d-3p | +++ | miR-423-3p | |
| miR-301a | + | miR-592 | |
| miR-338 | ++ | miR-628 | |
| miR-33a | ++ | miR-875 | |
| miR-340 | + | miR-876 | |
| miR-34a | ++ |  |  |
| miR-362-3p | ++ |  |  |
| miR-365-3p | ++ |  |  |
| miR-369-3p | + |  |  |
| miR-369-5p | + |  |  |
| miR-374a | ++ |  |  |
| miR-374b | ++ |  |  |
| miR-376b | + |  |  |
| miR-376c | ++ |  |  |
| miR-376d | + |  |  |
| miR-376e | + |  |  |
| miR-379 | ++ |  |  |
| miR-380-3p | ++ |  |  |
| miR-382 | ++ |  |  |
| miR-424-5p | +++ |  |  |
| miR-448 | + |  |  |
| miR-450a | + |  |  |
| miR-451 | ++ |  |  |
| miR-454 | + |  |  |
| miR-455-3p | + |  |  |
| miR-497 | ++ |  |  |
| miR-505 | ++ |  |  |
| miR-599 | + |  |  |
| miR-670 | + |  |  |
| miR-677 | +++ |  |  |
| miR-7 | ++ |  |  |
| miR-708 | ++ |  |  |
| miR-759 | + |  |  |
| miR-9-3p | + |  |  |
| miR-9-5p | ++ |  |  |
| miR-98 | ++ |  |  |

|  | Cq | |
| --- | --- | --- |
| microRNAs | Ampulla | Isthmus |
| Detected in both, ampulla and isthmus | | |
| let-7a-5p | +++ | + |
| let-7b | +++ | ++ |
| let-7c | +++ | + |
| let-7i | +++ | + |
| let-7a-3p | ++ | + |
| miR-100 | +++ | + |
| miR-103 | +++ | + |
| miR-105a | ++ | + |
| miR-106a | +++ | + |
| miR-106b | +++ | + |
| miR-107 | ++ | + |
| miR-10a | +++ | + |
| miR-122 | ++ | ++ |
| miR-124a | ++ | ++ |
| miR-124b | ++ | ++ |
| miR-125a | +++ | + |
| miR-125b | +++ | ++ |
| miR-126-3p | +++ | + |
| miR-126-5p | +++ | + |
| miR-127 | ++ | ++ |
| miR-128 | ++ | + |
| miR-129-3p | ++ | ++ |
| miR-129-5p | ++ | ++ |
| miR-130a | ++ | + |
| miR-130b | +++ | ++ |
| miR-132 | ++ | ++ |
| miR-133a | ++ | ++ |
| miR-133b | + | + |
| miR-134 | + | + |
| miR-140 | ++ | + |
| miR-142-3p | ++ | + |
| miR-142-5p | ++ | + |
| miR-143 | +++ | ++ |
| miR-148a | +++ | + |
| miR-148b | +++ | + |
| miR-150 | ++ | + |
| miR-151-3p | +++ | ++ |
| miR-151-5p | +++ | ++ |
| miR-152 | ++ | ++ |
| miR-154a | + | + |
| miR-154b | ++ | ++ |
| miR-154c | ++ | + |
| miR-15b | ++ | + |
| miR-16a | +++ | + |
| miR-16b | +++ | + |
| miR-17-3p | ++ | + |
| miR-17-5p | ++ | ++ |
| miR-181a | ++ | + |
| miR-181b | ++ | ++ |
| miR-181c | ++ | + |
| miR-181d | +++ | + |
| miR-182 | + | + |
| miR-183 | + | + |
| miR-184 | + | + |
| miR-185 | ++ | ++ |
| miR-186 | +++ | ++ |
| miR-187 | ++ | ++ |
| miR-188 | ++ | + |
| miR-191 | +++ | ++ |
| miR-192 | ++ | ++ |
| miR-193a | + | + |
| miR-193a-3p | ++ | + |
| miR-193a-5p | ++ | ++ |
| miR-193b | ++ | ++ |
| miR-194 | ++ | + |
| miR-195 | +++ | ++ |
| miR-196a | + | + |
| miR-196b | + | + |
| miR-197 | ++ | ++ |
| miR-199a-3p | +++ | + |
| miR-199a-5p | +++ | + |
| miR-199b | +++ | + |
| miR-199c | +++ | + |
| miR-19a | +++ | + |
| miR-19b | +++ | + |
| miR-200b | +++ | ++ |
| miR-200c | +++ | + |
| miR-202 | + | + |
| miR-204 | ++ | + |
| miR-205 | ++ | ++ |
| miR-206 | + | ++ |
| miR-208a | + | + |
| miR-208b | + | + |
| miR-20a | +++ | + |
| miR-20b | ++ | + |
| miR-21-3p | ++ | ++ |
| miR-21-5p | ++ | + |
| miR-210 | +++ | ++ |
| miR-211 | ++ | ++ |
| miR-212 | + | + |
| miR-214 | +++ | +++ |
| miR-215 | ++ | + |
| miR-216a | ++ | ++ |
| miR-216b | + | + |
| miR-218 | ++ | +++ |
| miR-219 | ++ | + |
| miR-219-3p | ++ | ++ |
| miR-219-5p | + | + |
| miR-222 | +++ | ++ |
| miR-223 | ++ | ++ |
| miR-23b-5p | ++ | + |
| miR-24 | ++ | +++ |
| miR-24-3p | +++ | ++ |
| miR-25 | +++ | + |
| miR-26b | +++ | + |
| miR-27a-3p | + | ++ |
| miR-296-5p | +++ | ++ |
| miR-299 | + | + |
| miR-29a | +++ | + |
| miR-29b | +++ | + |
| miR-29c | +++ | + |
| miR-29d-5p | +++ | ++ |
| miR-29e | ++ | + |
| miR-301b | + | + |
| miR-302a | + | + |
| miR-302c | + | + |
| miR-302d | + | + |
| miR-30a-5p | +++ | ++ |
| miR-30b-3p | ++ | + |
| miR-30b-5p | +++ | + |
| miR-30c | +++ | ++ |
| miR-30d | +++ | + |
| miR-30e-5p | +++ | + |
| miR-30f | ++ | + |
| miR-31 | +++ | ++ |
| miR-32 | + | + |
| miR-320a | +++ | +++ |
| miR-320b | ++ | ++ |
| miR-323 | +++ | +++ |
| miR-324 | ++ | + |
| miR-326 | +++ | +++ |
| miR-328 | ++ | ++ |
| miR-329a | + | + |
| miR-329b | + | ++ |
| miR-330 | ++ | ++ |
| miR-331-3p | ++ | +++ |
| miR-331-5p | ++ | + |
| miR-335 | + | + |
| miR-339a | +++ | ++ |
| miR-339b | +++ | +++ |
| miR-33b | ++ | + |
| miR-342 | ++ | + |
| miR-345-3p | ++ | ++ |
| miR-345-5p | ++ | ++ |
| miR-346 | ++ | ++ |
| miR-34b | +++ | + |
| miR-34c | +++ | + |
| miR-361 | ++ | + |
| miR-362-5p | ++ | + |
| miR-363 | + | + |
| miR-365-5p | +++ | +++ |
| miR-367 | + | + |
| miR-370 | ++ | ++ |
| miR-371 | + | + |
| miR-375 | +++ | + |
| miR-376a | + | + |
| miR-377 | + | + |
| miR-378 | ++ | ++ |
| miR-378b | ++ | + |
| miR-378c | ++ | ++ |
| miR-381 | ++ | + |
| miR-383 | ++ | ++ |
| miR-409b | + | + |
| miR-411a | ++ | + |
| miR-411b | + | ++ |
| miR-411c-3p | + | + |
| miR-412 | + | + |
| miR-423-5p | +++ | ++ |
| miR-424-3p | ++ | + |
| miR-429 | ++ | + |
| miR-431 | ++ | ++ |
| miR-432 | ++ | + |
| miR-433 | ++ | + |
| miR-449a | +++ | + |
| miR-449b | ++ | + |
| miR-449c | ++ | ++ |
| miR-449d | ++ | ++ |
| miR-450b | + | + |
| miR-452 | ++ | ++ |
| miR-453 | ++ | + |
| miR-455-5p | ++ | + |
| miR-483 | ++ | ++ |
| miR-484 | ++ | + |
| miR-485 | + | +++ |
| miR-486 | ++ | ++ |
| miR-487a | ++ | ++ |
| miR-487b | ++ | + |
| miR-488 | + | ++ |
| miR-489 | + | + |
| miR-490 | ++ | ++ |
| miR-491 | ++ | ++ |
| miR-493 | ++ | ++ |
| miR-494 | +++ | +++ |
| miR-495 | ++ | + |
| miR-496 | + | + |
| miR-499 | + | + |
| miR-500 | ++ | + |
| miR-502a | ++ | ++ |
| miR-502b | ++ | ++ |
| miR-503-3p | ++ | +++ |
| miR-503-5p | ++ | ++ |
| miR-504 | ++ | + |
| miR-532 | ++ | ++ |
| miR-539 | + | + |
| miR-541 | ++ | ++ |
| miR-543 | + | +++ |
| miR-544a | ++ | ++ |
| miR-544b | ++ | ++ |
| miR-545-3p | + | + |
| miR-545-5p | + | + |
| miR-551a | + | + |
| miR-551b | + | + |
| miR-562 | + | + |
| miR-568 | + | + |
| miR-574 | +++ | +++ |
| miR-615 | +++ | +++ |
| miR-631 | +++ | +++ |
| miR-652 | ++ | ++ |
| miR-654 | ++ | ++ |
| miR-655 | + | + |
| miR-656 | + | + |
| miR-658 | ++ | ++ |
| miR-660 | ++ | ++ |
| miR-664 | ++ | ++ |
| miR-664b | +++ | + |
| miR-665 | +++ | ++ |
| miR-669 | +++ | +++ |
| miR-671 | ++ | + |
| miR-744 | ++ | ++ |
| miR-758 | + | + |
| miR-760-3p | ++ | ++ |
| miR-760-5p | ++ | ++ |
| miR-761 | ++ | ++ |
| miR-763 | ++ | ++ |
| miR-764 | + | + |
| miR-767 | ++ | ++ |
| miR-769 | ++ | ++ |
| miR-873 | + | ++ |
| miR-874 | +++ | ++ |
| miR-885 | ++ | ++ |
| miR-92a | +++ | ++ |
| miR-92b | +++ | ++ |
| miR-93 | +++ | ++ |
| miR-935 | ++ | + |
| miR-940 | +++ | +++ |
| miR-95 | + | + |
| miR-96 | ++ | + |
| miR-99a-3p | ++ | + |
| miR-99a-5p | +++ | + |
