# Supplementary material for: Steroidal Regulation of Oviductal microRNAs Is Associated with microRNA-Processing in Beef Cows
Source: Int J Mol Sci. 2021 Jan 19;22(2):953. doi: 10.3390/ijms22020953 (PMC7835783; doi:10.3390/ijms22020953)
Supplement: Supplementary file 1 [file ijms-22-00953-s001.zip › Supplementary Material 2.docx]

**Supplementary Material 2. Clusters of genes that presented a specific expression pattern in each region (ampulla or isthmus) and group (LF-LCL or SF-SCL).** CEMITOOL package was used to identify cluster of genes with similar expression. Twelve clusters were identified and the representative figure, as well as gene IDs are presented next. Note that the scale of the Y-axis varies according to cluster.

1. **Cluster 1: 1834 genes.**


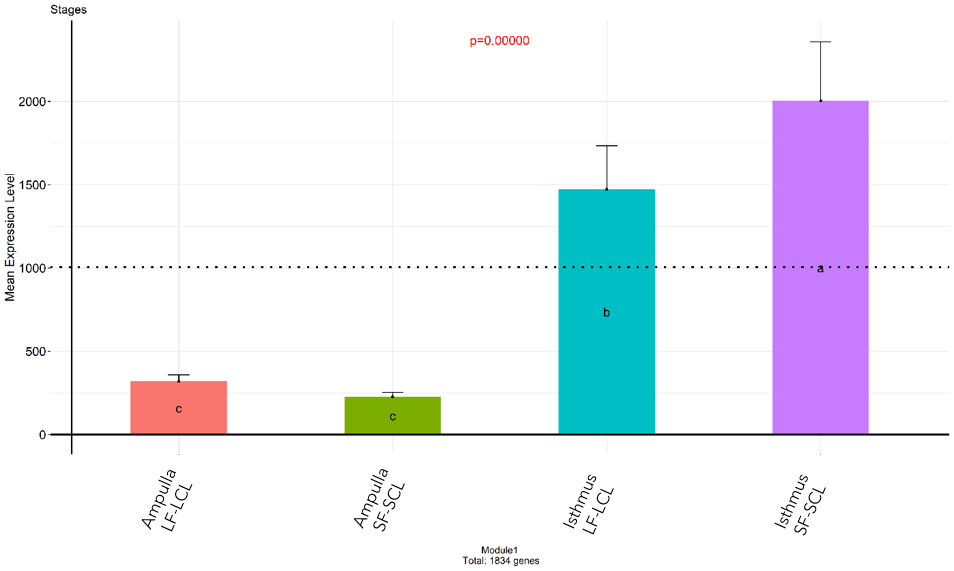


ENSBTAG00000014614_ACTA2

ENSBTAG00000014835_SPARC

ENSBTAG00000004094_SPARCL1

ENSBTAG00000011190_FLNA

ENSBTAG00000015988_MYH11

ENSBTAG00000011424_TPM2

ENSBTAG00000007196_TAGLN

ENSBTAG00000015441_ACTB

ENSBTAG00000021466_COL3A1

ENSBTAG00000012409_POSTN

ENSBTAG00000005353_DES

ENSBTAG00000013472_COL1A2

ENSBTAG00000013103_COL1A1

ENSBTAG00000011473_MYL9

ENSBTAG00000000575_TNC

ENSBTAG00000014567_MYLK

ENSBTAG00000011207_CNN1

ENSBTAG00000011802_COL6A1

ENSBTAG00000017869_CAV1

ENSBTAG00000016057_CSRP1

ENSBTAG00000015434_DSTN

ENSBTAG00000030190_COL6A3

ENSBTAG00000020056_COL12A1

ENSBTAG00000001360_RPS12

ENSBTAG00000018563_SFRP2

ENSBTAG00000017183_PDLIM3

ENSBTAG00000004796_GRP

ENSBTAG00000015473_RPS27A

ENSBTAG00000035998_CKB

ENSBTAG00000011324_EMILIN1

ENSBTAG00000006434_SYNPO2

ENSBTAG00000020116_JSP.1

ENSBTAG00000021164_SLMAP

ENSBTAG00000004915_PFN1

ENSBTAG00000013461_RPL24

ENSBTAG00000011412_LAMB1

ENSBTAG00000005077_CXCL12

ENSBTAG00000003100_SMTN

ENSBTAG00000011752_SYNM

ENSBTAG00000005146

ENSBTAG00000035959

ENSBTAG00000010793_CCDC80

ENSBTAG00000007129_MRVI1

ENSBTAG00000007343_PDLIM7

ENSBTAG00000019839_LTBP1

ENSBTAG00000019267_MMP2

ENSBTAG00000021576_LMOD1

ENSBTAG00000019517_ELN

ENSBTAG00000038495_DCLK1

ENSBTAG00000014324_ANTXR2

ENSBTAG00000003894_NDRG4

ENSBTAG00000030333

ENSBTAG00000005355_BAG2

ENSBTAG00000013347_DMPK

ENSBTAG00000048179

ENSBTAG00000047132

ENSBTAG00000003502_SPOCK1

ENSBTAG00000015185_MRGPRF

ENSBTAG00000003152_IFI27

ENSBTAG00000004555_LRP2

ENSBTAG00000003994_IGFBP3

ENSBTAG00000011327_OLFML3

ENSBTAG00000021276_TFF3

ENSBTAG00000021306_CHRDL2

ENSBTAG00000001081_PALLD

ENSBTAG00000012931_PLN

ENSBTAG00000019754_PRKCDBP

ENSBTAG00000021945_NID2

ENSBTAG00000021829_GM2A

ENSBTAG00000003398_KCNG1

ENSBTAG00000011971_NRP2

ENSBTAG00000011045_MRPS36

ENSBTAG00000048210

ENSBTAG00000014813_TCF23

ENSBTAG00000038532_FOLR1

ENSBTAG00000047998_COL5A1

ENSBTAG00000014912_FMOD

ENSBTAG00000002391_TGFB1I1

ENSBTAG00000011476_HOXA9

ENSBTAG00000006262_LIMS2

ENSBTAG00000033143

ENSBTAG00000003580_PSD

ENSBTAG00000019203_S100A4

ENSBTAG00000013300_KCNMA1

ENSBTAG00000019247

ENSBTAG00000015416_PEBP4

ENSBTAG00000045721_bta-mir-2892

ENSBTAG00000020620

ENSBTAG00000005810_PDZK1

ENSBTAG00000004170

ENSBTAG00000002092_PI16

ENSBTAG00000026809

ENSBTAG00000007068_SH3BGR

ENSBTAG00000015053_CFL2

ENSBTAG00000017733_CA2

ENSBTAG00000048268

ENSBTAG00000046337_TUBB6

ENSBTAG00000047529

ENSBTAG00000046820

ENSBTAG00000016677_C1QL3

ENSBTAG00000010517_EVPL

ENSBTAG00000001085_FAM20A

ENSBTAG00000012007_SOCS2

ENSBTAG00000001176_LRRN1

ENSBTAG00000014130_COX6C

ENSBTAG00000047064

ENSBTAG00000021211_DPT

ENSBTAG00000001209_PHLDB2

ENSBTAG00000032774_C28H10ORF116

ENSBTAG00000037600_RPS15A

ENSBTAG00000014548_FAM129A

ENSBTAG00000020532

ENSBTAG00000020564

ENSBTAG00000008921_NEXN

ENSBTAG00000003165_ADAMTS9

ENSBTAG00000019707_GATA2

ENSBTAG00000046034

ENSBTAG00000018644_PDZRN3

ENSBTAG00000015724_BPIFB1

ENSBTAG00000038783

ENSBTAG00000033727_RBPMS

ENSBTAG00000047744

ENSBTAG00000014340_KERA

ENSBTAG00000037605_BOLA-DQA1

ENSBTAG00000011578_CD44

ENSBTAG00000018777_ADCY5

ENSBTAG00000003086

ENSBTAG00000007109_ASB2

ENSBTAG00000011338_NREP

ENSBTAG00000021581_FHOD3

ENSBTAG00000040512_PCP4L1

ENSBTAG00000014239_CCNB1

ENSBTAG00000018413_FAM46B

ENSBTAG00000008121_RSPO3

ENSBTAG00000040082_HOXA10

ENSBTAG00000015551

ENSBTAG00000018098

ENSBTAG00000000198

ENSBTAG00000004924_PENK

ENSBTAG00000032022_RPL23A

ENSBTAG00000010660_CACNA1C

ENSBTAG00000008807_FBXL22

ENSBTAG00000014788_AKAP12

ENSBTAG00000031905

ENSBTAG00000022278_RPL23A

ENSBTAG00000009902_AKR1B1

ENSBTAG00000000015_FOXRED2

ENSBTAG00000035706

ENSBTAG00000034147_OLFML2B

ENSBTAG00000014596_EFHD1

ENSBTAG00000021358_BLNK

ENSBTAG00000000168

ENSBTAG00000018966_PLCE1

ENSBTAG00000011864_RGMB

ENSBTAG00000019276

ENSBTAG00000027875_CCDC141

ENSBTAG00000010850_SERTAD4

ENSBTAG00000026181_UGT1A6

ENSBTAG00000047177

ENSBTAG00000021127_GNA14

ENSBTAG00000006676_FIBIN

ENSBTAG00000003880_EMILIN2

ENSBTAG00000021119_TDGF1

ENSBTAG00000046056

ENSBTAG00000046850

ENSBTAG00000007740_BMK

ENSBTAG00000001728_IGSF10

ENSBTAG00000008135_SLIRP

ENSBTAG00000002758_THBD

ENSBTAG00000008160_MBOAT2

ENSBTAG00000008544_SALL1

ENSBTAG00000047586_NPY1R

ENSBTAG00000007300_FHL3

ENSBTAG00000008452_IHH

ENSBTAG00000048157

ENSBTAG00000003867

ENSBTAG00000047613_HTRA3

ENSBTAG00000009238_HOXC6

ENSBTAG00000044126_SNTB1

ENSBTAG00000034973

ENSBTAG00000011037_RBPMS2

ENSBTAG00000016819_FABP3

ENSBTAG00000021938_ARHGAP44

ENSBTAG00000009835_CACNA1G

ENSBTAG00000015690_PLIN4

ENSBTAG00000017496_ATP5I

ENSBTAG00000000021

ENSBTAG00000011082_IGF-I

ENSBTAG00000006432_KCNE4

ENSBTAG00000010148

ENSBTAG00000010875_MSX1

ENSBTAG00000012307_DTNA

ENSBTAG00000044073_CD248

ENSBTAG00000015366_SFRP4

ENSBTAG00000048225

ENSBTAG00000019587_PI15

ENSBTAG00000015307_FBN2

ENSBTAG00000024731

ENSBTAG00000011941_LYZ1

ENSBTAG00000012328_GHRL

ENSBTAG00000047700

ENSBTAG00000011704

ENSBTAG00000045588

ENSBTAG00000044027_ITGBL1

ENSBTAG00000045734_LRRC26

ENSBTAG00000011139_BCHE

ENSBTAG00000008793_RNASE1

ENSBTAG00000000802_LYVE1

ENSBTAG00000047569

ENSBTAG00000004645_ADAMTSL3

ENSBTAG00000006600_ARPC2

ENSBTAG00000020028_RBP1

ENSBTAG00000014665_ADAMTS2

ENSBTAG00000015549_PCDH18

ENSBTAG00000011882_SLC24A3

ENSBTAG00000047787

ENSBTAG00000011153_MGAT4C

ENSBTAG00000011328_CGREF1

ENSBTAG00000001032_PYGM

ENSBTAG00000039820_CLDN8

ENSBTAG00000040490_CCDC3

ENSBTAG00000019798_PIGR

ENSBTAG00000018941_CPXM2

ENSBTAG00000008020

ENSBTAG00000009260_GPM6B

ENSBTAG00000005096_COX7A2

ENSBTAG00000017071_C1QTNF3

ENSBTAG00000023002_JPH2

ENSBTAG00000008275_GREB1L

ENSBTAG00000002188_C16orf89

ENSBTAG00000002551_KCNJ8

ENSBTAG00000021751_RASEF

ENSBTAG00000046120

ENSBTAG00000030885_SSC5D

ENSBTAG00000019585_MYOM1

ENSBTAG00000003794_GNAO1

ENSBTAG00000004680_SLC13A5

ENSBTAG00000015107_SLC16A1

ENSBTAG00000005717_GJB5

ENSBTAG00000020590_FZD2

ENSBTAG00000002821_CILP

ENSBTAG00000033083

ENSBTAG00000000061_PCDH7

ENSBTAG00000007444_C1H21ORF7

ENSBTAG00000047202_GRIN1

ENSBTAG00000046161

ENSBTAG00000030705_DACT3

ENSBTAG00000010129_SLITRK5

ENSBTAG00000020238_RIMS1

ENSBTAG00000020665_GFRA2

ENSBTAG00000018843_SERPINA1

ENSBTAG00000037775

ENSBTAG00000017375_C6H4orf32

ENSBTAG00000021347_CWH43

ENSBTAG00000012461_PRSS16

ENSBTAG00000008956_CD8B

ENSBTAG00000047240

ENSBTAG00000007678_MKX

ENSBTAG00000045727

ENSBTAG00000005745_HPSE

ENSBTAG00000000042_PYCR1

ENSBTAG00000010940_HSPB7

ENSBTAG00000047986

ENSBTAG00000006735_STAC

ENSBTAG00000010931_SSBP1

ENSBTAG00000017020_S100G

ENSBTAG00000020127_TKTL1

ENSBTAG00000009656_BOLA-DQA2

ENSBTAG00000017430_PNCK

ENSBTAG00000011299

ENSBTAG00000001029_RASGRP2

ENSBTAG00000005685_HSD11B2

ENSBTAG00000030599_SMOC1

ENSBTAG00000001966_PDGFRL

ENSBTAG00000046261

ENSBTAG00000047677

ENSBTAG00000038128_BOLA-DQA5

ENSBTAG00000020342_MYOC

ENSBTAG00000004824_REEP1

ENSBTAG00000014655_MYO1A

ENSBTAG00000016748

ENSBTAG00000005719_GJB4

ENSBTAG00000019977

ENSBTAG00000014960_SCHIP1

ENSBTAG00000003130_CHRNA3

ENSBTAG00000006562

ENSBTAG00000009014_UPK1B

ENSBTAG00000006631_GLI1

ENSBTAG00000047965

ENSBTAG00000019859_B3GALT2

ENSBTAG00000010060_SLC43A3

ENSBTAG00000009876_C4BPA

ENSBTAG00000006589_CFTR

ENSBTAG00000010161_CCL21

ENSBTAG00000000604_GPNMB

ENSBTAG00000009195

ENSBTAG00000013662_COL8A1

ENSBTAG00000010578_IL17B

ENSBTAG00000003258

ENSBTAG00000021007_FOLR1

ENSBTAG00000003308_GRID2IP

ENSBTAG00000004303_SLC27A2

ENSBTAG00000019291_GRB14

ENSBTAG00000001580_CLGN

ENSBTAG00000046256_TMEM132C

ENSBTAG00000009696_ACTN2

ENSBTAG00000018460_ADAMTS16

ENSBTAG00000021069_PBK

ENSBTAG00000012503_MGAT3

ENSBTAG00000001740_HAPLN3

ENSBTAG00000048169

ENSBTAG00000018125_KIF5C

ENSBTAG00000002319_HMCN2

ENSBTAG00000046451

ENSBTAG00000027923

ENSBTAG00000034433

ENSBTAG00000016982_MCOLN3

ENSBTAG00000020381_FCF1

ENSBTAG00000006894_INOS

ENSBTAG00000018583

ENSBTAG00000011634_ACPP

ENSBTAG00000026249

ENSBTAG00000030735_COX7B

ENSBTAG00000031247_MUC12

ENSBTAG00000007665_NPR3

ENSBTAG00000024910_GALR2

ENSBTAG00000003352

ENSBTAG00000018214_SHISA2

ENSBTAG00000047174

ENSBTAG00000013081_PSPH

ENSBTAG00000004411_CRISPLD1

ENSBTAG00000000818_PLEKHG7

ENSBTAG00000025803_C15orf59

ENSBTAG00000005606_HOXC9

ENSBTAG00000034323_SLC2A5

ENSBTAG00000002180

ENSBTAG00000040418_PPP1CA

ENSBTAG00000021077_BOLA-DQB

ENSBTAG00000039380

ENSBTAG00000025604

ENSBTAG00000047703

ENSBTAG00000004860_SLC27A6

ENSBTAG00000022306

ENSBTAG00000013250_PCSK1N

ENSBTAG00000012884_MPTX

ENSBTAG00000006326_ALDH1L2

ENSBTAG00000038913

ENSBTAG00000021252_TMEM35

ENSBTAG00000015902_FP

ENSBTAG00000045619_RPL39

ENSBTAG00000000062_RASL12

ENSBTAG00000035855

ENSBTAG00000011833_GRIA3

ENSBTAG00000012540

ENSBTAG00000004612_SORCS3

ENSBTAG00000017069_FAM198B

ENSBTAG00000012889_NETO1

ENSBTAG00000007635_PLCL1

ENSBTAG00000032130_CATSPER2

ENSBTAG00000015532_NR0B1

ENSBTAG00000013155_COL2A1

ENSBTAG00000020223_CASQ1

ENSBTAG00000003856

ENSBTAG00000046813

ENSBTAG00000046350_PKP1

ENSBTAG00000012467_MASP1

ENSBTAG00000007680_THSD7A

ENSBTAG00000021481_CA14

ENSBTAG00000001476_BT.105339

ENSBTAG00000002290

ENSBTAG00000007704_ELOVL7

ENSBTAG00000004594_MMP7

ENSBTAG00000047437

ENSBTAG00000037452

ENSBTAG00000046476_IGSF1

ENSBTAG00000020650_ADCYAP1

ENSBTAG00000015395

ENSBTAG00000003837_RSPO1

ENSBTAG00000037931_RPL39

ENSBTAG00000003137_LPPR1

ENSBTAG00000004129_CCL11

ENSBTAG00000003238_MEOX2

ENSBTAG00000022886_RYR2

ENSBTAG00000024639

ENSBTAG00000039524

ENSBTAG00000018445_GRIN3A

ENSBTAG00000008359

ENSBTAG00000019143

ENSBTAG00000034844_RPL39

ENSBTAG00000010103_TRIM9

ENSBTAG00000019332_SLC9C1

ENSBTAG00000007325_TPSB1

ENSBTAG00000021446

ENSBTAG00000014781

ENSBTAG00000045759

ENSBTAG00000044119_KSR2

ENSBTAG00000017602_TMEM45B

ENSBTAG00000010010

ENSBTAG00000006877_MMP-16

ENSBTAG00000001357_PRMT1

ENSBTAG00000027516_APLNR

ENSBTAG00000012584_GJB3

ENSBTAG00000018554

ENSBTAG00000045993

ENSBTAG00000047048

ENSBTAG00000023177

ENSBTAG00000019268

ENSBTAG00000003319_FLRT3

ENSBTAG00000046633

ENSBTAG00000018833_SVOP

ENSBTAG00000022137

ENSBTAG00000006738_GPR68

ENSBTAG00000037786_NALCN

ENSBTAG00000016741_TOX2

ENSBTAG00000017483

ENSBTAG00000019453_PTGES

ENSBTAG00000013462_L1CAM

ENSBTAG00000009870_HES6

ENSBTAG00000008074_C1QTNF6

ENSBTAG00000010719_ANGPTL1

ENSBTAG00000024560_MGC151592

ENSBTAG00000021924_PLA2G4E

ENSBTAG00000038421_NPY5R

ENSBTAG00000016627_WASF1

ENSBTAG00000010472_TNFAIP8L3

ENSBTAG00000007051_CLDN23

ENSBTAG00000012460_ELF5

ENSBTAG00000013820

ENSBTAG00000045498_SULT2B1

ENSBTAG00000017722_F5

ENSBTAG00000026437_ULBP3

ENSBTAG00000045505_PSIP1

ENSBTAG00000023941_CPN1

ENSBTAG00000031538

ENSBTAG00000039425

ENSBTAG00000047265

ENSBTAG00000013984_KL

ENSBTAG00000047601_MGC140151

ENSBTAG00000032944

ENSBTAG00000018010_ABCA4

ENSBTAG00000046879

ENSBTAG00000021916_MYL4

ENSBTAG00000017132_TTC22

ENSBTAG00000011370

ENSBTAG00000002330

ENSBTAG00000007235_NTNG2

ENSBTAG00000002936_PRRX2

ENSBTAG00000019249_HS3ST6

ENSBTAG00000009692

ENSBTAG00000017717_RALYL

ENSBTAG00000046818

ENSBTAG00000008477

ENSBTAG00000047936

ENSBTAG00000004010_PAPPA

ENSBTAG00000016644_AQPEP

ENSBTAG00000035171_SERPINB12

ENSBTAG00000046838

ENSBTAG00000047295_MGC140151

ENSBTAG00000010366_HCRTR1

ENSBTAG00000040070_SCGB1D

ENSBTAG00000031362_NXPE2

ENSBTAG00000010866_BMPER

ENSBTAG00000048014

ENSBTAG00000045765

ENSBTAG00000005972

ENSBTAG00000000059_RPL22

ENSBTAG00000009093_GAL3ST2

ENSBTAG00000021969_KLHL29

ENSBTAG00000047710

ENSBTAG00000019221

ENSBTAG00000012969_DKK2

ENSBTAG00000016968_CHST4

ENSBTAG00000035182_SERPINB13

ENSBTAG00000004743

ENSBTAG00000001325_UPB1

ENSBTAG00000015204_SMPX

ENSBTAG00000046495

ENSBTAG00000008539_ENHO

ENSBTAG00000017981_ADRB3

ENSBTAG00000003349_JAKMIP2

ENSBTAG00000008150_PKIA

ENSBTAG00000008352_ART4

ENSBTAG00000026732_PLA2G1B

ENSBTAG00000014011_TMOD2

ENSBTAG00000047294_CUL1

ENSBTAG00000010836_PRRT3

ENSBTAG00000007302

ENSBTAG00000000257_MRAP2

ENSBTAG00000047125

ENSBTAG00000043993_C21orf62

ENSBTAG00000031704_MGC133804

ENSBTAG00000037881

ENSBTAG00000020667_KCNQ3

ENSBTAG00000046322

ENSBTAG00000005288_GFRA3

ENSBTAG00000019297_GFRA1

ENSBTAG00000020368

ENSBTAG00000009406

ENSBTAG00000012834_ARSI

ENSBTAG00000006273_SULT4A1

ENSBTAG00000047884

ENSBTAG00000009268

ENSBTAG00000026913_HHIPL1

ENSBTAG00000016306_PFDN4

ENSBTAG00000040106_CHADL

ENSBTAG00000047580

ENSBTAG00000002772_GAL3ST3

ENSBTAG00000046365

ENSBTAG00000047417_CYLC1

ENSBTAG00000046915

ENSBTAG00000000300_KIAA2022

ENSBTAG00000003919_KIAA0408

ENSBTAG00000027434_SORCS2

ENSBTAG00000002354

ENSBTAG00000013147_NEFH

ENSBTAG00000003676

ENSBTAG00000038272_SOX1

ENSBTAG00000045678

ENSBTAG00000010634_C6H4ORF31

ENSBTAG00000021033_SGK2

ENSBTAG00000045747_5_8S_rRNA

ENSBTAG00000009355_SNAP91

ENSBTAG00000008894

ENSBTAG00000017524_CBLN4

ENSBTAG00000021992_MURC

ENSBTAG00000013625

ENSBTAG00000033224

ENSBTAG00000046245

ENSBTAG00000046595

ENSBTAG00000005019_PRSS50

ENSBTAG00000027134_DYNC1I1

ENSBTAG00000017098_BPIFA1

ENSBTAG00000046649_SGPP2

ENSBTAG00000030493_RERGL

ENSBTAG00000034681_CA10

ENSBTAG00000039346

ENSBTAG00000046003_HIST2H2AC

ENSBTAG00000024545_DCHS2

ENSBTAG00000045825_5_8S_rRNA

ENSBTAG00000046193

ENSBTAG00000002266_NPL

ENSBTAG00000018224_GLP1R

ENSBTAG00000012621_RTN4RL2

ENSBTAG00000032156

ENSBTAG00000016752_ASB10

ENSBTAG00000046468

ENSBTAG00000018989_GRM3

ENSBTAG00000048255_5_8S_rRNA

ENSBTAG00000007997

ENSBTAG00000021009_TRIM55

ENSBTAG00000009418_FAM131B

ENSBTAG00000047401_5_8S_rRNA

ENSBTAG00000002027_FAM167B

ENSBTAG00000037429_HTR1B

ENSBTAG00000048162_C5H12ORF4

ENSBTAG00000020809_SLC36A2

ENSBTAG00000021283_KCNF1

ENSBTAG00000047231_TNNI1

ENSBTAG00000048148_5_8S_rRNA

ENSBTAG00000011098_TTLL8

ENSBTAG00000007493

ENSBTAG00000046882_5_8S_rRNA

ENSBTAG00000045952_5_8S_rRNA

ENSBTAG00000045750_DDX3Y

ENSBTAG00000012837_COL6A6

ENSBTAG00000030490

ENSBTAG00000012866_THBS4

ENSBTAG00000008113_OSR1

ENSBTAG00000018753_TMEM163

ENSBTAG00000031532_DACT2

ENSBTAG00000047907

ENSBTAG00000048030

ENSBTAG00000015027

ENSBTAG00000017411_AK5

ENSBTAG00000027832_CNTNAP2

ENSBTAG00000044427_RNaseP_nuc

ENSBTAG00000046519_C5H12ORF4

ENSBTAG00000033835_MPZ

ENSBTAG00000015775_CHRNA7

ENSBTAG00000005562_GALNT13

ENSBTAG00000000189_LHFPL3

ENSBTAG00000036061

ENSBTAG00000017821

ENSBTAG00000004232_TMIE

ENSBTAG00000033326_DPEP1

ENSBTAG00000011759_C1QL1

ENSBTAG00000033803_FABP7

ENSBTAG00000013059

ENSBTAG00000048274

ENSBTAG00000039321

ENSBTAG00000004591

ENSBTAG00000032806

ENSBTAG00000011962_PLA2G3

ENSBTAG00000010245_SPRY3

ENSBTAG00000010306_RXFP1

ENSBTAG00000016928_PDZD7

ENSBTAG00000000376_P2RX3

ENSBTAG00000026822

ENSBTAG00000007486_CEL

ENSBTAG00000006176_GRIA4

ENSBTAG00000047602

ENSBTAG00000033109

ENSBTAG00000021132_SYNPO2L

ENSBTAG00000032456_HIST2H2AC

ENSBTAG00000001899_KCNH7

ENSBTAG00000005236_CCKBR

ENSBTAG00000012182_DIRAS3

ENSBTAG00000037899_DLK

ENSBTAG00000047084

ENSBTAG00000010543_FGFR4

ENSBTAG00000006899_JPH3

ENSBTAG00000000576_GPR64

ENSBTAG00000018193_KCNK3

ENSBTAG00000047194

ENSBTAG00000008138_HOXA2

ENSBTAG00000037604_EFNB3

ENSBTAG00000008357

ENSBTAG00000027126_MUC15

ENSBTAG00000020992_RHBDL3

ENSBTAG00000038496_CR2

ENSBTAG00000015143

ENSBTAG00000025410_EGFL8

ENSBTAG00000048114

ENSBTAG00000012444_ADAM12

ENSBTAG00000003886_BSP3

ENSBTAG00000048004

ENSBTAG00000018566_SFRP5

ENSBTAG00000002427

ENSBTAG00000045966_GPRIN3

ENSBTAG00000040060_ARTN

ENSBTAG00000035392

ENSBTAG00000010241_UNC5D

ENSBTAG00000010283

ENSBTAG00000045715

ENSBTAG00000046586

ENSBTAG00000002865_KCNIP1

ENSBTAG00000004292_CRMP1

ENSBTAG00000045655

ENSBTAG00000016913_AVIL

ENSBTAG00000016765_C7orf61

ENSBTAG00000020159_NKAIN1

ENSBTAG00000048048

ENSBTAG00000045879_FAM26E

ENSBTAG00000039046_MGC127538

ENSBTAG00000011706_TNR

ENSBTAG00000013686_WSCD2

ENSBTAG00000036099

ENSBTAG00000012275_ENDOU

ENSBTAG00000008687_PCSK2

ENSBTAG00000012280_PGLYRP2

ENSBTAG00000033299_IGFALS

ENSBTAG00000038738_PXMP4

ENSBTAG00000045571

ENSBTAG00000014498_UNC93A

ENSBTAG00000019762

ENSBTAG00000006624_SLC36A3

ENSBTAG00000048147

ENSBTAG00000012971

ENSBTAG00000048002

ENSBTAG00000002545

ENSBTAG00000006110_MCTP1

ENSBTAG00000046123_UTY

ENSBTAG00000043402_SNORA43

ENSBTAG00000009900

ENSBTAG00000026418_HBA

ENSBTAG00000045649_DEFB

ENSBTAG00000045563

ENSBTAG00000029857_bta-mir-145

ENSBTAG00000010223_NTF3

ENSBTAG00000017146_GRIK4

ENSBTAG00000025213_OMG

ENSBTAG00000005143

ENSBTAG00000003278_HOXC10

ENSBTAG00000000011_TDH

ENSBTAG00000001803_FHL5

ENSBTAG00000048177

ENSBTAG00000023270_CDH19

ENSBTAG00000025621

ENSBTAG00000023431

ENSBTAG00000048258

ENSBTAG00000017102

ENSBTAG00000017785_FRMPD4

ENSBTAG00000045912

ENSBTAG00000025009_HOXB9

ENSBTAG00000034597

ENSBTAG00000009280

ENSBTAG00000005604_POPDC2

ENSBTAG00000045633_AGTR1

ENSBTAG00000046051

ENSBTAG00000001836

ENSBTAG00000002008

ENSBTAG00000011638

ENSBTAG00000011023_TMEM52B

ENSBTAG00000016059

ENSBTAG00000045579_TMEM60

ENSBTAG00000019892_HAS2

ENSBTAG00000005504_TP53INP2

ENSBTAG00000021066_CAPN11

ENSBTAG00000046147

ENSBTAG00000048085

ENSBTAG00000026417_HBA

ENSBTAG00000016216

ENSBTAG00000031497_FGFBP1

ENSBTAG00000024490_ULBP27

ENSBTAG00000003472_ABCA12

ENSBTAG00000021120_SMYD1

ENSBTAG00000014218_DKK1

ENSBTAG00000016505_TMC7

ENSBTAG00000027274

ENSBTAG00000043972_SLC24A2

ENSBTAG00000039357

ENSBTAG00000040413_CES3

ENSBTAG00000009296_SLC26A5

ENSBTAG00000008511

ENSBTAG00000021707_MYBPC3

ENSBTAG00000039688_FAM101A

ENSBTAG00000038523_ATP1A4

ENSBTAG00000039080_SLC22A3

ENSBTAG00000045987

ENSBTAG00000001078_SRL

ENSBTAG00000047190

ENSBTAG00000030838

ENSBTAG00000011547_KBTBD12

ENSBTAG00000005085_TRIM63

ENSBTAG00000022971

ENSBTAG00000046186

ENSBTAG00000023249

ENSBTAG00000021987_GPX5

ENSBTAG00000019628_EDAR

ENSBTAG00000003343

ENSBTAG00000048067_C17orf96

ENSBTAG00000006233_NIM1K

ENSBTAG00000011390_CHRND

ENSBTAG00000039834

ENSBTAG00000019385_SCN3A

ENSBTAG00000040390

ENSBTAG00000046926_GPX4

ENSBTAG00000025220_FAM155A

ENSBTAG00000047863

ENSBTAG00000037743

ENSBTAG00000022990

ENSBTAG00000021959_CDC20B

ENSBTAG00000012290_SCNN1B

ENSBTAG00000039169

ENSBTAG00000046769

ENSBTAG00000010555_LSMEM1

ENSBTAG00000023000

ENSBTAG00000018253_CHRNA1

ENSBTAG00000047869

ENSBTAG00000047814

ENSBTAG00000014127_PGHS-2

ENSBTAG00000035349

ENSBTAG00000026242_BAALC

ENSBTAG00000018311

ENSBTAG00000001249_SULT1B1

ENSBTAG00000026543

ENSBTAG00000003253_NPPC

ENSBTAG00000047871

ENSBTAG00000002066_MYL7

ENSBTAG00000000426

ENSBTAG00000000743_MBOAT4

ENSBTAG00000045555

ENSBTAG00000017677_SCG3

ENSBTAG00000006655_PIP

ENSBTAG00000000719

ENSBTAG00000026323_LYSB

ENSBTAG00000017949

ENSBTAG00000007446_NGF

ENSBTAG00000025307

ENSBTAG00000045994

ENSBTAG00000016071_HHIP

ENSBTAG00000013991_NR2E1

ENSBTAG00000023182_WNK3

ENSBTAG00000040296_GPR21

ENSBTAG00000007199

ENSBTAG00000003600_MGC151839

ENSBTAG00000024957_SNCA

ENSBTAG00000047320_UBE2D3

ENSBTAG00000020049_CABP1

ENSBTAG00000016158_ACAN

ENSBTAG00000021217_COL11A1

ENSBTAG00000000128_FGF18

ENSBTAG00000046593

ENSBTAG00000031891_NLGN1

ENSBTAG00000047087_MCIDAS

ENSBTAG00000017800_DMRT3

ENSBTAG00000014056_CHRNA6

ENSBTAG00000048089

ENSBTAG00000015351_STYK1

ENSBTAG00000047948

ENSBTAG00000005330_KRTDAP

ENSBTAG00000045671

ENSBTAG00000010640

ENSBTAG00000046234

ENSBTAG00000008351_C12orf60

ENSBTAG00000005290_CLDN2

ENSBTAG00000036258

ENSBTAG00000047823

ENSBTAG00000020582_PRSS22

ENSBTAG00000046776

ENSBTAG00000044492

ENSBTAG00000026768_TH

ENSBTAG00000016754_KLHL38

ENSBTAG00000045821

ENSBTAG00000019158_MEX3A

ENSBTAG00000040450

ENSBTAG00000047638

ENSBTAG00000026122_ZNF804A

ENSBTAG00000013149_NAA11

ENSBTAG00000015940_SCGB2A2

ENSBTAG00000024088

ENSBTAG00000012640_S100A8

ENSBTAG00000045858

ENSBTAG00000007791_KCNQ5

ENSBTAG00000047004_RPA3-AS1

ENSBTAG00000030367_RNF186

ENSBTAG00000017180_SLITRK3

ENSBTAG00000012870_BMP5

ENSBTAG00000047102

ENSBTAG00000039462_PAF

ENSBTAG00000047653

ENSBTAG00000026769_ASCL2

ENSBTAG00000014470_ALDH1L1

ENSBTAG00000040019

ENSBTAG00000034284

ENSBTAG00000006973_PHF21B

ENSBTAG00000046631

ENSBTAG00000038033_KRT6B

ENSBTAG00000040253_ADRA1B

ENSBTAG00000005697

ENSBTAG00000040347_GPC6

ENSBTAG00000045954_LRRC14B

ENSBTAG00000045677

ENSBTAG00000046844

ENSBTAG00000019772_OXTR

ENSBTAG00000008779_ALOX15B

ENSBTAG00000008714_CLSTN2

ENSBTAG00000019658_ASB16

ENSBTAG00000010991_TTR

ENSBTAG00000005750_TCF15

ENSBTAG00000036273

ENSBTAG00000044177_C11orf53

ENSBTAG00000009073

ENSBTAG00000020413_APBA2

ENSBTAG00000023860

ENSBTAG00000039696_SHD

ENSBTAG00000006251

ENSBTAG00000010837_HTR4

ENSBTAG00000001206_LCN15

ENSBTAG00000005262

ENSBTAG00000017679_PAK7

ENSBTAG00000032687

ENSBTAG00000039810

ENSBTAG00000042458_RNase_MRP

ENSBTAG00000047962

ENSBTAG00000048024

ENSBTAG00000000476_THPO

ENSBTAG00000015465_C7H5orf46

ENSBTAG00000003515_SNCG

ENSBTAG00000029828_bta-mir-421

ENSBTAG00000031135_CTXN3

ENSBTAG00000015470_SYPL2

ENSBTAG00000008501_FOXD3

ENSBTAG00000007312_CD209

ENSBTAG00000005772_SPDYC

ENSBTAG00000046716_UBE2D3

ENSBTAG00000047849

ENSBTAG00000020513

ENSBTAG00000010024_MMP24

ENSBTAG00000017194_ASPG

ENSBTAG00000020430_GLT1D1

ENSBTAG00000046102

ENSBTAG00000017946

ENSBTAG00000025458_DPF3

ENSBTAG00000025200_ACCN1

ENSBTAG00000026580_HTR1E

ENSBTAG00000032591_CTHRC1

ENSBTAG00000006977_PLP1

ENSBTAG00000030114_bta-mir-143

ENSBTAG00000040378_HIST1H2AG

ENSBTAG00000003910_SH2D4B

ENSBTAG00000020253_SPEM1

ENSBTAG00000037821

ENSBTAG00000006261_GPR17

ENSBTAG00000038690

ENSBTAG00000030704

ENSBTAG00000019820_RAB9B

ENSBTAG00000032481_DAPL1

ENSBTAG00000014581_MLIP

ENSBTAG00000045569_TADA2B

ENSBTAG00000013877_HORMAD2

ENSBTAG00000038325_CES4A

ENSBTAG00000034676_HIGD1D

ENSBTAG00000011844

ENSBTAG00000000585_LY6G6C

ENSBTAG00000014925_NPAS4

ENSBTAG00000017927_EGFL6

ENSBTAG00000015139

ENSBTAG00000017167_LRAT

ENSBTAG00000043990_KHDRBS2

ENSBTAG00000011537

ENSBTAG00000012320_CETN1

ENSBTAG00000007732_ARPP21

ENSBTAG00000033460_FAM162B

ENSBTAG00000012210_C5

ENSBTAG00000018356_SYT15

ENSBTAG00000047302

ENSBTAG00000010622_TRIM46

ENSBTAG00000009836_CHGA

ENSBTAG00000045695_SLC6A19

ENSBTAG00000012918_CRISP3

ENSBTAG00000010175_COL8A2

ENSBTAG00000033506

ENSBTAG00000046168

ENSBTAG00000047331_GYPB

ENSBTAG00000002240_GPR18

ENSBTAG00000037421

ENSBTAG00000014832_TMEFF2

ENSBTAG00000045577_MCCD1

ENSBTAG00000013537_FER1L6

ENSBTAG00000013711

ENSBTAG00000011010_PRND

ENSBTAG00000003771_C1QL2

ENSBTAG00000010829_EBF2

ENSBTAG00000001912_TRPV5

ENSBTAG00000000986_USH1C

ENSBTAG00000014620_FOXI1

ENSBTAG00000026275_CCL24

ENSBTAG00000048170_PCDHA11

ENSBTAG00000003751_MACC1

ENSBTAG00000021046_ELAVL4

ENSBTAG00000024874

ENSBTAG00000039714

ENSBTAG00000047449

ENSBTAG00000016446_GOT1L1

ENSBTAG00000011743_TLL2

ENSBTAG00000023550

ENSBTAG00000002007_PRG3

ENSBTAG00000034674

ENSBTAG00000001464_GPR156

ENSBTAG00000012682_UNC13A

ENSBTAG00000012097_RSPH6A

ENSBTAG00000005938_BARHL2

ENSBTAG00000000567

ENSBTAG00000011780_SLITRK4

ENSBTAG00000021339_SCN8A

ENSBTAG00000037578

ENSBTAG00000046276

ENSBTAG00000003132_CHRNB4

ENSBTAG00000045495

ENSBTAG00000040144

ENSBTAG00000007638_PIF1

ENSBTAG00000023999_GTF2A1L

ENSBTAG00000046678

ENSBTAG00000017083_TEX15

ENSBTAG00000046635_DDX53

ENSBTAG00000040388

ENSBTAG00000016121_KRT75

ENSBTAG00000008039_ACMSD

ENSBTAG00000010035

ENSBTAG00000017500_KCNK12

ENSBTAG00000006205_DNAJB7

ENSBTAG00000018481

ENSBTAG00000014968_YJEFN3

ENSBTAG00000034091

ENSBTAG00000011579_CHRM1

ENSBTAG00000021462_CSF3

ENSBTAG00000045662_KRT3

ENSBTAG00000020793_KCNA4

ENSBTAG00000047332

ENSBTAG00000014412_ELAVL3

ENSBTAG00000019670_SUCNR1

ENSBTAG00000020737_SOX8

ENSBTAG00000012763

ENSBTAG00000003950_PROC

ENSBTAG00000011392_MYBPC1

ENSBTAG00000033526_GNG13

ENSBTAG00000040386_AT2

ENSBTAG00000024632_LRP1B

ENSBTAG00000008777_ATP4A

ENSBTAG00000033562_LHX1

ENSBTAG00000045086_7SK

ENSBTAG00000034645_PON3

ENSBTAG00000001571_LRRD1

ENSBTAG00000039772_5HTR2B

ENSBTAG00000039803_MYO7B

ENSBTAG00000047228

ENSBTAG00000033173_BHLHE22

ENSBTAG00000048199

ENSBTAG00000033021

ENSBTAG00000005941_TRAV8-1

ENSBTAG00000003353_SLC6A2

ENSBTAG00000002192_C14H8orf46

ENSBTAG00000038464_PLIN5

ENSBTAG00000043297_SNORD20

ENSBTAG00000011321_NUGGC

ENSBTAG00000031238_C16H1ORF14

ENSBTAG00000006354_HP

ENSBTAG00000011598_SOX2

ENSBTAG00000045763

ENSBTAG00000027974

ENSBTAG00000031115

ENSBTAG00000031265_SVOPL

ENSBTAG00000017294_AGP

ENSBTAG00000003921

ENSBTAG00000018707_LDB3

ENSBTAG00000011720_HSD17B6

ENSBTAG00000030672

ENSBTAG00000019293_EGR3

ENSBTAG00000010522

ENSBTAG00000005202_SCP2D1

ENSBTAG00000045932

ENSBTAG00000006408_MROH9

ENSBTAG00000013702_ZNF296

ENSBTAG00000027225_LAP

ENSBTAG00000011654

ENSBTAG00000015409_STK32B

ENSBTAG00000004630_COMP

ENSBTAG00000023309

ENSBTAG00000034368_PRSS33

ENSBTAG00000006569_SEZ6L

ENSBTAG00000021739_NPPB

ENSBTAG00000040065_RAD51AP1

ENSBTAG00000024420_COL28A1

ENSBTAG00000037890

ENSBTAG00000034657

ENSBTAG00000013442_GPR62

ENSBTAG00000046771_FNDC7

ENSBTAG00000006384

ENSBTAG00000007169_P2RX1

ENSBTAG00000004297_ACOXL

ENSBTAG00000016145_ADAMTS19

ENSBTAG00000020467_HRH4

ENSBTAG00000014920_SEMA3E

ENSBTAG00000012184_PTTG1

ENSBTAG00000037526_FABP4

ENSBTAG00000047574

ENSBTAG00000007239_TSG-6

ENSBTAG00000016149_C9

ENSBTAG00000011939_LY6H

ENSBTAG00000021795_HNF1A

ENSBTAG00000007762_ACCN3

ENSBTAG00000047570

ENSBTAG00000045705

ENSBTAG00000029970_bta-mir-196b

ENSBTAG00000017477

ENSBTAG00000002786

ENSBTAG00000010070_GSDMA

ENSBTAG00000034069_MAB21L1

ENSBTAG00000047861

ENSBTAG00000008942_NGEF

ENSBTAG00000002950_TMEM132D

ENSBTAG00000018635_ATP1A3

ENSBTAG00000030416

ENSBTAG00000004903_KRT15

ENSBTAG00000007506_CACNG7

ENSBTAG00000011944_MTTP

ENSBTAG00000018195_CWC15

ENSBTAG00000032880_PRM2

ENSBTAG00000031076

ENSBTAG00000040040_TAS2R40

ENSBTAG00000040282_NOG

ENSBTAG00000015865_CAPN14

ENSBTAG00000047347_RAD51AP1

ENSBTAG00000021220_TMEM72

ENSBTAG00000001392_RDH16

ENSBTAG00000025535_GLYATL3

ENSBTAG00000027080_SLC8A3

ENSBTAG00000011956_CNNM1

ENSBTAG00000018645_DLX5

ENSBTAG00000040255

ENSBTAG00000017641

ENSBTAG00000002145_MMP17

ENSBTAG00000001228_CEND1

ENSBTAG00000017837_GABRB1

ENSBTAG00000011734_ANKRD1

ENSBTAG00000010016_C16H1orf227

ENSBTAG00000017953

ENSBTAG00000045813

ENSBTAG00000038985

ENSBTAG00000032071_SYCP1

ENSBTAG00000040242

ENSBTAG00000019813_ADIPOQ

ENSBTAG00000021407_CASP16

ENSBTAG00000025920

ENSBTAG00000007482

ENSBTAG00000001003_CKMT2

ENSBTAG00000004414_SLC30A10

ENSBTAG00000045039_SCARNA17

ENSBTAG00000008328

ENSBTAG00000027052

ENSBTAG00000048231

ENSBTAG00000012161

ENSBTAG00000005585_ANXA13

ENSBTAG00000047816

ENSBTAG00000017593_TREM1

ENSBTAG00000031788_GSTM1

ENSBTAG00000005850_DLK2

ENSBTAG00000047858

ENSBTAG00000032947_GRXCR1

ENSBTAG00000011952_SULT1E1

ENSBTAG00000019394_ANO5

ENSBTAG00000043968

ENSBTAG00000047952

ENSBTAG00000003301_NCAM2

ENSBTAG00000002024_ASB18

ENSBTAG00000039485_C17orf104

ENSBTAG00000031773_HIST1H3B

ENSBTAG00000046095

ENSBTAG00000022779_OLFM4

ENSBTAG00000010970

ENSBTAG00000047848

ENSBTAG00000019496_CDKN3

ENSBTAG00000023697

ENSBTAG00000039875

ENSBTAG00000007901_EMR1

ENSBTAG00000045592

ENSBTAG00000047412_TNFSF18

ENSBTAG00000003093_TSGA10IP

ENSBTAG00000026156_VGLL3

ENSBTAG00000025108

ENSBTAG00000004095_FAM166A

ENSBTAG00000023967

ENSBTAG00000048203_SAMD13

ENSBTAG00000024326

ENSBTAG00000009266_CD226

ENSBTAG00000025485

ENSBTAG00000033218

ENSBTAG00000007392

ENSBTAG00000047911

ENSBTAG00000022947

ENSBTAG00000013090_NODAL

ENSBTAG00000046544_SLC7A10

ENSBTAG00000017955

ENSBTAG00000002898_UNC45B

ENSBTAG00000016433_FANCB

ENSBTAG00000015170_LCT

ENSBTAG00000048120_7SK

ENSBTAG00000027172

ENSBTAG00000025042

ENSBTAG00000046006_CNR1

ENSBTAG00000005848_PRTG

ENSBTAG00000046554

ENSBTAG00000006415_LRRIQ4

ENSBTAG00000014517_KLB

ENSBTAG00000010273_EREG

ENSBTAG00000005235_DPP10

ENSBTAG00000046560

ENSBTAG00000046063_SNORA3

ENSBTAG00000013334_CSF3R

ENSBTAG00000034174

ENSBTAG00000042863_SNORA7

ENSBTAG00000013219_PEX5L

ENSBTAG00000006295_TSHB

ENSBTAG00000002894_TNFSF4

ENSBTAG00000019504_ADRA1D

ENSBTAG00000007309_ST6GALNAC5

ENSBTAG00000001001_ANKRD55

ENSBTAG00000042543_SNORD113

ENSBTAG00000021616_PRSS57

ENSBTAG00000000360

ENSBTAG00000046948

ENSBTAG00000004224_C19orf38

ENSBTAG00000033545_EBD

ENSBTAG00000002964_TXLNB

ENSBTAG00000021667

ENSBTAG00000021728_OGDHL

ENSBTAG00000006380

ENSBTAG00000005841

ENSBTAG00000014197_ATA3

ENSBTAG00000042434_SNORA66

ENSBTAG00000047873

ENSBTAG00000046418_RNF224

ENSBTAG00000048283

ENSBTAG00000047503

ENSBTAG00000022699_CAV3

ENSBTAG00000001638_FGA

ENSBTAG00000014833_NEU4

ENSBTAG00000048008

ENSBTAG00000046377

ENSBTAG00000040329_HTR1D

ENSBTAG00000040187

ENSBTAG00000000853

ENSBTAG00000005128_VPREB3

ENSBTAG00000014910_PIWIL4

ENSBTAG00000038544

ENSBTAG00000015298_TNFRSF13B

ENSBTAG00000046363

ENSBTAG00000044853_snoU85

ENSBTAG00000007859_VSIG1

ENSBTAG00000001326_SLC44A5

ENSBTAG00000012403_ARG1

ENSBTAG00000048036

ENSBTAG00000039618_C10H11ORF46

ENSBTAG00000021492_FAM163A

ENSBTAG00000007333_AKNAD1

ENSBTAG00000020856_HFM1

ENSBTAG00000011624_FGF21

ENSBTAG00000030083_bta-mir-670

ENSBTAG00000001603_YIPF7

ENSBTAG00000039817

ENSBTAG00000014683_APOBEC1

ENSBTAG00000015985_GPR20

ENSBTAG00000047757

ENSBTAG00000026610_SMCO3

ENSBTAG00000000522_AHSG

ENSBTAG00000027786

ENSBTAG00000005450_CYP3A5

ENSBTAG00000006841_LRRC4B

ENSBTAG00000048291

ENSBTAG00000002414_KCNJ10

ENSBTAG00000000169_ASZ1

ENSBTAG00000005129_CEP55

ENSBTAG00000040590

ENSBTAG00000015260

ENSBTAG00000017391_HS3ST2

ENSBTAG00000048104

ENSBTAG00000038204

ENSBTAG00000000229

ENSBTAG00000016030_HOXD10

ENSBTAG00000035438

ENSBTAG00000003272

ENSBTAG00000005176_GDNF

ENSBTAG00000046412

ENSBTAG00000007316_NDST3

ENSBTAG00000038361_SERPINA11

ENSBTAG00000010177_GOLT1A

ENSBTAG00000008623_FGF3

ENSBTAG00000021050_ECEL1

ENSBTAG00000039016

ENSBTAG00000032455_H2B

ENSBTAG00000047940

ENSBTAG00000015654_PON1

ENSBTAG00000044050_XKR4

ENSBTAG00000011336_OTOGL

ENSBTAG00000029886_bta-mir-214

ENSBTAG00000014908_CCL27

ENSBTAG00000045990

ENSBTAG00000000354_PDE6G

ENSBTAG00000021139

ENSBTAG00000001495_SPINLW1

ENSBTAG00000020843_PCSK1

ENSBTAG00000000516_OTOR

ENSBTAG00000015197_KLHDC8A

ENSBTAG00000015033

ENSBTAG00000018167_KLHL31

ENSBTAG00000009938_XCL1

ENSBTAG00000008819_BTBD16

ENSBTAG00000048196_SLC25A16

ENSBTAG00000042679_SNORD113

ENSBTAG00000018326_FXYD7

ENSBTAG00000023954

ENSBTAG00000047397

ENSBTAG00000022588_C14H8orf34

ENSBTAG00000032709_CMTM5

ENSBTAG00000039397_WNT3A

ENSBTAG00000012436_HES7

ENSBTAG00000039652_PTGDR2

ENSBTAG00000031250

ENSBTAG00000038326

ENSBTAG00000036430_bta-mir-29d

ENSBTAG00000001696_RBM46

ENSBTAG00000037570

ENSBTAG00000026977_PTPRQ

ENSBTAG00000025088_RCVRN

ENSBTAG00000030423_CLEC1B

ENSBTAG00000046524_snoZ17

ENSBTAG00000012560_SFTPC

ENSBTAG00000009433_BGLAP

ENSBTAG00000039522_CYCT

ENSBTAG00000011062_TDO2

ENSBTAG00000003327_MGC157327

ENSBTAG00000001706_SLC9A2

ENSBTAG00000046790

ENSBTAG00000004212_ACTBL2

ENSBTAG00000007010

ENSBTAG00000015576_NPAS1

ENSBTAG00000047377

ENSBTAG00000018575_TRHDE

ENSBTAG00000014738_HOXA11

ENSBTAG00000013741_CPLX2

ENSBTAG00000038849_TRDN

ENSBTAG00000006042_C10orf128

ENSBTAG00000000119

ENSBTAG00000032396_KCTD19

ENSBTAG00000015830_SLC5A5

ENSBTAG00000046355

ENSBTAG00000038377_ADAM20

ENSBTAG00000019355_KCNK10

ENSBTAG00000016885_LRRTM3

ENSBTAG00000011871_BTLA

ENSBTAG00000039491

ENSBTAG00000047399

ENSBTAG00000030898_MS4A13

ENSBTAG00000039171_ADAM7

ENSBTAG00000045010_bta-mir-2350

ENSBTAG00000015895_HGD

ENSBTAG00000048061_GRM5

ENSBTAG00000024751

ENSBTAG00000018885

ENSBTAG00000035206

ENSBTAG00000011441

ENSBTAG00000016385_F10

ENSBTAG00000012207

ENSBTAG00000009252_KLRA1

ENSBTAG00000000395

ENSBTAG00000044406_SCARNA15

ENSBTAG00000034555_AP3S1

ENSBTAG00000047224_RYK

ENSBTAG00000001062_PTH2

ENSBTAG00000003625_VSTM2A

ENSBTAG00000031364_NAT8L

ENSBTAG00000039963

ENSBTAG00000017650_TMPRSS5

ENSBTAG00000000861_ETV3L

ENSBTAG00000018778_TMC1

ENSBTAG00000021360_RASGEF1C

ENSBTAG00000001623

ENSBTAG00000006263_GP2

ENSBTAG00000002355

ENSBTAG00000037586

ENSBTAG00000012553

ENSBTAG00000019447_NPVF

ENSBTAG00000008479_CXCL13

ENSBTAG00000016620_SIGLEC15

ENSBTAG00000010605_VAX2

ENSBTAG00000046472

ENSBTAG00000046749

ENSBTAG00000004519

ENSBTAG00000000965_TNNI3K

ENSBTAG00000046584

ENSBTAG00000009722

ENSBTAG00000043953

ENSBTAG00000042775_SNORD103

ENSBTAG00000007558_IL9R

ENSBTAG00000044275_SNORA31

ENSBTAG00000039245_GK2

ENSBTAG00000001297

ENSBTAG00000021092_ATP6V0D2

ENSBTAG00000011496

ENSBTAG00000042780_SNORD91

ENSBTAG00000003528

ENSBTAG00000006709_NPPA

ENSBTAG00000044894_Vault

ENSBTAG00000007295

ENSBTAG00000048118

ENSBTAG00000015784_HAPLN2

ENSBTAG00000003838

ENSBTAG00000019199_MGC133647

ENSBTAG00000047719_DRD1

ENSBTAG00000033699_CHRM4

ENSBTAG00000001213

ENSBTAG00000042406_SNORD18

ENSBTAG00000014572

ENSBTAG00000006674_SLC13A1

ENSBTAG00000017305_VPREB

ENSBTAG00000016138_CGA

ENSBTAG00000019532_HTRA4

ENSBTAG00000034503

ENSBTAG00000031766_HIST1H3H

ENSBTAG00000001865_SGCG

ENSBTAG00000042224_SNORA19

ENSBTAG00000047312

ENSBTAG00000001979_TAF7L

ENSBTAG00000000419_PHEX

ENSBTAG00000043480_SNORD14

ENSBTAG00000024582_RPL10L

ENSBTAG00000014930_MYLK2

ENSBTAG00000011587_C10orf90

ENSBTAG00000014287

ENSBTAG00000040023

ENSBTAG00000046456_IL6

ENSBTAG00000009468_FAM159B

ENSBTAG00000021508_LMOD3

ENSBTAG00000039325_GPA

ENSBTAG00000030798_WFDC11

ENSBTAG00000046784

ENSBTAG00000007063_GPHA2

ENSBTAG00000010947_PHYHIPL

ENSBTAG00000001344_METTL21E

ENSBTAG00000039850

ENSBTAG00000011271_SNX31

ENSBTAG00000002717_INA

ENSBTAG00000013791_GPR41

ENSBTAG00000004888_SLC10A4

ENSBTAG00000045841

ENSBTAG00000015676_AMBP

ENSBTAG00000000888_SPTA1

ENSBTAG00000032106_PPAPDC1A

ENSBTAG00000003467_SLC5A11

ENSBTAG00000029863_bta-mir-208a

ENSBTAG00000012217_PLA2G2F

ENSBTAG00000001382_SLC26A9

ENSBTAG00000009382_KLK13

ENSBTAG00000022861_KLRJ1

ENSBTAG00000018237_MYO16

ENSBTAG00000001825_SP6

ENSBTAG00000015759

ENSBTAG00000011804_ARNTL2

ENSBTAG00000014899_TTC36

ENSBTAG00000021240_DCSTAMP

ENSBTAG00000031918

ENSBTAG00000044409_SNORA70

ENSBTAG00000047153

ENSBTAG00000002822_CNBD2

ENSBTAG00000015347_WNT10B

ENSBTAG00000039675

ENSBTAG00000038605

ENSBTAG00000001703_SYT13

ENSBTAG00000011621_IZUMO1

ENSBTAG00000004109

ENSBTAG00000020300

ENSBTAG00000021426_CPLX3

ENSBTAG00000027348_OOSP1

ENSBTAG00000033195

ENSBTAG00000046788

ENSBTAG00000026633

ENSBTAG00000003992_LXH4

ENSBTAG00000046807

ENSBTAG00000030517

ENSBTAG00000021059_TNMD

ENSBTAG00000048317

ENSBTAG00000006036_ERP27

ENSBTAG00000015854_TCHHL1

ENSBTAG00000003260_GPR151

ENSBTAG00000002089_FAM217A

ENSBTAG00000013002_GABRQ

ENSBTAG00000014580_FCRLB

ENSBTAG00000021101_GPR88

ENSBTAG00000009774_MOBP

ENSBTAG00000013027_CCK

ENSBTAG00000005337_RAB3B

ENSBTAG00000016186

ENSBTAG00000044375_SNORA70

ENSBTAG00000046175

ENSBTAG00000030483_KLK7

ENSBTAG00000042626_SNORD22

ENSBTAG00000014735_HOXA13

ENSBTAG00000006188_USH2A

ENSBTAG00000038183

ENSBTAG00000038138

ENSBTAG00000047690

ENSBTAG00000032782_INSC

ENSBTAG00000020140_CER1

ENSBTAG00000038232

ENSBTAG00000046714

ENSBTAG00000016998_ARL11

ENSBTAG00000016645_GABRA4

ENSBTAG00000025202

ENSBTAG00000001764_NCAN

ENSBTAG00000040605_FOXL1

ENSBTAG00000003564_HPX

ENSBTAG00000047688

ENSBTAG00000024132_SMC1B

ENSBTAG00000000044_MYADML2

ENSBTAG00000018218

ENSBTAG00000001784_LDLRAD2

ENSBTAG00000035785

ENSBTAG00000045537

ENSBTAG00000002934_GPR115

ENSBTAG00000022471_TTPA

ENSBTAG00000046876

ENSBTAG00000007794_KRT20

ENSBTAG00000015935_IYD

ENSBTAG00000015737_KPNA7

ENSBTAG00000045860

ENSBTAG00000046336

ENSBTAG00000045283_snoZ6

ENSBTAG00000029885_bta-mir-450a-2

ENSBTAG00000042357_SNORD21

ENSBTAG00000038090

ENSBTAG00000019159_PVALB

ENSBTAG00000047932

ENSBTAG00000012057_GIF

ENSBTAG00000006173_CCDC116

ENSBTAG00000031256

ENSBTAG00000020353_PFN3

ENSBTAG00000046165

ENSBTAG00000045866

ENSBTAG00000044161_NMU

ENSBTAG00000008644_KCNK4

ENSBTAG00000013977_PRSS38

ENSBTAG00000006191_SERPINA12

ENSBTAG00000042134_SNORD14

ENSBTAG00000010728_RAB44

ENSBTAG00000018161_TBX18

ENSBTAG00000029957_bta-mir-191

ENSBTAG00000024291_FAM71F2

ENSBTAG00000019547

ENSBTAG00000009675

ENSBTAG00000044478_bta-mir-2442

ENSBTAG00000048245

ENSBTAG00000009701_IL25

ENSBTAG00000017026_DEPDC1B

ENSBTAG00000030296_LCNL1

ENSBTAG00000037768_MMP3

ENSBTAG00000019840

ENSBTAG00000046421_SFTPD

ENSBTAG00000012025_LMX1A

ENSBTAG00000046725_TNNC2

ENSBTAG00000038606_DBDR

ENSBTAG00000000171

ENSBTAG00000037993

ENSBTAG00000003760

ENSBTAG00000019041_FAM19A1

ENSBTAG00000025340_CDHR2

ENSBTAG00000006725

ENSBTAG00000044068_VWC2

ENSBTAG00000008505_APOB

ENSBTAG00000027419

ENSBTAG00000009180_NRSN1

ENSBTAG00000007887_GRIN2A

ENSBTAG00000045805

ENSBTAG00000034294

ENSBTAG00000015150_IL12A

ENSBTAG00000001099_C5H12ORF53

ENSBTAG00000034854_GRIK1

ENSBTAG00000030199

ENSBTAG00000031889_H2B

ENSBTAG00000015274_PRL

ENSBTAG00000048145

ENSBTAG00000010112_BPIFB6

ENSBTAG00000014741_OTX2

ENSBTAG00000026259

ENSBTAG00000047368

ENSBTAG00000014704_HELT

ENSBTAG00000005287_CYP7A1

ENSBTAG00000013979

ENSBTAG00000021397_NKX2-3

ENSBTAG00000002016

ENSBTAG00000004130_C11orf87

ENSBTAG00000010615_ARL9

ENSBTAG00000030922

ENSBTAG00000033954_PRPS1L1

ENSBTAG00000009750_RAX2

ENSBTAG00000034925_KHDC3L

ENSBTAG00000011672_GABRR1

ENSBTAG00000004955_SYTL5

ENSBTAG00000026917

ENSBTAG00000045829_AZU1

ENSBTAG00000026070

ENSBTAG00000008832_CCL1

ENSBTAG00000012502

ENSBTAG00000018268_UCN

ENSBTAG00000040103_SYT8

ENSBTAG00000042256_SNORA74

ENSBTAG00000002855

ENSBTAG00000030504_HIST1H4D

ENSBTAG00000046949_RPS26

ENSBTAG00000037794_MYH4

ENSBTAG00000042234_SNORA70

ENSBTAG00000010796_SPADH1

ENSBTAG00000047741

ENSBTAG00000026250_PDHA2

ENSBTAG00000046540_SERPINA3-1

ENSBTAG00000022534

ENSBTAG00000011113

ENSBTAG00000016483

ENSBTAG00000009030_NOX5

ENSBTAG00000046236

ENSBTAG00000006352_MYT1

ENSBTAG00000047652_RBMX

ENSBTAG00000006221_GPR97

ENSBTAG00000032428

ENSBTAG00000038027

ENSBTAG00000011036

ENSBTAG00000031462_FAM209B

ENSBTAG00000012319_KCNE1L

ENSBTAG00000038267

ENSBTAG00000042711_snoU2_19

ENSBTAG00000035696_UCN3

ENSBTAG00000011500_CASQ2

ENSBTAG00000017502_RIMKLA

ENSBTAG00000031376_BSP30C

ENSBTAG00000006473

ENSBTAG00000038080

ENSBTAG00000013700

ENSBTAG00000030306_U2

ENSBTAG00000020120_SEL1L2

ENSBTAG00000000930

ENSBTAG00000009702_MYHC-FETAL

ENSBTAG00000031614_BOLL

ENSBTAG00000046993

ENSBTAG00000038397

ENSBTAG00000047595

ENSBTAG00000012888_C2H2orf80

ENSBTAG00000032853

ENSBTAG00000007286

ENSBTAG00000007298_TEX29

ENSBTAG00000024895

ENSBTAG00000042739_SNORD113

ENSBTAG00000043521_SNORA70

ENSBTAG00000044837_SCARNA21

ENSBTAG00000046279

ENSBTAG00000014078_IGFBPL1

ENSBTAG00000020849

ENSBTAG00000012014_CDX1

ENSBTAG00000029836_bta-mir-30f

ENSBTAG00000042269_U6atac

ENSBTAG00000047146

ENSBTAG00000010284_ETNPPL

ENSBTAG00000008945_SDSL

ENSBTAG00000047841

ENSBTAG00000045409_bta-mir-2487

ENSBTAG00000001150_KCNE1

ENSBTAG00000045275_SCARNA7

ENSBTAG00000010385

ENSBTAG00000046443_IFNAA

ENSBTAG00000048094

ENSBTAG00000008394_MYL3

ENSBTAG00000048121_SNORD116

ENSBTAG00000047899

ENSBTAG00000042852_SNORA43

ENSBTAG00000038189_GPR52

ENSBTAG00000026273_MYL10

ENSBTAG00000025246_ZIC2

ENSBTAG00000047264

ENSBTAG00000040504_TRAV16

ENSBTAG00000046947

ENSBTAG00000016864_LBP

ENSBTAG00000007969_CIDEC

ENSBTAG00000046132

ENSBTAG00000043016_snR47

ENSBTAG00000047893

ENSBTAG00000046702_SNORA67

ENSBTAG00000008148_GCM-1

ENSBTAG00000025951

ENSBTAG00000017924

ENSBTAG00000047266

ENSBTAG00000048096

ENSBTAG00000020508

ENSBTAG00000007094_KIAA1024

ENSBTAG00000029763_bta-mir-375

ENSBTAG00000048244

ENSBTAG00000008117_SLC5A4

ENSBTAG00000045659

ENSBTAG00000031632_ADRA1A

ENSBTAG00000000400_KRT85

ENSBTAG00000000997

ENSBTAG00000015702_PITX3

ENSBTAG00000043177_SNORA16

ENSBTAG00000018085_GTSF1

ENSBTAG00000023507

ENSBTAG00000004702

ENSBTAG00000039691

ENSBTAG00000025847

ENSBTAG00000013134_SOST

ENSBTAG00000045635

ENSBTAG00000042898_snoU2-30

ENSBTAG00000047305

ENSBTAG00000012166_TMC3

ENSBTAG00000039355_MADCAM1

ENSBTAG00000014834_ZP3

ENSBTAG00000000164_GNRH1

ENSBTAG00000011750

ENSBTAG00000028153_5S_rRNA

ENSBTAG00000045793

ENSBTAG00000014045

ENSBTAG00000019087_C16orf95

ENSBTAG00000039891_KRT71

ENSBTAG00000015349_C1orf56

ENSBTAG00000016209_SLURP1

ENSBTAG00000009536_RNF151

ENSBTAG00000046911_SCN10A

ENSBTAG00000002565

ENSBTAG00000043103_SNORA8

ENSBTAG00000006194_FOSL1

ENSBTAG00000001247_LECT2

ENSBTAG00000046342

ENSBTAG00000009674_C3H1orf185

ENSBTAG00000022019

ENSBTAG00000027841

ENSBTAG00000047068

ENSBTAG00000047013

ENSBTAG00000032350

ENSBTAG00000045404_SNORA79

ENSBTAG00000029818_bta-mir-224

ENSBTAG00000010051_LGSN

ENSBTAG00000031655

ENSBTAG00000017054

ENSBTAG00000043153_U6atac

ENSBTAG00000013473_MGC127766

ENSBTAG00000030413_LACTBL1

ENSBTAG00000003320_GLRA4

ENSBTAG00000032833

ENSBTAG00000022744_TRIM71

ENSBTAG00000006343_IL1R2

ENSBTAG00000002582_LYG2

ENSBTAG00000046849_IFNB3

ENSBTAG00000002887_DDI1

ENSBTAG00000029867_bta-mir-15a

ENSBTAG00000032926

ENSBTAG00000005855

ENSBTAG00000001530_FGF8

ENSBTAG00000035122

ENSBTAG00000040083

ENSBTAG00000007767_TBX15

ENSBTAG00000020788_SLC18A1

ENSBTAG00000038739_CPLX4

ENSBTAG00000007843_ITIH1

ENSBTAG00000024929_PPP1R27

ENSBTAG00000045770_KRT77

ENSBTAG00000026861

ENSBTAG00000032237_PNLIP

ENSBTAG00000040586

ENSBTAG00000039356

ENSBTAG00000021458_DLX6

ENSBTAG00000042143_SNORD105

ENSBTAG00000045830

ENSBTAG00000005425_GATA4

ENSBTAG00000007009

ENSBTAG00000005471_RHCG

ENSBTAG00000024043

ENSBTAG00000048052

ENSBTAG00000020242_CRLF2

ENSBTAG00000004206_LRRC55

ENSBTAG00000039765

ENSBTAG00000004607_GSG1L

ENSBTAG00000046083_ZNF280A

ENSBTAG00000008676_SRRM4

ENSBTAG00000018369_MYL2

ENSBTAG00000000214

ENSBTAG00000036389_bta-mir-16a

ENSBTAG00000043928_SNORA79

ENSBTAG00000010330_LYZL1

ENSBTAG00000009144

ENSBTAG00000047562

ENSBTAG00000005609

ENSBTAG00000034189

ENSBTAG00000038069_FAM188B2

ENSBTAG00000039956

ENSBTAG00000046352

ENSBTAG00000015460_TP63

ENSBTAG00000042723_U11

ENSBTAG00000005798_TRIM50

ENSBTAG00000046368

ENSBTAG00000040157

ENSBTAG00000045487

ENSBTAG00000002247

ENSBTAG00000014072

ENSBTAG00000009220

ENSBTAG00000016662_CPS1

ENSBTAG00000018180_C12orf50

ENSBTAG00000047404

ENSBTAG00000020723

ENSBTAG00000013632_GRM4

ENSBTAG00000002775_C12orf71

ENSBTAG00000012285_ESRRB

ENSBTAG00000048046

ENSBTAG00000003497

ENSBTAG00000039559

ENSBTAG00000006733_PPP1R3A

ENSBTAG00000016097_CACNA1F

ENSBTAG00000045112_bta-mir-2398

ENSBTAG00000044899_bta-mir-2460

ENSBTAG00000036304_CNGB3

ENSBTAG00000035054_COL9A1

ENSBTAG00000040357_RNF12

ENSBTAG00000014251_MMP20

ENSBTAG00000021914

ENSBTAG00000047745

ENSBTAG00000042126_SNORA66

ENSBTAG00000044157_FAM71E2

ENSBTAG00000038858_CKLF

ENSBTAG00000012628_SLC1A2

ENSBTAG00000011783

ENSBTAG00000013481_CYP4A22

ENSBTAG00000046143

ENSBTAG00000037637_5_8S_rRNA

ENSBTAG00000017145_C1orf177

ENSBTAG00000047105

ENSBTAG00000019168_PHOX2A

ENSBTAG00000046932

ENSBTAG00000046240

ENSBTAG00000045544

ENSBTAG00000047872_KRT84

ENSBTAG00000032114

ENSBTAG00000037810

ENSBTAG00000001866_EMX1

ENSBTAG00000023032_SFTPA1

ENSBTAG00000046039

ENSBTAG00000004608

ENSBTAG00000009868_VGF

ENSBTAG00000045335_bta-mir-2286

ENSBTAG00000048018

ENSBTAG00000019799_FCAMR

ENSBTAG00000001610_BKB3

ENSBTAG00000016867_HSD17B3

ENSBTAG00000017345_KIF2B

ENSBTAG00000038748_HBB

ENSBTAG00000038312_MS4A18

ENSBTAG00000033477

ENSBTAG00000021994_CACNA2D4

ENSBTAG00000026034

ENSBTAG00000019391

ENSBTAG00000045819

ENSBTAG00000008871_DDX4

ENSBTAG00000020191_FEV

ENSBTAG00000008343

ENSBTAG00000046868

ENSBTAG00000006620_SLC24A4

ENSBTAG00000003492

ENSBTAG00000044547_SNORA27

ENSBTAG00000047107_TNIP3

ENSBTAG00000005930

ENSBTAG00000038630_KLHL34

ENSBTAG00000032884_TNP2

ENSBTAG00000001998_ST6GALNAC1

ENSBTAG00000006594_TMEM211

ENSBTAG00000012896_METTL7B

ENSBTAG00000044508_U6

ENSBTAG00000024611

ENSBTAG00000034315

ENSBTAG00000013907_CRP

ENSBTAG00000044624_U7

ENSBTAG00000030297_MGC137099

ENSBTAG00000003060_NTRK1

ENSBTAG00000034582_H2B

ENSBTAG00000040337

ENSBTAG00000038149_NLRP12

ENSBTAG00000047257

ENSBTAG00000007736

ENSBTAG00000029683_U1

ENSBTAG00000045650

ENSBTAG00000045996

ENSBTAG00000026365

ENSBTAG00000010215_CREB3L3

ENSBTAG00000039702_FCRL3

ENSBTAG00000043446_SNORA51

ENSBTAG00000031955_GPR26

ENSBTAG00000004793_AMBN

ENSBTAG00000044771_5S_rRNA

ENSBTAG00000043339_U6

ENSBTAG00000018759

1. **Cluster 2: 182 genes.**


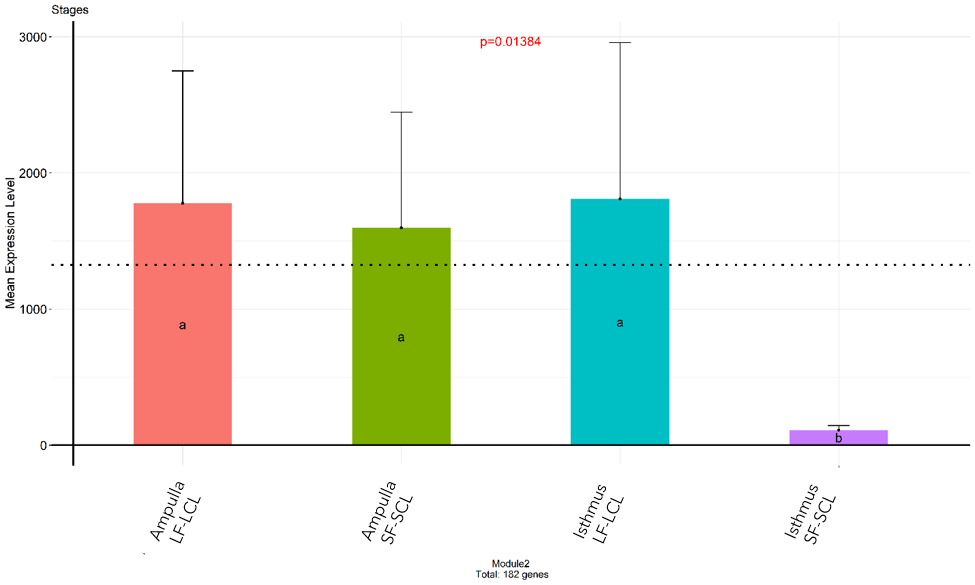


ENSBTAG00000005973_OVGP1

ENSBTAG00000014713_RARRES1

ENSBTAG00000024849

ENSBTAG00000000177_MSLN

ENSBTAG00000037693_MUC16

ENSBTAG00000007431_KIAA1199

ENSBTAG00000007496_P2RX2

ENSBTAG00000001801_SYT4

ENSBTAG00000021190_CDH2

ENSBTAG00000039446

ENSBTAG00000002199_CORIN

ENSBTAG00000046375

ENSBTAG00000038384_KRT5

ENSBTAG00000001417_ACSM1

ENSBTAG00000018650_HEPACAM

ENSBTAG00000006730_SUSD2

ENSBTAG00000003829_HPN

ENSBTAG00000038878

ENSBTAG00000005268_PLA2G4D

ENSBTAG00000015345_BNIPL

ENSBTAG00000002110_DMGDH

ENSBTAG00000018160_LAMA1

ENSBTAG00000014885_MYOM3

ENSBTAG00000002430_COL17A1

ENSBTAG00000009798_DCDC2

ENSBTAG00000002974_FMO2

ENSBTAG00000012992_CDH6

ENSBTAG00000045520_PDYN

ENSBTAG00000038974_STMN3

ENSBTAG00000011817_GABRA2

ENSBTAG00000012119_PTPRZ1

ENSBTAG00000033386_C12H13ORF26

ENSBTAG00000017576_GPR144

ENSBTAG00000025101_ASGR2

ENSBTAG00000026247_PKHD1L1

ENSBTAG00000004578_LGI3

ENSBTAG00000044148_UTS2B

ENSBTAG00000016407_IRX6

ENSBTAG00000011820_DAPK2

ENSBTAG00000019855_MAP3K15

ENSBTAG00000020813

ENSBTAG00000009837_ANKS4B

ENSBTAG00000014829_MYRFL

ENSBTAG00000023549_CYP2C19

ENSBTAG00000047225_BCL2L14

ENSBTAG00000000066_LRRN4

ENSBTAG00000001558_PATL2

ENSBTAG00000001851

ENSBTAG00000021529

ENSBTAG00000005527_GIPR

ENSBTAG00000047510_SMIM6

ENSBTAG00000018657_ATP13A5

ENSBTAG00000031679_HSD17B13

ENSBTAG00000008773_GUCY2D

ENSBTAG00000026710_C2orf72

ENSBTAG00000000690_THEM5

ENSBTAG00000001102_SERPINB10

ENSBTAG00000002963_SAA4

ENSBTAG00000017376_GALNT5

ENSBTAG00000012462_MAP1LC3C

ENSBTAG00000020966_ALX3

ENSBTAG00000040460_KRT23

ENSBTAG00000027017_SIX3

ENSBTAG00000009294_DEGS2

ENSBTAG00000033554_TRAPPC3L

ENSBTAG00000044171_CNIH3

ENSBTAG00000020203_TMEM151A

ENSBTAG00000001255_CRYBB3

ENSBTAG00000023198_SERPINB2

ENSBTAG00000021588_SCG2

ENSBTAG00000033122_FOXD4L1

ENSBTAG00000013619_SCTR

ENSBTAG00000009864_B4GALNT2

ENSBTAG00000030994_ANKS1B

ENSBTAG00000046353

ENSBTAG00000015547_SLC26A3

ENSBTAG00000012475_ALOX12B

ENSBTAG00000032881_SLCO5A1

ENSBTAG00000000219_GRIN2B

ENSBTAG00000003006_RNASE9

ENSBTAG00000013578_CHI3L2

ENSBTAG00000044197_EDARADD

ENSBTAG00000032560_SPINK2

ENSBTAG00000046376

ENSBTAG00000012706_RTP1

ENSBTAG00000007262_ZNF536

ENSBTAG00000046058_RNASE11

ENSBTAG00000018287_SLC25A48

ENSBTAG00000026792

ENSBTAG00000008788_SLC13A2

ENSBTAG00000013124_LRTM1

ENSBTAG00000002369_TMEM217

ENSBTAG00000007904_KRT72

ENSBTAG00000010847_FOXN4

ENSBTAG00000003666_ZPLD1

ENSBTAG00000031194_PHLDA2

ENSBTAG00000016202_TREH

ENSBTAG00000000170

ENSBTAG00000007503_STRC

ENSBTAG00000021408_FMO1

ENSBTAG00000024773_POU3F2

ENSBTAG00000012229

ENSBTAG00000001187_RASL10A

ENSBTAG00000047563_CLDN9

ENSBTAG00000019535

ENSBTAG00000029853_bta-let-7d

ENSBTAG00000031933_ALOX12E

ENSBTAG00000048078

ENSBTAG00000004662_SLC16A12

ENSBTAG00000047990

ENSBTAG00000005741_DLX2

ENSBTAG00000020597_FMO3

ENSBTAG00000007359_PROP1

ENSBTAG00000047704_NPBWR2

ENSBTAG00000004561_PAX6

ENSBTAG00000031647_LRG1

ENSBTAG00000012140_GPR128

ENSBTAG00000016777_TEX101

ENSBTAG00000048080

ENSBTAG00000046383

ENSBTAG00000015533_PTCHD2

ENSBTAG00000011525_SMCT1

ENSBTAG00000001765_HAPLN4

ENSBTAG00000003864_SLC13A4

ENSBTAG00000038168_SLITRK1

ENSBTAG00000012794_PAH

ENSBTAG00000034372

ENSBTAG00000011163_CYTL1

ENSBTAG00000038307

ENSBTAG00000044548_bta-mir-2284b

ENSBTAG00000021189_ARHGAP36

ENSBTAG00000020676_MMP9

ENSBTAG00000034954_DEFB5

ENSBTAG00000012801_UPP2

ENSBTAG00000039234_LIPK

ENSBTAG00000046280_C2orf16

ENSBTAG00000005122_KNG1

ENSBTAG00000046638_IFNAH

ENSBTAG00000047218

ENSBTAG00000002498

ENSBTAG00000038415_SLC6A12

ENSBTAG00000045630

ENSBTAG00000039256

ENSBTAG00000029906_bta-mir-133a-2

ENSBTAG00000026344_MAFA

ENSBTAG00000020543_UPK1A

ENSBTAG00000039893_OR9I1

ENSBTAG00000030629_MGC127133

ENSBTAG00000046457

ENSBTAG00000017628_MAP7D2

ENSBTAG00000032918_SCGB1A1

ENSBTAG00000024188_H2B

ENSBTAG00000010938_C2H1orf64

ENSBTAG00000014969_CILP2

ENSBTAG00000019425_TSPAN8

ENSBTAG00000027245

ENSBTAG00000048312

ENSBTAG00000048305

ENSBTAG00000027438

ENSBTAG00000008374_AQP2

ENSBTAG00000044009_PPP1R1C

ENSBTAG00000007144_KRT83

ENSBTAG00000047358_HIGD1C

ENSBTAG00000002139_MIXL1

ENSBTAG00000009059_PITX2

ENSBTAG00000042812_SNORD116

ENSBTAG00000011315_GJA10

ENSBTAG00000015084_MT4

ENSBTAG00000009625_DMBX1

ENSBTAG00000006468_PIWIL1

ENSBTAG00000040248

ENSBTAG00000014192_LRRTM2

ENSBTAG00000002931_BEST3

ENSBTAG00000001191_CCL17

ENSBTAG00000029827_bta-mir-125b-2

ENSBTAG00000019330_PROK2

ENSBTAG00000000534_PNOC

ENSBTAG00000011992_PTH2R

ENSBTAG00000004429_GDF5

ENSBTAG00000016573_LHCGR

ENSBTAG00000004116_PAG10

ENSBTAG00000046508

1. **Cluster 3: 121 genes.**


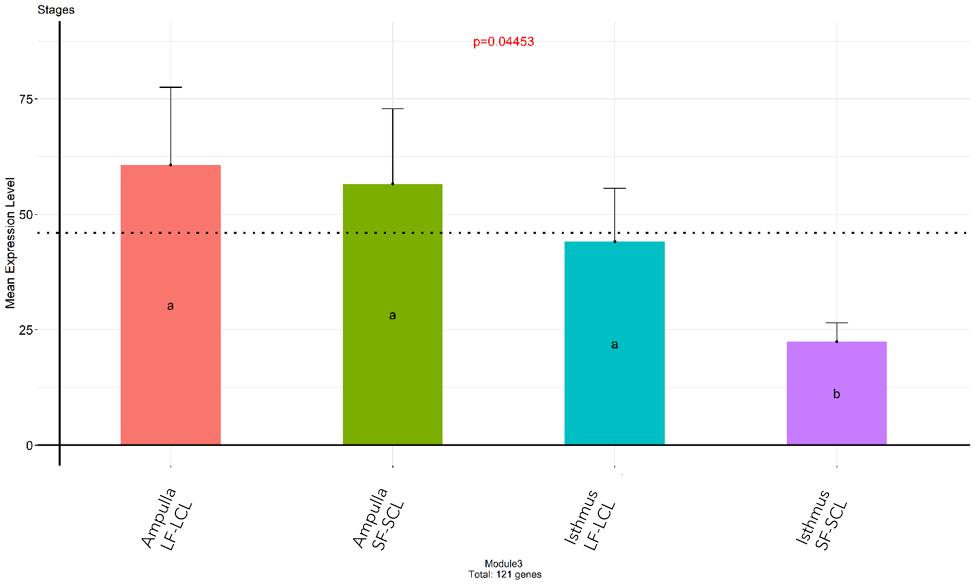


ENSBTAG00000016799_ERN1

ENSBTAG00000008176_KCNRG

ENSBTAG00000014391_CRB2

ENSBTAG00000005800_GRIA1

ENSBTAG00000001139_ACHE

ENSBTAG00000047587

ENSBTAG00000010145_SLC12A1

ENSBTAG00000038233

ENSBTAG00000017950_SLC4A8

ENSBTAG00000014016_IKZF1

ENSBTAG00000002205_CNGA1

ENSBTAG00000025219

ENSBTAG00000005978_PIK3R5

ENSBTAG00000033594_C12orf74

ENSBTAG00000014224_IGSF9B

ENSBTAG00000000783_TGFA

ENSBTAG00000024643_VEPH1

ENSBTAG00000048246

ENSBTAG00000003121_FRMPD3

ENSBTAG00000022962

ENSBTAG00000014652_TRPM8

ENSBTAG00000023192

ENSBTAG00000046611

ENSBTAG00000010392_ESRRG

ENSBTAG00000018284_KBTBD11

ENSBTAG00000037490

ENSBTAG00000000552_DISP2

ENSBTAG00000006961_NLRP13

ENSBTAG00000019600_OOEP

ENSBTAG00000001250_TFAP2A

ENSBTAG00000047979

ENSBTAG00000037951_KLHL6

ENSBTAG00000047342

ENSBTAG00000019965_TMEM255B

ENSBTAG00000038063_LIPJ

ENSBTAG00000000259_CHIA

ENSBTAG00000039196

ENSBTAG00000010345_XPNPEP2

ENSBTAG00000047528_C2CD4D

ENSBTAG00000001739_CDH20

ENSBTAG00000032125

ENSBTAG00000007200_COX6B2

ENSBTAG00000005567_BEND4

ENSBTAG00000000626_STK32A

ENSBTAG00000047383

ENSBTAG00000044011_PRTFDC1

ENSBTAG00000014674_CHRM2

ENSBTAG00000046640

ENSBTAG00000039141_TMEM74

ENSBTAG00000045701_OPRL1

ENSBTAG00000044680_bta-mir-2428

ENSBTAG00000004547_OLR1

ENSBTAG00000047866

ENSBTAG00000038903

ENSBTAG00000047765

ENSBTAG00000026194_AMZ1

ENSBTAG00000047914_MUPCDH

ENSBTAG00000043948_HCN1

ENSBTAG00000003957_PRDM7

ENSBTAG00000000960_SPATA25

ENSBTAG00000030166_BTBD17

ENSBTAG00000007001_SLC26A1

ENSBTAG00000031866

ENSBTAG00000015093_ADAMTS13

ENSBTAG00000048142_GPR45

ENSBTAG00000047351

ENSBTAG00000020537_ASIC4

ENSBTAG00000008940_NPTX1

ENSBTAG00000011818_COL26A1

ENSBTAG00000039978

ENSBTAG00000032277_KYNU

ENSBTAG00000034632_LEMD1

ENSBTAG00000044033_EDIL3

ENSBTAG00000024021

ENSBTAG00000000593_ST8SIA1

ENSBTAG00000045478_bta-mir-2308

ENSBTAG00000014555_CASS4

ENSBTAG00000039366_CD1A

ENSBTAG00000047129_B3GNT6

ENSBTAG00000017174_SCN11A

ENSBTAG00000020510_EPHA10

ENSBTAG00000000908_GPR81

ENSBTAG00000045826_C10orf105

ENSBTAG00000004714_C19orf59

ENSBTAG00000014496_CCR6

ENSBTAG00000015520_SLC11A1

ENSBTAG00000000261_FAM228A

ENSBTAG00000046817

ENSBTAG00000046420_SNORD113

ENSBTAG00000000728_ZNF597

ENSBTAG00000038711_TMEM91

ENSBTAG00000004690_CD300E

ENSBTAG00000001608

ENSBTAG00000002744_MUSK

ENSBTAG00000033316_PDCD1LG2

ENSBTAG00000019506_ODF3L1

ENSBTAG00000040550_NKX6-2

ENSBTAG00000040290_GSTO2

ENSBTAG00000007379_ALK

ENSBTAG00000012809_KIAA1045

ENSBTAG00000007890_SEC14L5

ENSBTAG00000007750

ENSBTAG00000021774_CELF5

ENSBTAG00000047839_P2RY8

ENSBTAG00000029822_bta-mir-423

ENSBTAG00000016607_TLX2

ENSBTAG00000040318

ENSBTAG00000046216

ENSBTAG00000029772_bta-let-7a-3

ENSBTAG00000036365_bta-mir-29e

ENSBTAG00000046044

ENSBTAG00000018147_NXPH2

ENSBTAG00000004148_MATN4

ENSBTAG00000012750_UPK2

ENSBTAG00000016242_DUOXA1

ENSBTAG00000046772_CLEC3A

ENSBTAG00000021657_CACNG6

ENSBTAG00000016189

ENSBTAG00000015835_PIWIL2

ENSBTAG00000043140_SNORD69

ENSBTAG00000047996

1. **Cluster 4: 103 genes.**


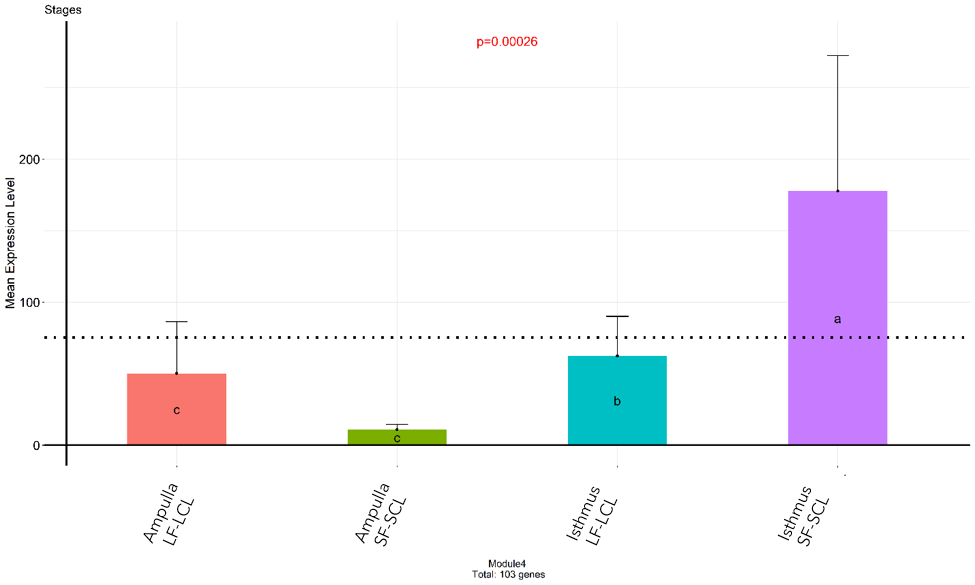


ENSBTAG00000001292_LTF

ENSBTAG00000021381_DAAM2

ENSBTAG00000022150_MXRA5

ENSBTAG00000004081_FAT3

ENSBTAG00000047648_SYT2

ENSBTAG00000011680_PAPLN

ENSBTAG00000000720_CTSL1

ENSBTAG00000006535_IRS4

ENSBTAG00000007417_GDF11

ENSBTAG00000030920

ENSBTAG00000002023_NOS1

ENSBTAG00000010464_MN1

ENSBTAG00000039789

ENSBTAG00000009862_C14orf105

ENSBTAG00000006811_EPHA3

ENSBTAG00000033529_SCN2B

ENSBTAG00000002915_GPR63

ENSBTAG00000012953_ASTN1

ENSBTAG00000024526

ENSBTAG00000019440_PROZ

ENSBTAG00000005219

ENSBTAG00000033445

ENSBTAG00000006156_BST1

ENSBTAG00000010822_PLCXD3

ENSBTAG00000009269_GRIK3

ENSBTAG00000001880_VGLL1

ENSBTAG00000019031

ENSBTAG00000018069_TNFSF15

ENSBTAG00000047459

ENSBTAG00000014488_CDH7

ENSBTAG00000012311_CACNG5

ENSBTAG00000047164

ENSBTAG00000026381_TMEM114

ENSBTAG00000015800

ENSBTAG00000030246_ENTPD8

ENSBTAG00000009911_HDC

ENSBTAG00000019132_DMP1

ENSBTAG00000012668

ENSBTAG00000018019_BCL11B

ENSBTAG00000030286_GABRA1

ENSBTAG00000004557_DHRS9

ENSBTAG00000016633_NPY2R

ENSBTAG00000022986_FAM19A5

ENSBTAG00000015128_KLK8

ENSBTAG00000014848_NR0B2

ENSBTAG00000005797_AGXT

ENSBTAG00000038171_CFHR5

ENSBTAG00000033128_CRH

ENSBTAG00000007411_F7

ENSBTAG00000046511_LYZ1

ENSBTAG00000008910_SSTR3

ENSBTAG00000000082_KCNJ14

ENSBTAG00000043911

ENSBTAG00000047191_KCNA6

ENSBTAG00000047118

ENSBTAG00000042882_SNORD113

ENSBTAG00000009349_MMEL1

ENSBTAG00000026032_ZYG11A

ENSBTAG00000003184_GATA1

ENSBTAG00000009192_SKINT1

ENSBTAG00000047155_C10orf71

ENSBTAG00000005259_UCP3

ENSBTAG00000011530_CDH15

ENSBTAG00000039186_MGC151949

ENSBTAG00000047183

ENSBTAG00000042690_SNORD113

ENSBTAG00000029844_bta-mir-125a

ENSBTAG00000014533_KEL

ENSBTAG00000036215

ENSBTAG00000039643

ENSBTAG00000047885_ACTL10

ENSBTAG00000020201_S100Z

ENSBTAG00000020096_MS4A2

ENSBTAG00000045871

ENSBTAG00000013511_VWA5B1

ENSBTAG00000029846_bta-mir-27b

ENSBTAG00000002407_TDRD5

ENSBTAG00000046341

ENSBTAG00000044191_ABCC12

ENSBTAG00000046604_GREM1

ENSBTAG00000043983_LRRC19

ENSBTAG00000013203_GP5

ENSBTAG00000045094_bta-mir-2323

ENSBTAG00000038548_SLC47A2

ENSBTAG00000043541_SNORA5

ENSBTAG00000007517_CHRNB2

ENSBTAG00000046050_SNORA73

ENSBTAG00000018955

ENSBTAG00000011120_GBX1

ENSBTAG00000037537

ENSBTAG00000029931_bta-mir-199a-1

ENSBTAG00000045209_SCARNA15

ENSBTAG00000001621_EFCAB13

ENSBTAG00000040567_MMP27

ENSBTAG00000047275

ENSBTAG00000029803_bta-mir-221

ENSBTAG00000047110

ENSBTAG00000042378_SNORA70

ENSBTAG00000021693_RPP25

ENSBTAG00000030208_FAM132B

ENSBTAG00000000008_KCNJ1

ENSBTAG00000043157_Telomerase-vert

ENSBTAG00000004886_ZAR1

1. **Cluster 5: 96 genes.**


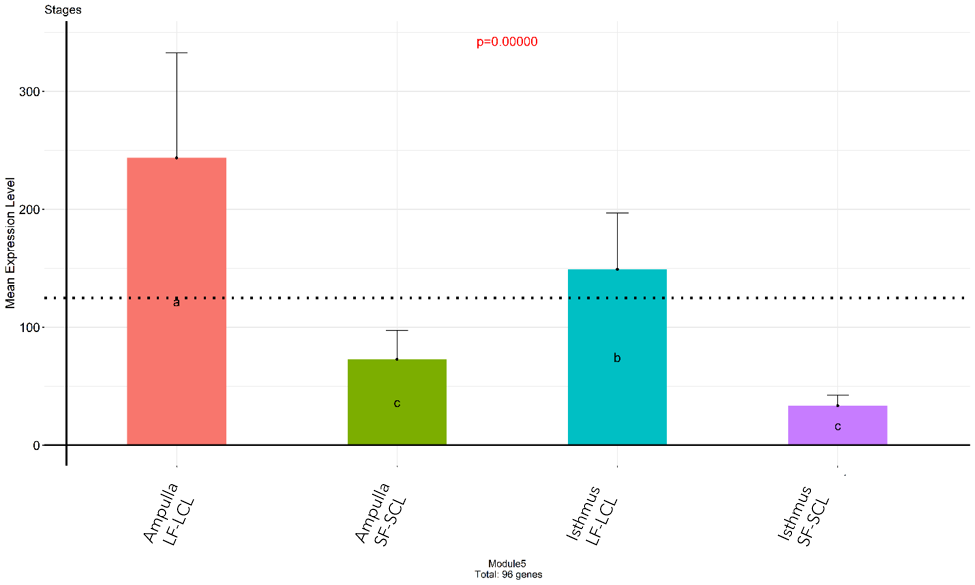


ENSBTAG00000008471_MX2

ENSBTAG00000014707_ISG15

ENSBTAG00000037527_OAS1Z

ENSBTAG00000007881_IFIT1

ENSBTAG00000004221_ESM1

ENSBTAG00000019790_MSH5

ENSBTAG00000014628_OAS2

ENSBTAG00000012406_ZBP1

ENSBTAG00000019437

ENSBTAG00000037949_GSTA5

ENSBTAG00000018235_SLC45A2

ENSBTAG00000032642

ENSBTAG00000037991

ENSBTAG00000015712_POU3F4

ENSBTAG00000002065_TFAP2C

ENSBTAG00000005091_DGKG

ENSBTAG00000019946_USP44

ENSBTAG00000013047_GRM7

ENSBTAG00000023970

ENSBTAG00000000987_OTOG

ENSBTAG00000005305_NTS

ENSBTAG00000011298_EYA1

ENSBTAG00000013569_CD38

ENSBTAG00000025812

ENSBTAG00000033759

ENSBTAG00000007650_SLC38A11

ENSBTAG00000005704_IL1RL2

ENSBTAG00000015340_ANGPTL7

ENSBTAG00000009219_CADM2

ENSBTAG00000037773_SERPINB7

ENSBTAG00000014760_FOXE1

ENSBTAG00000001975_DRP2

ENSBTAG00000005324

ENSBTAG00000014046_BPI

ENSBTAG00000004560_CLEC4F

ENSBTAG00000003684_LRFN5

ENSBTAG00000015210_LOXHD1

ENSBTAG00000010782_KIAA1210

ENSBTAG00000013422_GABRB3

ENSBTAG00000002252_CHRNA2

ENSBTAG00000010448_CPNE4

ENSBTAG00000017971_LRFN2

ENSBTAG00000010414_DMRTA2

ENSBTAG00000002123_MYO3A

ENSBTAG00000020769_OPCML

ENSBTAG00000020555_DEFB123

ENSBTAG00000000597_TMPRSS15

ENSBTAG00000014497_GPR31

ENSBTAG00000038891

ENSBTAG00000045854

ENSBTAG00000006293_PLAC1

ENSBTAG00000038215_CLCA2

ENSBTAG00000045795_KIR2DS1

ENSBTAG00000044107_FAM159A

ENSBTAG00000012079_MYLK4

ENSBTAG00000000161_TMEM40

ENSBTAG00000044830_bta-mir-339a

ENSBTAG00000002576_GLDN

ENSBTAG00000035915

ENSBTAG00000017916

ENSBTAG00000009217_WNT10A

ENSBTAG00000013766_IRGC

ENSBTAG00000021626_MLC1

ENSBTAG00000043512_SNORA68

ENSBTAG00000031612_SPINK8

ENSBTAG00000015810_PLET1

ENSBTAG00000046733

ENSBTAG00000002518_NKX6-1

ENSBTAG00000046738

ENSBTAG00000047600_ROS1

ENSBTAG00000017348_FGF5

ENSBTAG00000046815

ENSBTAG00000031309_RAG2

ENSBTAG00000042744_SNORA71

ENSBTAG00000004569_GLIPR1L1

ENSBTAG00000008026_OXT

ENSBTAG00000047411

ENSBTAG00000038680

ENSBTAG00000006943_EFCAB3

ENSBTAG00000011616_MPO

ENSBTAG00000005895

ENSBTAG00000001075_CALB2

ENSBTAG00000042559_SNORA42

ENSBTAG00000008922_APOL6

ENSBTAG00000013906

ENSBTAG00000008463_SLC22A14

ENSBTAG00000039674

ENSBTAG00000006648

ENSBTAG00000047982

ENSBTAG00000045760

ENSBTAG00000008295_ACTL6B

ENSBTAG00000037917_SLC17A1

ENSBTAG00000046052_ANKRD63

ENSBTAG00000007687_GDF6

ENSBTAG00000030472_TAS2R42

ENSBTAG00000047072

1. **Cluster 6: 54 genes.**


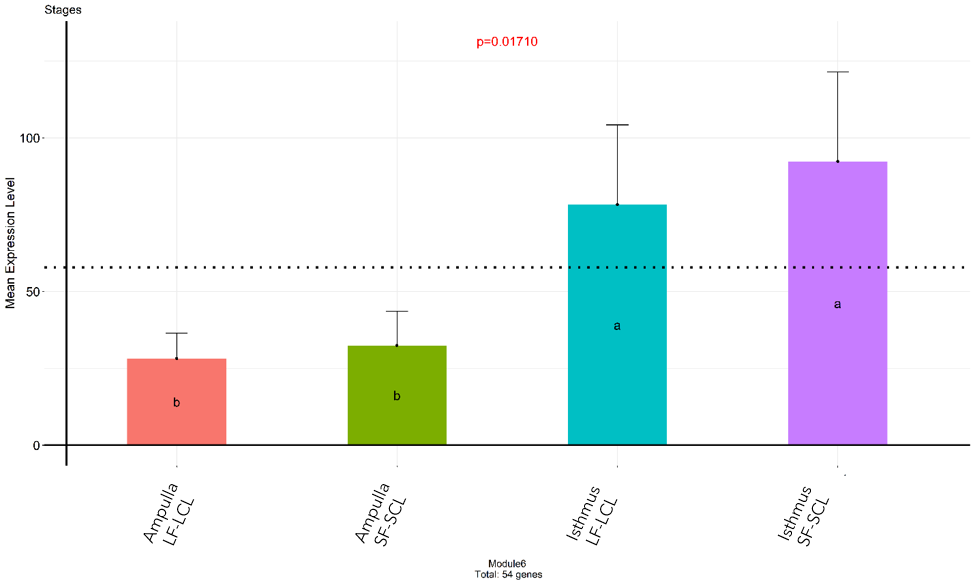


ENSBTAG00000021565_PRSS2

ENSBTAG00000003658_RELN

ENSBTAG00000010285_MMRN1

ENSBTAG00000007268_F13A1

ENSBTAG00000016520_TPSB1

ENSBTAG00000022514_CD5L

ENSBTAG00000026836_ABI3BP

ENSBTAG00000019669_CD163

ENSBTAG00000012185_CLEC4E

ENSBTAG00000015415_UNC80

ENSBTAG00000015008_CPA3

ENSBTAG00000020481_GXYLT2

ENSBTAG00000026119_PTI

ENSBTAG00000002680_TMEM132E

ENSBTAG00000022715_DMBT1

ENSBTAG00000003392_GABRA5

ENSBTAG00000005419_ABP1

ENSBTAG00000017948_SPIN4

ENSBTAG00000048155_FCNB

ENSBTAG00000048097

ENSBTAG00000022498

ENSBTAG00000017073_HPGDS

ENSBTAG00000048189

ENSBTAG00000003432_ZAN

ENSBTAG00000012034_KRT4

ENSBTAG00000001893_TM4SF4

ENSBTAG00000043081_SNORD98

ENSBTAG00000013849_RGS13

ENSBTAG00000043048_SNORD59

ENSBTAG00000020499_PLA2G2D3

ENSBTAG00000047454_OLAH

ENSBTAG00000016234_DUOX2

ENSBTAG00000001804_GPR179

ENSBTAG00000046662_TRPM5

ENSBTAG00000029925_bta-mir-125b-1

ENSBTAG00000001082_SH2D5

ENSBTAG00000014062_FRMD1

ENSBTAG00000018792_SLC22A11

ENSBTAG00000043342_SNORD113

ENSBTAG00000038422

ENSBTAG00000008851

ENSBTAG00000045184_SNORD127

ENSBTAG00000045583

ENSBTAG00000015763_SPATA22

ENSBTAG00000010858_ABCC11

ENSBTAG00000019007

ENSBTAG00000045376_SNORD116

ENSBTAG00000046252

ENSBTAG00000047889

ENSBTAG00000038576_GBP6

ENSBTAG00000010127_MIP

ENSBTAG00000017743_XIRP2

ENSBTAG00000047001

ENSBTAG00000046308

1. **Cluster 7: 52 genes.**


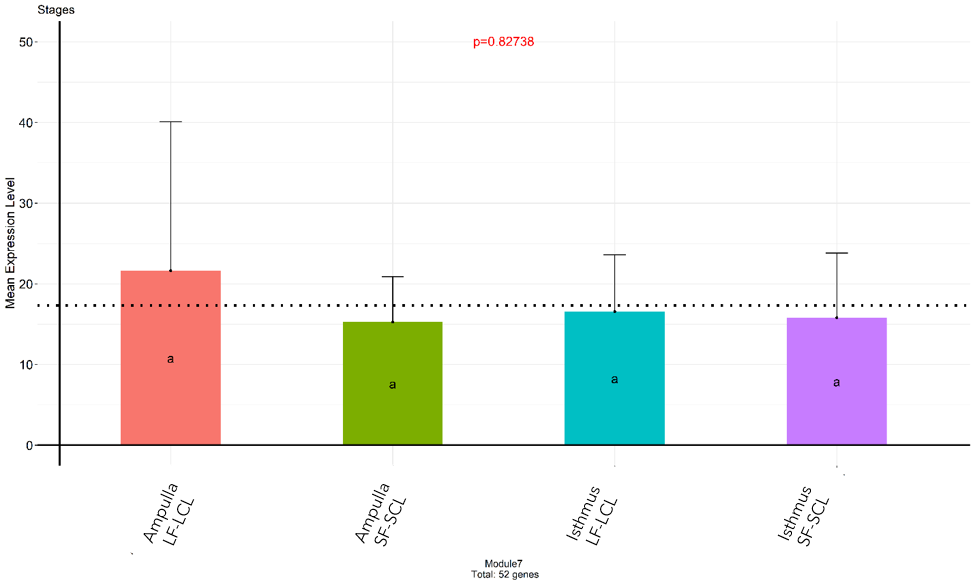


ENSBTAG00000005714_ACTC1

ENSBTAG00000023393_AIRE

ENSBTAG00000014328

ENSBTAG00000020285_PRG3

ENSBTAG00000047764

ENSBTAG00000020991_COL19A1

ENSBTAG00000006239

ENSBTAG00000016210_LYPD2

ENSBTAG00000003020_SRCRB4D

ENSBTAG00000020887_CAV2.3

ENSBTAG00000005667_SPATA31D1

ENSBTAG00000008017_UNC79

ENSBTAG00000047336

ENSBTAG00000045788

ENSBTAG00000027328

ENSBTAG00000047767_MUC3A

ENSBTAG00000033837

ENSBTAG00000009752_CD70

ENSBTAG00000021328_SPHKAP

ENSBTAG00000001562_NFE2

ENSBTAG00000031756

ENSBTAG00000016148

ENSBTAG00000046430_ZNF804B

ENSBTAG00000000401_TTC24

ENSBTAG00000047363

ENSBTAG00000047181

ENSBTAG00000013763_C10orf82

ENSBTAG00000038262

ENSBTAG00000010349_IL1A

ENSBTAG00000046035

ENSBTAG00000046709

ENSBTAG00000016159_NPBWR1

ENSBTAG00000017343_COLEC10

ENSBTAG00000016327_SLC28A2

ENSBTAG00000038159

ENSBTAG00000046726_CLDN20

ENSBTAG00000047270

ENSBTAG00000046298_SYCE3

ENSBTAG00000043233_SNORA27

ENSBTAG00000002983_NT5C1A

ENSBTAG00000039673

ENSBTAG00000043531_SNORD18

ENSBTAG00000047596_C2orf53

ENSBTAG00000046442

ENSBTAG00000029889_bta-mir-452

ENSBTAG00000001315_DMRTA1

ENSBTAG00000009409_PANX3

ENSBTAG00000003442_LRIT1

ENSBTAG00000013095_FOXN1

ENSBTAG00000037554

ENSBTAG00000040261

ENSBTAG00000020007

1. **Cluster 8: 51 genes.**


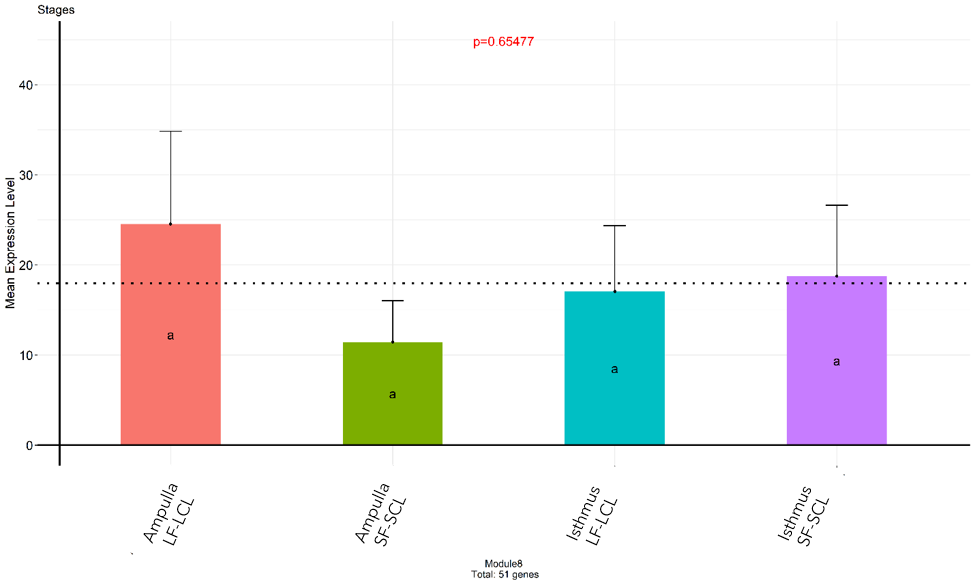


ENSBTAG00000045929

ENSBTAG00000031017

ENSBTAG00000037989

ENSBTAG00000038604_H2B

ENSBTAG00000001308

ENSBTAG00000014761_PGA

ENSBTAG00000031359

ENSBTAG00000017969_CA4

ENSBTAG00000002664_WNT9B

ENSBTAG00000006745_FGG

ENSBTAG00000003775_C8B

ENSBTAG00000004508_DBH

ENSBTAG00000024980_VIT

ENSBTAG00000037534_KLRF1

ENSBTAG00000048184

ENSBTAG00000031738

ENSBTAG00000017896_ST8SIA2

ENSBTAG00000040026

ENSBTAG00000001730_ODF3

ENSBTAG00000000595_FBN3

ENSBTAG00000000381_S1PR4

ENSBTAG00000039932

ENSBTAG00000021735_GALNT14

ENSBTAG00000001443

ENSBTAG00000010151_CA5A

ENSBTAG00000044037_DACH2

ENSBTAG00000039065_HS6ST3

ENSBTAG00000034064_MGC133764

ENSBTAG00000044536_SNORA30

ENSBTAG00000005560_ST18

ENSBTAG00000045309_bta-mir-2443

ENSBTAG00000031186_GBP6

ENSBTAG00000007306_RAB3C

ENSBTAG00000039052

ENSBTAG00000015125_KLK5

ENSBTAG00000040150

ENSBTAG00000010897_RESP18

ENSBTAG00000013014_GPR61

ENSBTAG00000043483_SNORD83

ENSBTAG00000017434_MROH2A

ENSBTAG00000033352_DMRT1

ENSBTAG00000047473

ENSBTAG00000013841_C21orf140

ENSBTAG00000012826_SLC25A31

ENSBTAG00000040222

ENSBTAG00000032873

ENSBTAG00000037844_CDH18

ENSBTAG00000012780_LPO

ENSBTAG00000008426

ENSBTAG00000034939_BTNL2

ENSBTAG00000047170

1. **Cluster 9: 43 genes.**


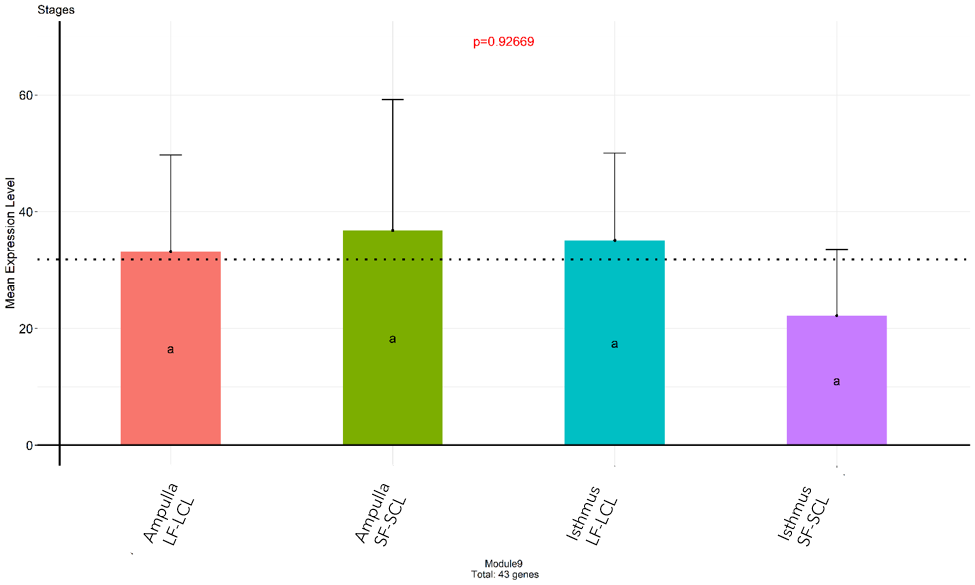


ENSBTAG00000026502

ENSBTAG00000020247_ADCYAP1R1

ENSBTAG00000024272_BT.37579

ENSBTAG00000048304

ENSBTAG00000025903_GLDC

ENSBTAG00000039563

ENSBTAG00000031802_SPATA16

ENSBTAG00000001260_PINLYP

ENSBTAG00000039329_RAET1G

ENSBTAG00000045905_PCDH15

ENSBTAG00000014847_BBOX1

ENSBTAG00000044183_SPINK7

ENSBTAG00000040580

ENSBTAG00000039827

ENSBTAG00000018585_GABRB2

ENSBTAG00000039994

ENSBTAG00000039849

ENSBTAG00000038797

ENSBTAG00000034302

ENSBTAG00000006769_HSD3B

ENSBTAG00000021340_POU2F3

ENSBTAG00000011921_TIGIT

ENSBTAG00000045782_BMP15

ENSBTAG00000009273

ENSBTAG00000045706

ENSBTAG00000045768

ENSBTAG00000003078_COL10A1

ENSBTAG00000017227

ENSBTAG00000017062_ADM2

ENSBTAG00000031232

ENSBTAG00000005548_HYAL4

ENSBTAG00000010062_SLC26A4

ENSBTAG00000046313_CCDC54

ENSBTAG00000025664

ENSBTAG00000005702_CNGB1

ENSBTAG00000046494_CORT

ENSBTAG00000019468_ACTL7A

ENSBTAG00000020351_SLC34A1

ENSBTAG00000000273_IL5RA

ENSBTAG00000033377

ENSBTAG00000004581_MS4A14

ENSBTAG00000047398

ENSBTAG00000047924_MAB21L2

1. **Cluster 10: 42 genes.**


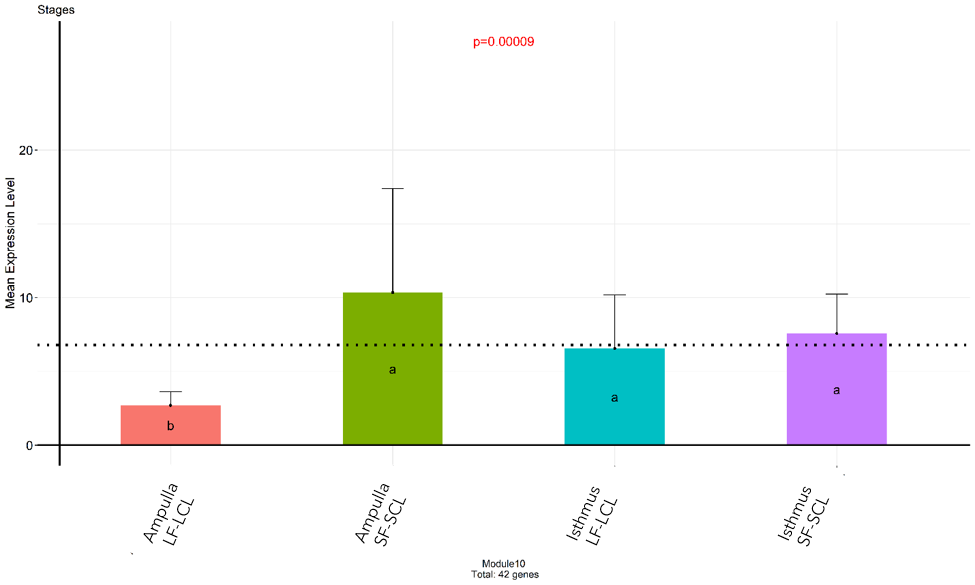


ENSBTAG00000019529_ATP8A2

ENSBTAG00000009775_PRRG3

ENSBTAG00000007424_LIF

ENSBTAG00000047675_SIGLEC6

ENSBTAG00000011990_ALOX12

ENSBTAG00000016768_SCN3B

ENSBTAG00000040000

ENSBTAG00000026994_C2H2orf88

ENSBTAG00000010352_KCNK16

ENSBTAG00000012759_FBXO41

ENSBTAG00000046213_SNORD116

ENSBTAG00000011940_ZNF831

ENSBTAG00000006566_GPR43

ENSBTAG00000006691_GABRA3

ENSBTAG00000039347_CLEC4D

ENSBTAG00000026626_ANHX

ENSBTAG00000016794_SLC10A6

ENSBTAG00000045197_SNORD125

ENSBTAG00000015175

ENSBTAG00000012347_SLC51A

ENSBTAG00000020859_SERPINF2

ENSBTAG00000042931_SNORD75

ENSBTAG00000034220_CDKN2A

ENSBTAG00000032429

ENSBTAG00000016151_VTN

ENSBTAG00000042456_SNORD43

ENSBTAG00000015525_SLC39A12

ENSBTAG00000003387_PIP5KL1

ENSBTAG00000025775_INSL3

ENSBTAG00000038224_GDF7

ENSBTAG00000038955

ENSBTAG00000014400_E2F2

ENSBTAG00000016880_TAS2R3

ENSBTAG00000043165_SNORA70

ENSBTAG00000016365_ABCG5

ENSBTAG00000016835_IL17F

ENSBTAG00000005952_CEBPE

ENSBTAG00000047971_KIR3DL1

ENSBTAG00000006635_DBX2

ENSBTAG00000018787_GCM2

ENSBTAG00000042370_SNORA56

ENSBTAG00000032236_CA1

1. **Cluster 11: 32 genes.**


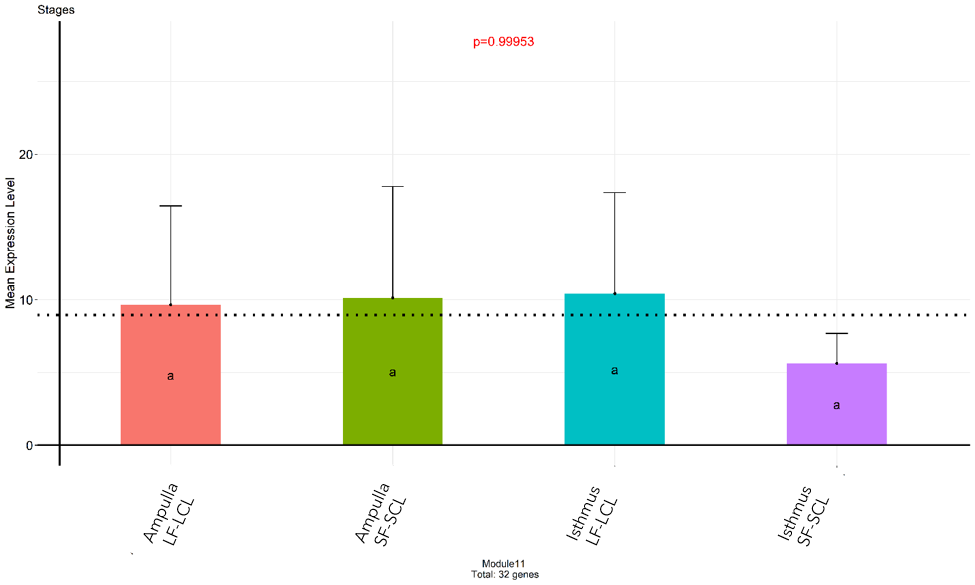


ENSBTAG00000009739_CSMD1

ENSBTAG00000015412_TGM7

ENSBTAG00000004595_GML

ENSBTAG00000019931

ENSBTAG00000000606_SYT10

ENSBTAG00000002253_FKBP6

ENSBTAG00000039257

ENSBTAG00000027033

ENSBTAG00000046345

ENSBTAG00000017648_TIFAB

ENSBTAG00000040146

ENSBTAG00000039929_RXFP3

ENSBTAG00000033359

ENSBTAG00000015061

ENSBTAG00000013106_C19orf81

ENSBTAG00000042499_SNORA61

ENSBTAG00000047175_GP6

ENSBTAG00000031052_CDK5R2

ENSBTAG00000019172_IGSF21

ENSBTAG00000006121_SBK2

ENSBTAG00000002071

ENSBTAG00000046294

ENSBTAG00000003479_MATN1

ENSBTAG00000009444_SLC15A5

ENSBTAG00000034577

ENSBTAG00000018162_VN1R4

ENSBTAG00000031763

ENSBTAG00000040133_C3orf20

ENSBTAG00000043795_SNORD31

ENSBTAG00000020669_PROKR1

ENSBTAG00000001271_PLG

ENSBTAG00000046068

1. **Cluster 12: 20 genes.**


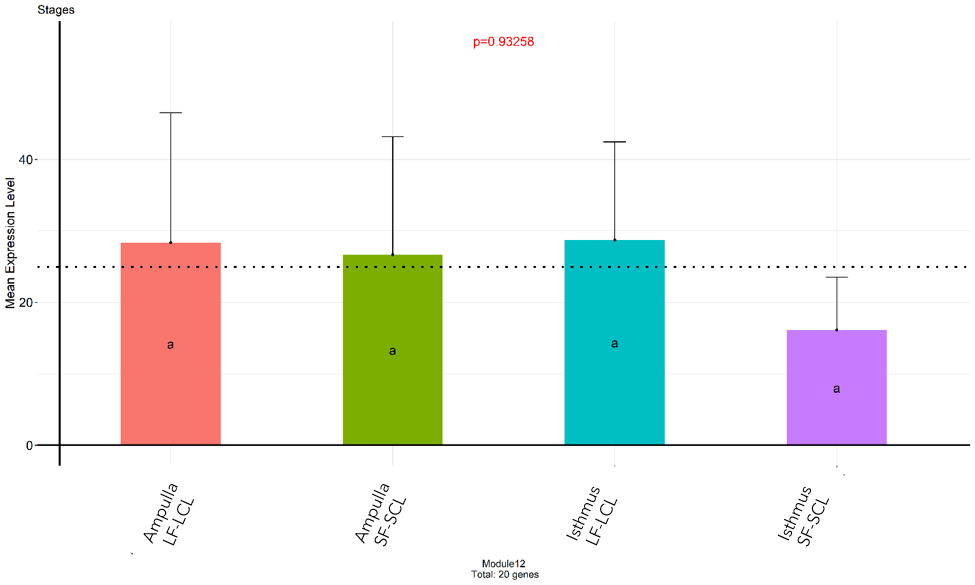


ENSBTAG00000038112_ULBP3

ENSBTAG00000017780_DPYSL4

ENSBTAG00000010033

ENSBTAG00000040409

ENSBTAG00000045845

ENSBTAG00000006539_SIRPB2

ENSBTAG00000014141_SPATA4

ENSBTAG00000038698

ENSBTAG00000032206_HEMGN

ENSBTAG00000033708

ENSBTAG00000046683

ENSBTAG00000022379_VSIG2

ENSBTAG00000046431

ENSBTAG00000006582_SSTR1

ENSBTAG00000014523_DIRAS2

ENSBTAG00000034320

ENSBTAG00000043979

ENSBTAG00000010676_RBM44

ENSBTAG00000006451_GAP43

ENSBTAG00000007633_ANKRD34B

1. **Complete list of not correlated genes.**

ENSBTAG00000047946

ENSBTAG00000021516_GSTA1

ENSBTAG00000010413_TMEM169

ENSBTAG00000019870_SLC14A1

ENSBTAG00000011985

ENSBTAG00000030543_SLC6A16

ENSBTAG00000017157_CCDC110

ENSBTAG00000018543_VIL1

ENSBTAG00000020361_SLC35F3

ENSBTAG00000002009

ENSBTAG00000017251_SLC26A8

ENSBTAG00000000951_JAKMIP3

ENSBTAG00000039194_C3H3orf22

ENSBTAG00000038275_CYP27C1

ENSBTAG00000025718_HMGCLL1

ENSBTAG00000020426_TFAP2B

ENSBTAG00000015132_RXFP2

ENSBTAG00000022209_CABP2

ENSBTAG00000047019

ENSBTAG00000046098_ACSM4

ENSBTAG00000009585_CDH16

ENSBTAG00000045896_NPTX2

ENSBTAG00000043234_SNORD87

ENSBTAG00000005164_GZMK

ENSBTAG00000018576_DPYSL5

ENSBTAG00000000751_RRH

ENSBTAG00000042677_SNORD59

ENSBTAG00000024901

ENSBTAG00000021326_CCL20

ENSBTAG00000013378_AADACL2

ENSBTAG00000007720_NKG2A

ENSBTAG00000002773

ENSBTAG00000030119

ENSBTAG00000038565

ENSBTAG00000003977_SLC52A3

ENSBTAG00000019690_PNLIPRP3

ENSBTAG00000042440_SNORA9

ENSBTAG00000021368

ENSBTAG00000045514

ENSBTAG00000006649_TSSK6

ENSBTAG00000032761_CALHM3

ENSBTAG00000010632_FABP6

ENSBTAG00000038433

ENSBTAG00000043383_SNORD24

ENSBTAG00000013974_TMEM215

ENSBTAG00000042100_SNORD102

ENSBTAG00000023079

ENSBTAG00000046478

ENSBTAG00000015715_SPOCD1

ENSBTAG00000031346_ANKUB1

ENSBTAG00000000776

ENSBTAG00000026157

ENSBTAG00000039401_TRB@

ENSBTAG00000046973

ENSBTAG00000015045_PRSS21

ENSBTAG00000000237

ENSBTAG00000015592_GPR84

ENSBTAG00000004474_BANF2

ENSBTAG00000014869

ENSBTAG00000048175

ENSBTAG00000019770_APOA4

ENSBTAG00000002595_ABCB5

ENSBTAG00000047937

ENSBTAG00000042819_snoZ39

ENSBTAG00000005891_FCRL1

ENSBTAG00000045878

ENSBTAG00000009840_OR2AE1

ENSBTAG00000045951

ENSBTAG00000047992_C17orf107

ENSBTAG00000040188_OR12D2

ENSBTAG00000000644_S100A5

ENSBTAG00000000029_LYZL6

ENSBTAG00000046466_SNORA15

ENSBTAG00000006438_ACTL8

ENSBTAG00000020022_IL13RA2

ENSBTAG00000021882

ENSBTAG00000017357_CLCA4

ENSBTAG00000017718_CCL22

ENSBTAG00000043914_SNORD19

ENSBTAG00000042644_snoU6-53

ENSBTAG00000019469_SPACA1

ENSBTAG00000046693

ENSBTAG00000038568

ENSBTAG00000018204_MYH1

ENSBTAG00000016366_ABCG8

ENSBTAG00000039086

ENSBTAG00000046169_CPA1

ENSBTAG00000006924_NXPH1

ENSBTAG00000012478_ALOXE3

ENSBTAG00000008876_EFCAB9

ENSBTAG00000030454

ENSBTAG00000015047

ENSBTAG00000001310_RHO

ENSBTAG00000036102

ENSBTAG00000031845_SSX5

ENSBTAG00000044025_IL1RAPL2

ENSBTAG00000038928

ENSBTAG00000014514

ENSBTAG00000012052_PADI4

ENSBTAG00000004218_SYNDIG1L

ENSBTAG00000001231_CDHR1

ENSBTAG00000047526_GSC2

ENSBTAG00000047423_C19orf69

ENSBTAG00000014284_ALPK2

ENSBTAG00000042579_snoU6-53

ENSBTAG00000018917_EN2

ENSBTAG00000043528_SNORA46

ENSBTAG00000024318

ENSBTAG00000019435

ENSBTAG00000045672

ENSBTAG00000029892_bta-mir-10a

ENSBTAG00000018880

ENSBTAG00000031236

ENSBTAG00000048101_SNORD113

ENSBTAG00000015518_KRT12

ENSBTAG00000047396

ENSBTAG00000011808_MSTN

ENSBTAG00000046663_CBY3

ENSBTAG00000014682_AANAT

ENSBTAG00000023648

ENSBTAG00000046591

ENSBTAG00000042366_SNORA22
