# Supplementary material for: Steroidal Regulation of Oviductal microRNAs Is Associated with microRNA-Processing in Beef Cows
Source: Int J Mol Sci. 2021 Jan 19;22(2):953. doi: 10.3390/ijms22020953 (PMC7835783; doi:10.3390/ijms22020953)
Supplement: Supplementary file 1 [file ijms-22-00953-s001.zip › Supplementary Material 3.docx]

**Supplementary Material 3. Clusters of miRNAs that presented a specific expression pattern in each region (ampulla or isthmus) and group (LF-LCL or SF-SCL).** CEMITOOL package was used to identify clusters of miRNAs with similar expression. Eight clusters were identified and the representative figures, as well as the list of miRNAs are presented next. Note that the scale of the Y-axis varies according to cluster.

1. **Cluster 1: 25 miRNAs.**


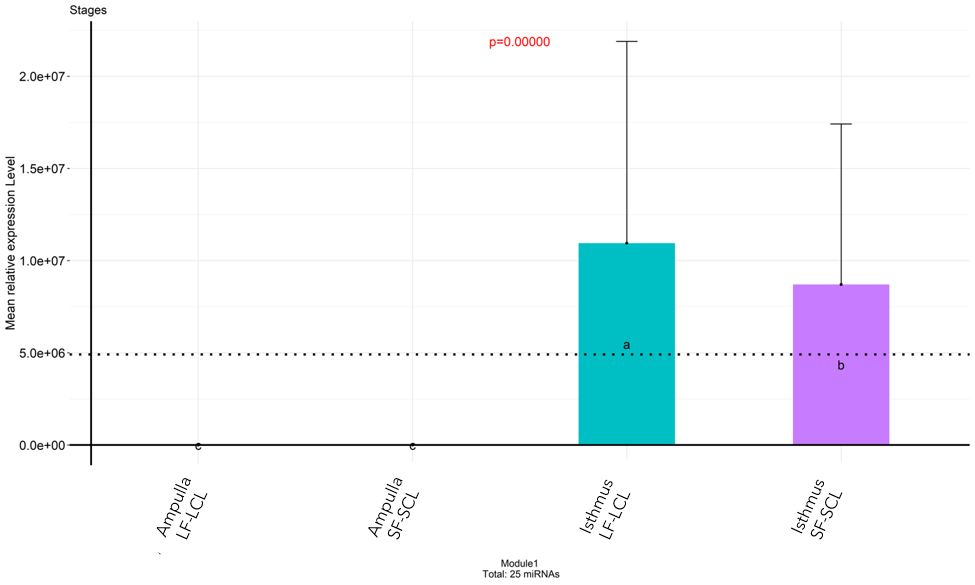


bta-miR-106a

bta-miR-122

bta-miR-124a

bta-miR-124b

bta-miR-145

bta-miR-152

bta-miR-185

bta-miR-26a

Hm/Ms/Rt T1 snRNA

bta-miR-345-5p

bta-miR-346

bta-miR-378c

bta-miR-425-3p

bta-miR-431

bta-miR-449d

bta-miR-483

bta-miR-488

bta-miR-502b

bta-miR-504

bta-miR-584

bta-miR-652

bta-miR-669

bta-miR-671

bta-miR-761

bta-miR-885

1. **Cluster 2: 17 miRNAs.**


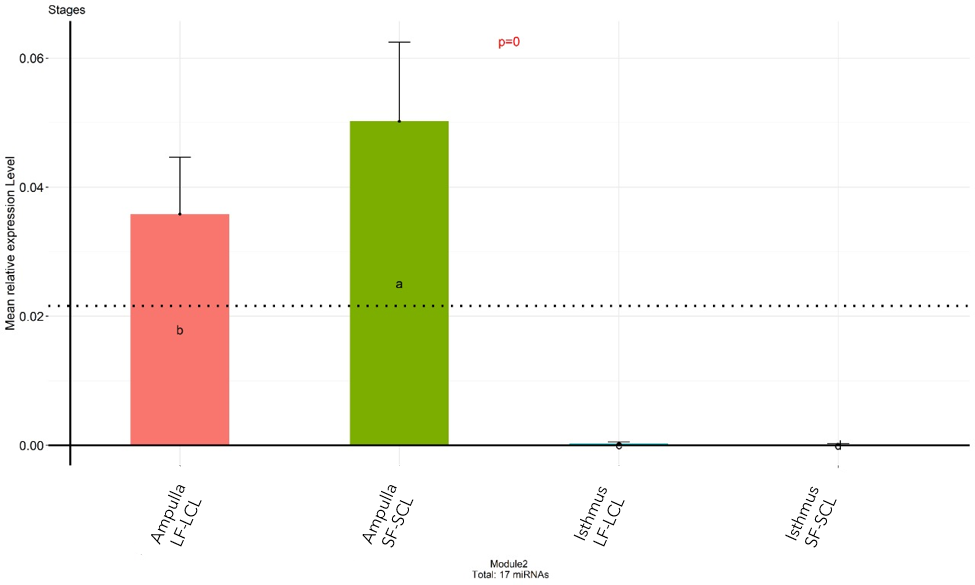


bta-miR-100

bta-miR-101

bta-miR-103

bta-miR-10a

bta-miR-10b

bta-miR-125a

bta-miR-126-3p

bta-miR-126-5p

bta-miR-135a

bta-miR-148a

bta-miR-16a

bta-miR-195

bta-miR-199a-5p

bta-miR-199b

bta-miR-29d-3p

bta-miR-424-5p

bta-miR-99a-5p

1. **Cluster 3: 15 miRNAs.**


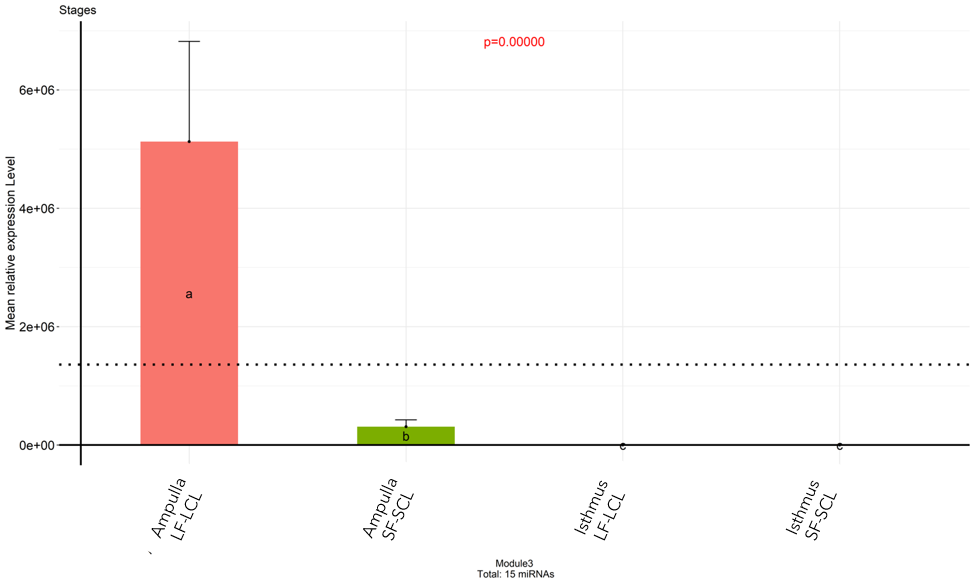


bta-let-7b

bta-miR-149-3p

bta-miR-181d

bta-miR-186

bta-miR-19a

bta-miR-20a

bta-miR-210

bta-miR-221

bta-miR-296-5p

bta-miR-30a-5p

bta-miR-320a

bta-miR-365-5p

bta-miR-494

bta-miR-92a

bta-miR-940

1. **Cluster 4: 14 miRNAs.**


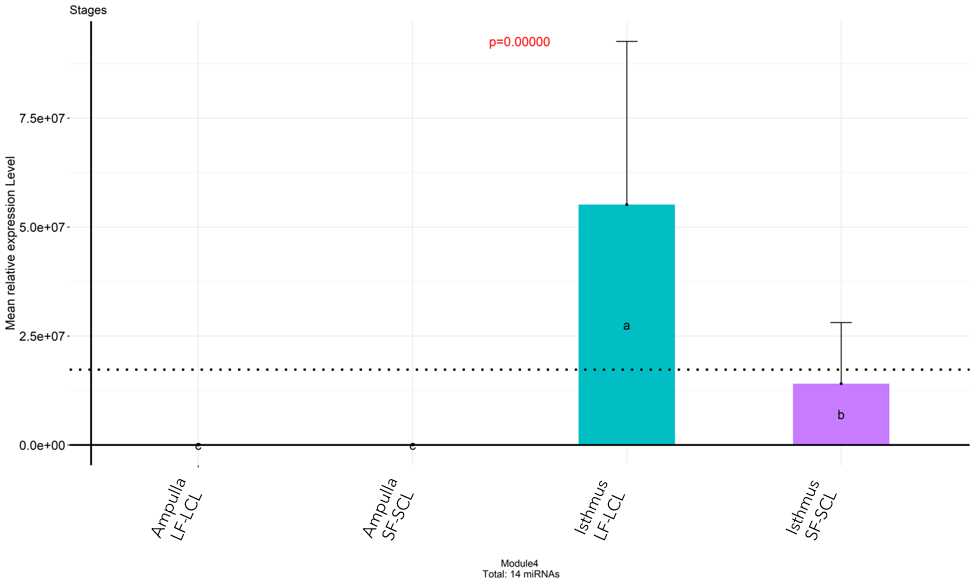


bta-miR-19b

bta-miR-17-3p

bta-miR-183

bta-miR-192

bta-miR-193b

bta-miR-216a

bta-miR-219-3p

bta-miR-223

bta-miR-23b-5p

bta-miR-28

RNT43 snoRNA

bta-miR-495

bta-miR-654

bta-miR-769

1. **Cluster 5: 13 miRNAs.**


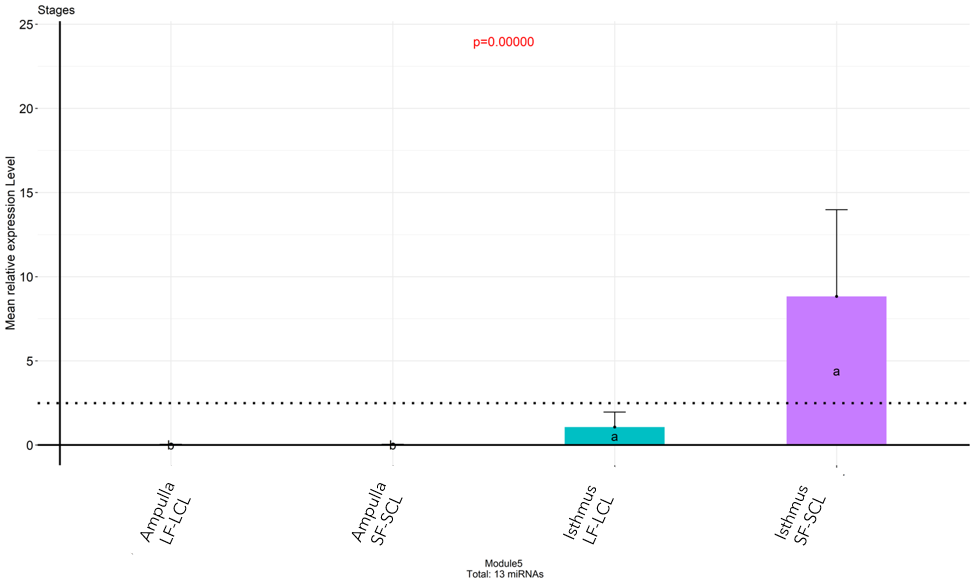


bta-miR-143

bta-miR-193a-5p

bta-miR-199a-3p

bta-miR-200b

bta-miR-132

bta-miR-138

bta-miR-181b

bta-miR-154b

bta-miR-17-5p

bta-miR-196b

bta-miR-211

bta-miR-219

bta-miR-99b

1. **Cluster 6: 13 miRNAs.**


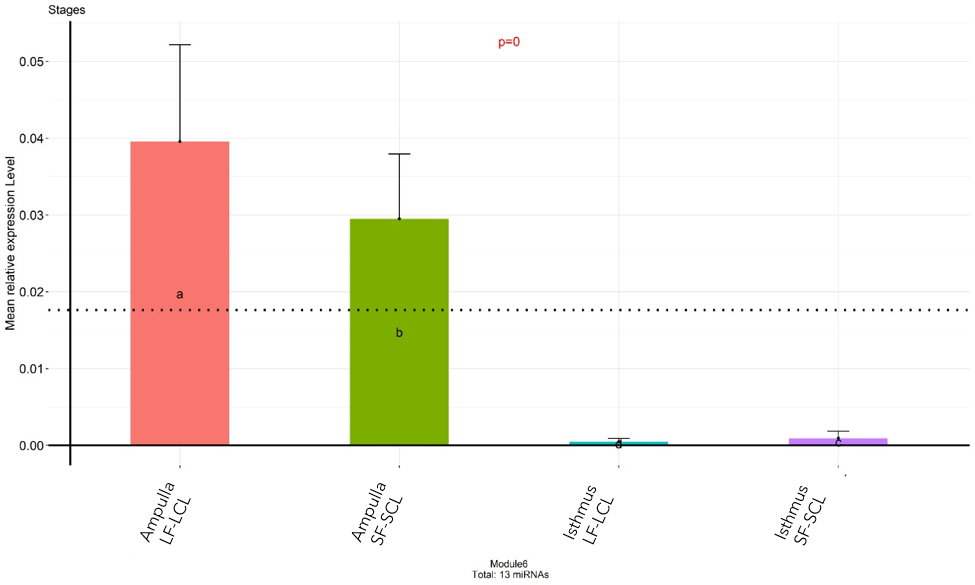


bta-let-7a-5p

bta-let-7c

bta-let-7d

bta-let-7e

bta-let-7f

bta-let-7g

bta-let-7i

bta-miR-15a

bta-miR-16b

bta-miR-200a

bta-miR-34b

bta-miR-34c

bta-miR-449a

1. **Cluster 7: 12 miRNAs.**


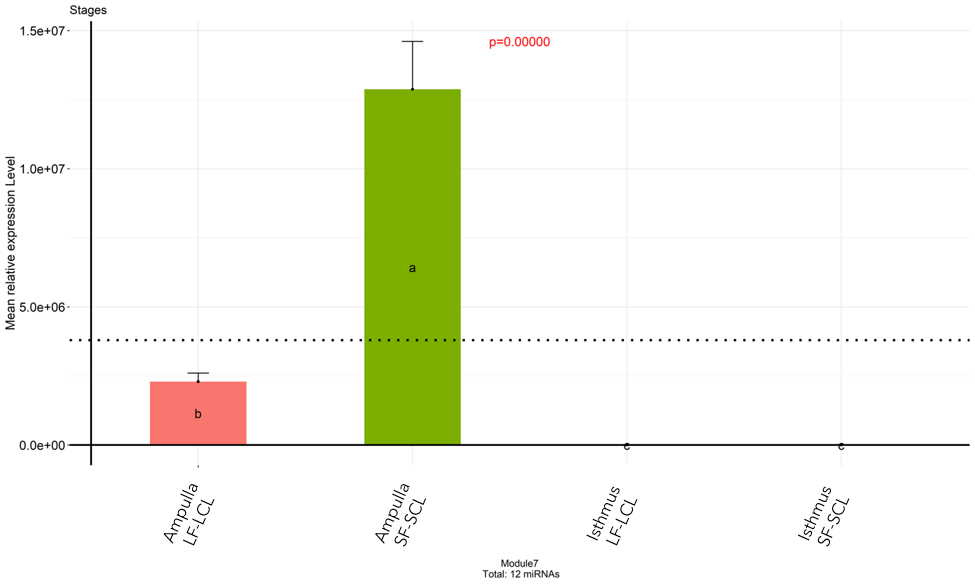


bta-miR-106b

bta-miR-148b

bta-miR-23a

bta-miR-29b

bta-miR-30c

bta-miR-339b

bta-miR-375

bta-miR-378

bta-miR-631

bta-miR-664b

bta-miR-92b

bta-miR-93

1. **Cluster 8: 12 miRNAs.**


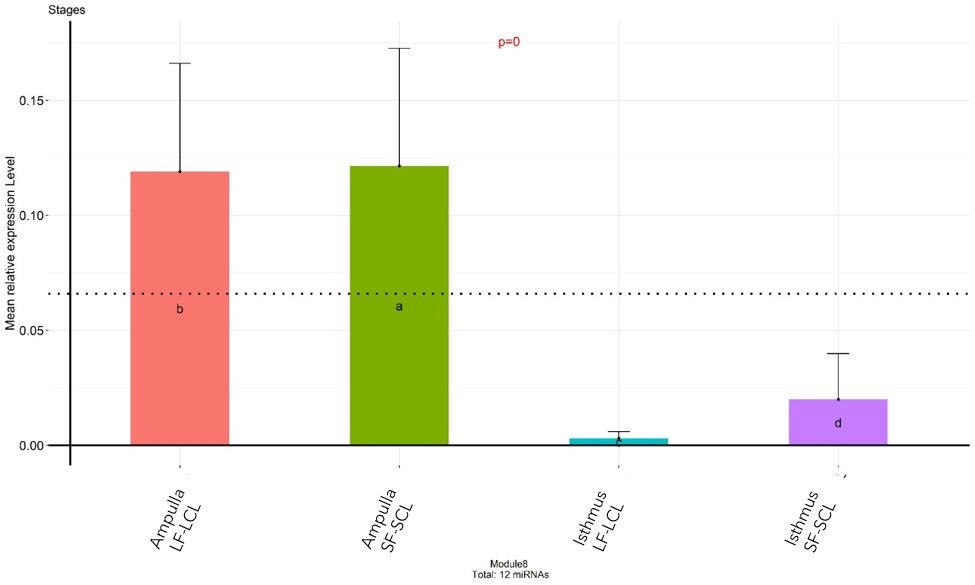


bta-miR-125b

bta-miR-130b

bta-miR-151-3p

bta-miR-151-5p

bta-miR-199c

bta-miR-20b

bta-miR-25

bta-miR-27b

bta-miR-29d-5p

bta-miR-30e-5p

bta-miR-31

bta-miR-677

1. **Complete list of non-clustered miRNAs.**

bta-miR-191

bta-miR-200c

bta-miR-222

bta-miR-24-3p

bta-miR-29a

bta-miR-29c

bta-miR-30b-5p

bta-miR-30d

bta-miR-323

bta-miR-34a

bta-miR-423-5p

bta-miR-665

bta-miR-874

bta-miR-187

bta-miR-188

bta-miR-30f

bta-miR-328

bta-miR-329b

bta-miR-342

bta-miR-383

bta-miR-409b

bta-miR-429

bta-miR-432

bta-miR-449c

bta-miR-490

bta-miR-491

bta-miR-493

bta-miR-503-5p

bta-miR-532

bta-miR-544a

bta-miR-658

bta-miR-660

bta-miR-873
