# Supplementary material for: Steroidal Regulation of Oviductal microRNAs Is Associated with microRNA-Processing in Beef Cows
Source: Int J Mol Sci. 2021 Jan 19;22(2):953. doi: 10.3390/ijms22020953 (PMC7835783; doi:10.3390/ijms22020953)
Supplement: Supplementary file 1 [file ijms-22-00953-s001.zip › Supplementary Material 4.docx]

**Supplementary Material 4.** miRNA/mRNA correlation. Only significative correlations were included. miRNA appears in alphabetic order

1. **bta-let-7b.**


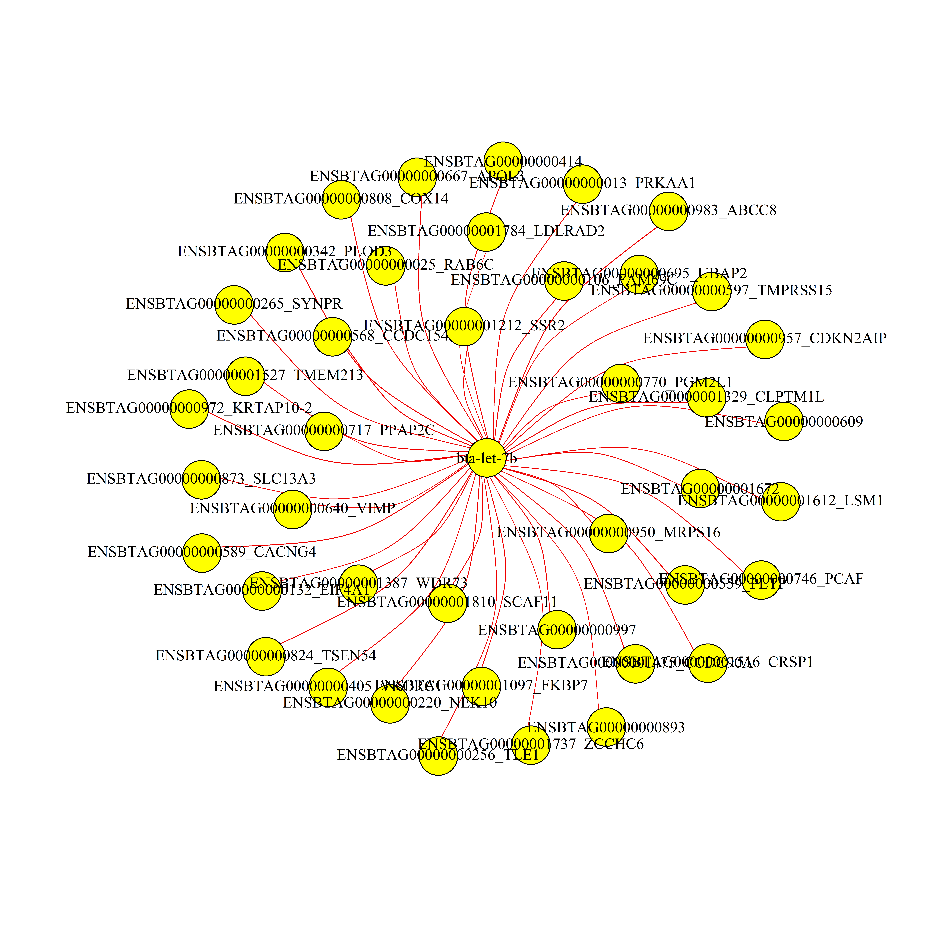


| **Correlation** | ***P* value** | **Correlated** | **With** |
| --- | --- | --- | --- |
| -0.36 | 0.029 | bta-let-7b | ENSBTAG00000000013_PRKAA1 |
| -0.38 | 0.0206 | bta-let-7b | ENSBTAG00000000025_RAB6C |
| -0.34 | 0.04 | bta-let-7b | ENSBTAG00000000106_FAM69C |
| -0.36 | 0.029 | bta-let-7b | ENSBTAG00000000132_EIF4A1 |
| -0.35 | 0.0341 | bta-let-7b | ENSBTAG00000000220_NEK10 |
| -0.37 | 0.0245 | bta-let-7b | ENSBTAG00000000256_TLE1 |
| -0.36 | 0.029 | bta-let-7b | ENSBTAG00000000265_SYNPR |
| -0.33 | 0.0468 | bta-let-7b | ENSBTAG00000000342_PLOD3 |
| -0.38 | 0.0206 | bta-let-7b | ENSBTAG00000000405_VKORC1 |
| -0.35 | 0.0341 | bta-let-7b | ENSBTAG00000000414 |
| -0.33 | 0.0468 | bta-let-7b | ENSBTAG00000000559_PLTP |
| -0.33 | 0.0468 | bta-let-7b | ENSBTAG00000000568_CCDC154 |
| -0.33 | 0.0468 | bta-let-7b | ENSBTAG00000000589_CACNG4 |
| -0.38 | 0.0206 | bta-let-7b | ENSBTAG00000000597_TMPRSS15 |
| -0.38 | 0.0206 | bta-let-7b | ENSBTAG00000000609 |
| -0.4 | 0.0145 | bta-let-7b | ENSBTAG00000000640_VIMP |
| -0.35 | 0.0341 | bta-let-7b | ENSBTAG00000000667_APOL3 |
| -0.4 | 0.0145 | bta-let-7b | ENSBTAG00000000695_UBAP2 |
| -0.38 | 0.0206 | bta-let-7b | ENSBTAG00000000717_PPAP2C |
| -0.38 | 0.0206 | bta-let-7b | ENSBTAG00000000746_PCAF |
| -0.36 | 0.029 | bta-let-7b | ENSBTAG00000000770_PGM2L1 |
| -0.34 | 0.04 | bta-let-7b | ENSBTAG00000000808_COX14 |
| -0.33 | 0.0468 | bta-let-7b | ENSBTAG00000000824_TSEN54 |
| -0.36 | 0.029 | bta-let-7b | ENSBTAG00000000873_SLC13A3 |
| -0.39 | 0.0173 | bta-let-7b | ENSBTAG00000000893 |
| -0.36 | 0.029 | bta-let-7b | ENSBTAG00000000950_MRPS16 |
| -0.38 | 0.0206 | bta-let-7b | ENSBTAG00000000957_CDKN2AIP |
| -0.33 | 0.0468 | bta-let-7b | ENSBTAG00000000972_KRTAP10-2 |
| -0.33 | 0.0468 | bta-let-7b | ENSBTAG00000000983_ABCC8 |
| -0.35 | 0.0341 | bta-let-7b | ENSBTAG00000000997 |
| -0.34 | 0.04 | bta-let-7b | ENSBTAG00000001097_FKBP7 |
| -0.36 | 0.029 | bta-let-7b | ENSBTAG00000001212_SSR2 |
| -0.33 | 0.0468 | bta-let-7b | ENSBTAG00000001329_CLPTM1L |
| -0.37 | 0.0245 | bta-let-7b | ENSBTAG00000001387_WDR73 |
| -0.34 | 0.04 | bta-let-7b | ENSBTAG00000001475_CCDC90A |
| -0.37 | 0.0245 | bta-let-7b | ENSBTAG00000001516_CRSP1 |
| -0.34 | 0.04 | bta-let-7b | ENSBTAG00000001527_TMEM213 |
| -0.36 | 0.029 | bta-let-7b | ENSBTAG00000001612_LSM1 |
| -0.34 | 0.04 | bta-let-7b | ENSBTAG00000001672 |
| -0.39 | 0.0173 | bta-let-7b | ENSBTAG00000001737_ZCCHC6 |
| -0.43 | 0.01 | bta-let-7b | ENSBTAG00000001784_LDLRAD2 |
| -0.35 | 0.0341 | bta-let-7b | ENSBTAG00000001810_SCAF11 |

1. **bta-let-7c.**





| **Correlation** | ***P* value** | **Correlated** | **With** |
| --- | --- | --- | --- |
| -0.35 | 0.0369 | bta-let-7c | ENSBTAG00000001500_FIGNL1 |
| -0.35 | 0.0369 | bta-let-7c | ENSBTAG00000001629_SCMH1 |

1. **bta-miR-103**





| **Correlation** | ***P* value** | **Correlated** | **With** |
| --- | --- | --- | --- |
| -0.34 | 0.0429 | bta-miR-103 | ENSBTAG00000001193_UNC93B1 |
| -0.34 | 0.0429 | bta-miR-103 | ENSBTAG00000001652_SLCO3A1 |

1. **bta-miR-106a**





| **Correlation** | ***P* value** | **Correlated** | **With** |
| --- | --- | --- | --- |
| -0.33 | 0.0468 | bta-miR-106a | ENSBTAG00000000222_ARID4B |
| -0.34 | 0.04 | bta-miR-106a | ENSBTAG00000000301 |
| -0.34 | 0.04 | bta-miR-106a | ENSBTAG00000000306_ITK |
| -0.34 | 0.04 | bta-miR-106a | ENSBTAG00000000478_LSMEM2 |
| -0.34 | 0.04 | bta-miR-106a | ENSBTAG00000000502_DAZL |
| -0.34 | 0.04 | bta-miR-106a | ENSBTAG00000000520_ATP2B3 |
| -0.34 | 0.04 | bta-miR-106a | ENSBTAG00000000679_PGM3 |
| -0.34 | 0.04 | bta-miR-106a | ENSBTAG00000000712_FBXW2 |
| -0.34 | 0.04 | bta-miR-106a | ENSBTAG00000001082_SH2D5 |
| -0.36 | 0.029 | bta-miR-106a | ENSBTAG00000001161_FAM60A |
| -0.35 | 0.0341 | bta-miR-106a | ENSBTAG00000001286_ELMO3 |
| -0.36 | 0.029 | bta-miR-106a | ENSBTAG00000001586_OTUD7B |
| -0.34 | 0.04 | bta-miR-106a | ENSBTAG00000001599_SV2A |

1. **bta-miR-125b**





| **Correlation** | ***P* value** | **Correlated** | **With** |
| --- | --- | --- | --- |
| -0.34 | 0.04 | bta-miR-125b | ENSBTAG00000000106_FAM69C |
| -0.33 | 0.0468 | bta-miR-125b | ENSBTAG00000001061_PRKCA |

1. **bta-miR-143**





| **Correlation** | ***P* value** | **Correlated** | **With** |
| --- | --- | --- | --- |
| -0.34 | 0.04 | bta-miR-143 | ENSBTAG00000000066_LRRN4 |
| -0.36 | 0.029 | bta-miR-143 | ENSBTAG00000000313_NUDT13 |
| -0.34 | 0.04 | bta-miR-143 | ENSBTAG00000000317_MAPK8IP1 |
| -0.34 | 0.04 | bta-miR-143 | ENSBTAG00000000414 |
| -0.38 | 0.0206 | bta-miR-143 | ENSBTAG00000000434_CRYAB |
| -0.38 | 0.0206 | bta-miR-143 | ENSBTAG00000000437_FFAR4 |
| -0.34 | 0.04 | bta-miR-143 | ENSBTAG00000000455_CREBZF |
| -0.38 | 0.0206 | bta-miR-143 | ENSBTAG00000000478_LSMEM2 |
| -0.36 | 0.029 | bta-miR-143 | ENSBTAG00000000494_PDE4D |
| -0.34 | 0.04 | bta-miR-143 | ENSBTAG00000000495_HAVCR2 |
| -0.43 | 0.01 | bta-miR-143 | ENSBTAG00000000505_CCBL2 |
| -0.34 | 0.04 | bta-miR-143 | ENSBTAG00000000654_ARMC4 |
| -0.38 | 0.0206 | bta-miR-143 | ENSBTAG00000000688 |
| -0.4 | 0.0145 | bta-miR-143 | ENSBTAG00000000795_NMNAT2 |
| -0.36 | 0.029 | bta-miR-143 | ENSBTAG00000000797_MRPL9 |
| -0.43 | 0.01 | bta-miR-143 | ENSBTAG00000000815_EPHA2 |
| -0.45 | 0.0068 | bta-miR-143 | ENSBTAG00000000843_NDRG2 |
| -0.38 | 0.0206 | bta-miR-143 | ENSBTAG00000001082_SH2D5 |
| -0.4 | 0.0145 | bta-miR-143 | ENSBTAG00000001573_JMJD1C |
| -0.38 | 0.0206 | bta-miR-143 | ENSBTAG00000001599_SV2A |
| -0.34 | 0.04 | bta-miR-143 | ENSBTAG00000001737_ZCCHC6 |

1. **bta-miR-186**


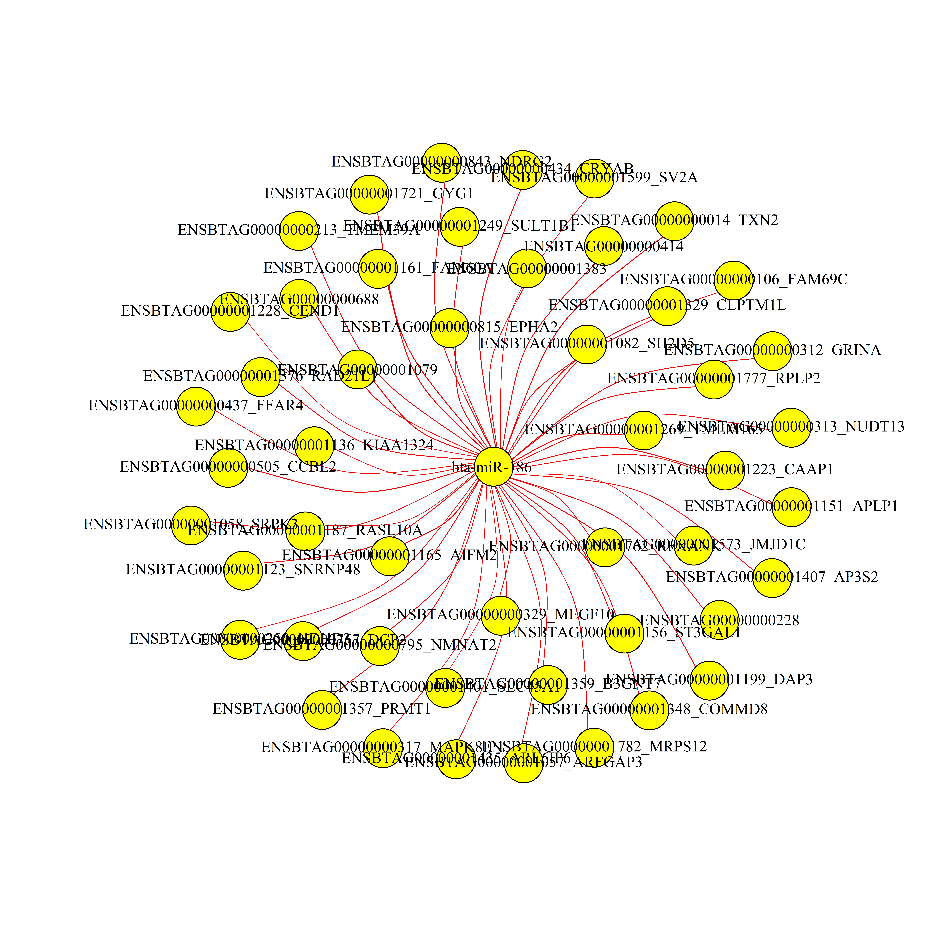


| **Correlation** | ***P* value** | **Correlated** | **With** |
| --- | --- | --- | --- |
| -0.33 | 0.0468 | bta-miR-186 | ENSBTAG00000000014_TXN2 |
| -0.38 | 0.0206 | bta-miR-186 | ENSBTAG00000000106_FAM69C |
| -0.34 | 0.0492 | bta-miR-186 | ENSBTAG00000000213_TMEM39A |
| -0.35 | 0.0341 | bta-miR-186 | ENSBTAG00000000228 |
| -0.39 | 0.0173 | bta-miR-186 | ENSBTAG00000000250_HDHD3 |
| -0.35 | 0.0341 | bta-miR-186 | ENSBTAG00000000312_GRINA |
| -0.37 | 0.0245 | bta-miR-186 | ENSBTAG00000000313_NUDT13 |
| -0.39 | 0.0173 | bta-miR-186 | ENSBTAG00000000317_MAPK8IP1 |
| -0.37 | 0.0245 | bta-miR-186 | ENSBTAG00000000329_MEGF10 |
| -0.35 | 0.0341 | bta-miR-186 | ENSBTAG00000000414 |
| -0.33 | 0.0468 | bta-miR-186 | ENSBTAG00000000434_CRYAB |
| -0.39 | 0.0173 | bta-miR-186 | ENSBTAG00000000437_FFAR4 |
| -0.33 | 0.0468 | bta-miR-186 | ENSBTAG00000000505_CCBL2 |
| -0.35 | 0.0341 | bta-miR-186 | ENSBTAG00000000688 |
| -0.35 | 0.0341 | bta-miR-186 | ENSBTAG00000000767_DCP2 |
| -0.37 | 0.0245 | bta-miR-186 | ENSBTAG00000000795_NMNAT2 |
| -0.35 | 0.0341 | bta-miR-186 | ENSBTAG00000000815_EPHA2 |
| -0.35 | 0.0341 | bta-miR-186 | ENSBTAG00000000843_NDRG2 |
| -0.37 | 0.0245 | bta-miR-186 | ENSBTAG00000001057_ARFGAP3 |
| -0.35 | 0.0341 | bta-miR-186 | ENSBTAG00000001058_SRPK3 |
| -0.33 | 0.0468 | bta-miR-186 | ENSBTAG00000001079 |
| -0.35 | 0.0341 | bta-miR-186 | ENSBTAG00000001082_SH2D5 |
| -0.35 | 0.0341 | bta-miR-186 | ENSBTAG00000001123_SNRNP48 |
| -0.39 | 0.0173 | bta-miR-186 | ENSBTAG00000001136_KIAA1324 |
| -0.36 | 0.029 | bta-miR-186 | ENSBTAG00000001151_APLP1 |
| -0.34 | 0.04 | bta-miR-186 | ENSBTAG00000001156_ST3GAL1 |
| -0.33 | 0.0468 | bta-miR-186 | ENSBTAG00000001161_FAM60A |
| -0.33 | 0.0468 | bta-miR-186 | ENSBTAG00000001165_AIFM2 |
| -0.42 | 0.0121 | bta-miR-186 | ENSBTAG00000001187_RASL10A |
| -0.33 | 0.0468 | bta-miR-186 | ENSBTAG00000001199_DAP3 |
| -0.33 | 0.0468 | bta-miR-186 | ENSBTAG00000001223_CAAP1 |
| -0.42 | 0.0121 | bta-miR-186 | ENSBTAG00000001228_CEND1 |
| -0.34 | 0.04 | bta-miR-186 | ENSBTAG00000001249_SULT1B1 |
| -0.33 | 0.0468 | bta-miR-186 | ENSBTAG00000001269_TMEM165 |
| -0.35 | 0.0341 | bta-miR-186 | ENSBTAG00000001329_CLPTM1L |
| -0.35 | 0.0335 | bta-miR-186 | ENSBTAG00000001348_COMMD8 |
| -0.37 | 0.0245 | bta-miR-186 | ENSBTAG00000001357_PRMT1 |
| -0.38 | 0.0206 | bta-miR-186 | ENSBTAG00000001359_B3GNT7 |
| -0.35 | 0.0341 | bta-miR-186 | ENSBTAG00000001376_RAD21L1 |
| -0.37 | 0.0245 | bta-miR-186 | ENSBTAG00000001383 |
| -0.42 | 0.0121 | bta-miR-186 | ENSBTAG00000001401_SLC45A1 |
| -0.36 | 0.029 | bta-miR-186 | ENSBTAG00000001407_AP3S2 |
| -0.36 | 0.029 | bta-miR-186 | ENSBTAG00000001435_ARL6IP6 |
| -0.44 | 0.0083 | bta-miR-186 | ENSBTAG00000001573_JMJD1C |
| -0.39 | 0.0173 | bta-miR-186 | ENSBTAG00000001599_SV2A |
| -0.33 | 0.0468 | bta-miR-186 | ENSBTAG00000001721_GYG1 |
| -0.39 | 0.0173 | bta-miR-186 | ENSBTAG00000001762_RFXANK |
| -0.35 | 0.0341 | bta-miR-186 | ENSBTAG00000001777_RPLP2 |
| -0.35 | 0.0341 | bta-miR-186 | ENSBTAG00000001782_MRPS12 |

1. **bta-miR-187**





| **Correlation** | ***P* value** | **Correlated** | **With** |
| --- | --- | --- | --- |
| -0.35 | 0.0487 | bta-miR-187 | ENSBTAG00000000827_TAOK1 |
| -0.35 | 0.0487 | bta-miR-187 | ENSBTAG00000000963 |
| -0.35 | 0.0487 | bta-miR-187 | ENSBTAG00000000995_FAM46A |

1. **bta-miR-191**


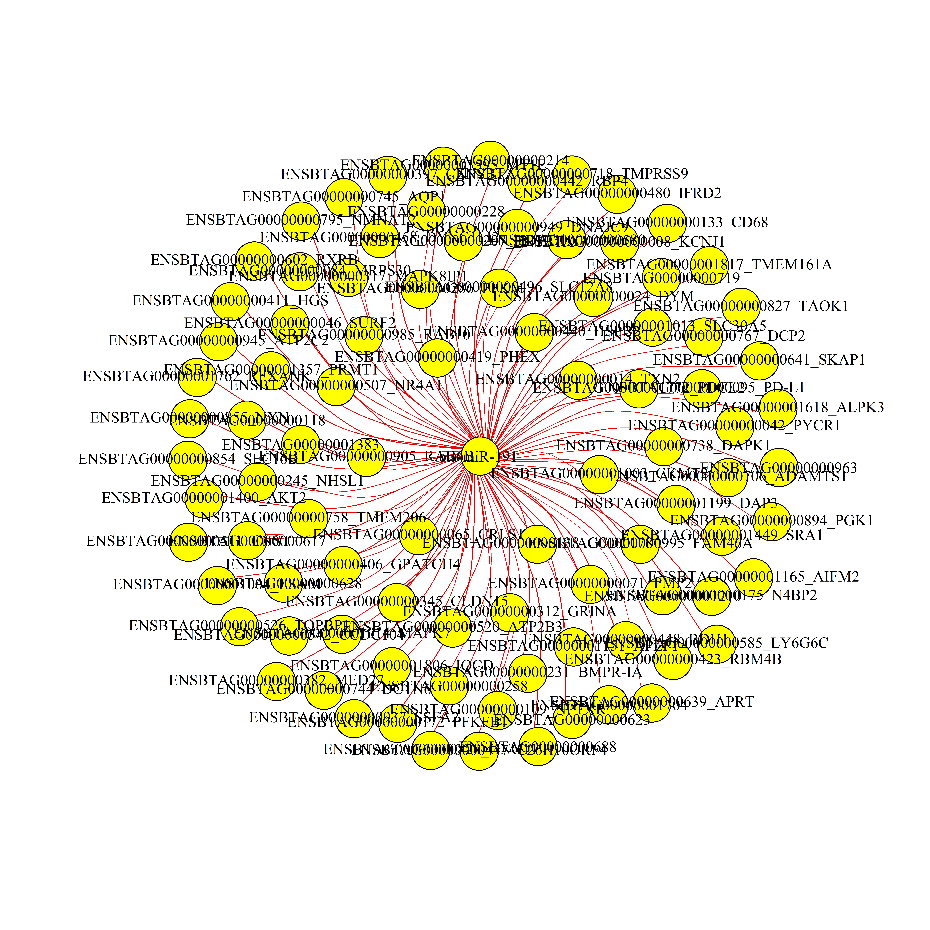


| **Correlation** | ***P* value** | **Correlated** | **With** |
| --- | --- | --- | --- |
| -0.34 | 0.0432 | bta-miR-191 | ENSBTAG00000000008_KCNJ1 |
| -0.42 | 0.011 | bta-miR-191 | ENSBTAG00000000014_TXN2 |
| -0.36 | 0.0313 | bta-miR-191 | ENSBTAG00000000024_DYM |
| -0.41 | 0.0132 | bta-miR-191 | ENSBTAG00000000042_PYCR1 |
| -0.37 | 0.0266 | bta-miR-191 | ENSBTAG00000000046_SURF2 |
| -0.41 | 0.0132 | bta-miR-191 | ENSBTAG00000000065_CRLS1 |
| -0.34 | 0.0432 | bta-miR-191 | ENSBTAG00000000071_PMP2 |
| -0.41 | 0.0132 | bta-miR-191 | ENSBTAG00000000084_MRPS30 |
| -0.38 | 0.0224 | bta-miR-191 | ENSBTAG00000000095_PD-L1 |
| -0.43 | 0.0091 | bta-miR-191 | ENSBTAG00000000109_NPTXR |
| -0.35 | 0.0369 | bta-miR-191 | ENSBTAG00000000118 |
| -0.46 | 0.005 | bta-miR-191 | ENSBTAG00000000133_CD68 |
| -0.35 | 0.0369 | bta-miR-191 | ENSBTAG00000000138_CCDC178 |
| -0.34 | 0.0432 | bta-miR-191 | ENSBTAG00000000172_PFKFB1 |
| -0.36 | 0.0313 | bta-miR-191 | ENSBTAG00000000175_N4BP2 |
| -0.37 | 0.0266 | bta-miR-191 | ENSBTAG00000000207_HDHD1A |
| -0.36 | 0.0313 | bta-miR-191 | ENSBTAG00000000214 |
| -0.46 | 0.005 | bta-miR-191 | ENSBTAG00000000228 |
| -0.39 | 0.0189 | bta-miR-191 | ENSBTAG00000000231_BMPR-IA |
| -0.45 | 0.0061 | bta-miR-191 | ENSBTAG00000000245_NHSL1 |
| -0.4 | 0.0158 | bta-miR-191 | ENSBTAG00000000258 |
| -0.39 | 0.0189 | bta-miR-191 | ENSBTAG00000000286_PFKM |
| -0.34 | 0.0432 | bta-miR-191 | ENSBTAG00000000312_GRINA |
| -0.38 | 0.0224 | bta-miR-191 | ENSBTAG00000000317_MAPK8IP1 |
| -0.34 | 0.0432 | bta-miR-191 | ENSBTAG00000000345_CLDN15 |
| -0.39 | 0.0189 | bta-miR-191 | ENSBTAG00000000382_MED27 |
| -0.34 | 0.0432 | bta-miR-191 | ENSBTAG00000000397_CNPY1 |
| -0.35 | 0.0369 | bta-miR-191 | ENSBTAG00000000406_GPATCH4 |
| -0.35 | 0.0369 | bta-miR-191 | ENSBTAG00000000411_HGS |
| -0.34 | 0.0432 | bta-miR-191 | ENSBTAG00000000419_PHEX |
| -0.39 | 0.0189 | bta-miR-191 | ENSBTAG00000000423_RBM4B |
| -0.34 | 0.0432 | bta-miR-191 | ENSBTAG00000000440_HSPB2 |
| -0.34 | 0.0432 | bta-miR-191 | ENSBTAG00000000442_RBP4 |
| -0.37 | 0.0266 | bta-miR-191 | ENSBTAG00000000447_C26H10ORF4 |
| -0.34 | 0.0432 | bta-miR-191 | ENSBTAG00000000448_BDH1 |
| -0.41 | 0.0132 | bta-miR-191 | ENSBTAG00000000458_DYNC1LI2 |
| -0.37 | 0.0266 | bta-miR-191 | ENSBTAG00000000480_IFRD2 |
| -0.46 | 0.005 | bta-miR-191 | ENSBTAG00000000496_SLC12A8 |
| -0.36 | 0.0313 | bta-miR-191 | ENSBTAG00000000507_NR4A1 |
| -0.37 | 0.0266 | bta-miR-191 | ENSBTAG00000000511_CNST |
| -0.37 | 0.0266 | bta-miR-191 | ENSBTAG00000000520_ATP2B3 |
| -0.41 | 0.0132 | bta-miR-191 | ENSBTAG00000000526_TOPBP1 |
| -0.37 | 0.0266 | bta-miR-191 | ENSBTAG00000000542_CCDC104 |
| -0.38 | 0.0224 | bta-miR-191 | ENSBTAG00000000580 |
| -0.37 | 0.0266 | bta-miR-191 | ENSBTAG00000000585_LY6G6C |
| -0.36 | 0.0313 | bta-miR-191 | ENSBTAG00000000602_RXRB |
| -0.44 | 0.0075 | bta-miR-191 | ENSBTAG00000000617 |
| -0.38 | 0.0224 | bta-miR-191 | ENSBTAG00000000623 |
| -0.43 | 0.0091 | bta-miR-191 | ENSBTAG00000000628 |
| -0.53 | 0.0014 | bta-miR-191 | ENSBTAG00000000639_APRT |
| -0.41 | 0.0132 | bta-miR-191 | ENSBTAG00000000641_SKAP1 |
| -0.34 | 0.0432 | bta-miR-191 | ENSBTAG00000000688 |
| -0.36 | 0.0308 | bta-miR-191 | ENSBTAG00000000706_ADAMTS1 |
| -0.44 | 0.0075 | bta-miR-191 | ENSBTAG00000000718_TMPRSS9 |
| -0.36 | 0.0313 | bta-miR-191 | ENSBTAG00000000719 |
| -0.39 | 0.0189 | bta-miR-191 | ENSBTAG00000000738_DAPK1 |
| -0.38 | 0.0224 | bta-miR-191 | ENSBTAG00000000744_DCTN6 |
| -0.43 | 0.0091 | bta-miR-191 | ENSBTAG00000000745_AQP1 |
| -0.43 | 0.0091 | bta-miR-191 | ENSBTAG00000000758_TMEM206 |
| -0.4 | 0.0158 | bta-miR-191 | ENSBTAG00000000767_DCP2 |
| -0.34 | 0.0432 | bta-miR-191 | ENSBTAG00000000795_NMNAT2 |
| -0.39 | 0.0189 | bta-miR-191 | ENSBTAG00000000820_GNG11 |
| -0.45 | 0.0061 | bta-miR-191 | ENSBTAG00000000827_TAOK1 |
| -0.4 | 0.0158 | bta-miR-191 | ENSBTAG00000000854_SEC16B |
| -0.36 | 0.0313 | bta-miR-191 | ENSBTAG00000000855_NXN |
| -0.42 | 0.011 | bta-miR-191 | ENSBTAG00000000894_PGK1 |
| -0.35 | 0.0369 | bta-miR-191 | ENSBTAG00000000905_RAB6B |
| -0.4 | 0.0158 | bta-miR-191 | ENSBTAG00000000937_SSFA2 |
| -0.36 | 0.0313 | bta-miR-191 | ENSBTAG00000000945_ATP2C2 |
| -0.34 | 0.0432 | bta-miR-191 | ENSBTAG00000000949_DNAJC9 |
| -0.39 | 0.0189 | bta-miR-191 | ENSBTAG00000000963 |
| -0.37 | 0.0266 | bta-miR-191 | ENSBTAG00000000985_RAB10 |
| -0.39 | 0.0189 | bta-miR-191 | ENSBTAG00000000995_FAM46A |
| -0.48 | 0.0041 | bta-miR-191 | ENSBTAG00000001003_CKMT2 |
| -0.39 | 0.0189 | bta-miR-191 | ENSBTAG00000001004_ESAM |
| -0.38 | 0.0224 | bta-miR-191 | ENSBTAG00000001013_SLC30A5 |
| -0.38 | 0.0224 | bta-miR-191 | ENSBTAG00000001014_MAPK7 |
| -0.35 | 0.0369 | bta-miR-191 | ENSBTAG00000001151_APLP1 |
| -0.4 | 0.0158 | bta-miR-191 | ENSBTAG00000001165_AIFM2 |
| -0.34 | 0.0432 | bta-miR-191 | ENSBTAG00000001199_DAP3 |
| -0.39 | 0.0189 | bta-miR-191 | ENSBTAG00000001210 |
| -0.36 | 0.0313 | bta-miR-191 | ENSBTAG00000001357_PRMT1 |
| -0.4 | 0.0158 | bta-miR-191 | ENSBTAG00000001383 |
| -0.37 | 0.0266 | bta-miR-191 | ENSBTAG00000001400_AKT2 |
| -0.39 | 0.0189 | bta-miR-191 | ENSBTAG00000001449_SRA1 |
| -0.37 | 0.0266 | bta-miR-191 | ENSBTAG00000001595_MT1E |
| -0.39 | 0.0189 | bta-miR-191 | ENSBTAG00000001618_ALPK3 |
| -0.42 | 0.011 | bta-miR-191 | ENSBTAG00000001704 |
| -0.36 | 0.0313 | bta-miR-191 | ENSBTAG00000001762_RFXANK |
| -0.35 | 0.0369 | bta-miR-191 | ENSBTAG00000001772_PDCL2 |
| -0.36 | 0.0313 | bta-miR-191 | ENSBTAG00000001806_IQCD |
| -0.38 | 0.0224 | bta-miR-191 | ENSBTAG00000001817_TMEM161A |

1. **bta-miR-193a-5p**


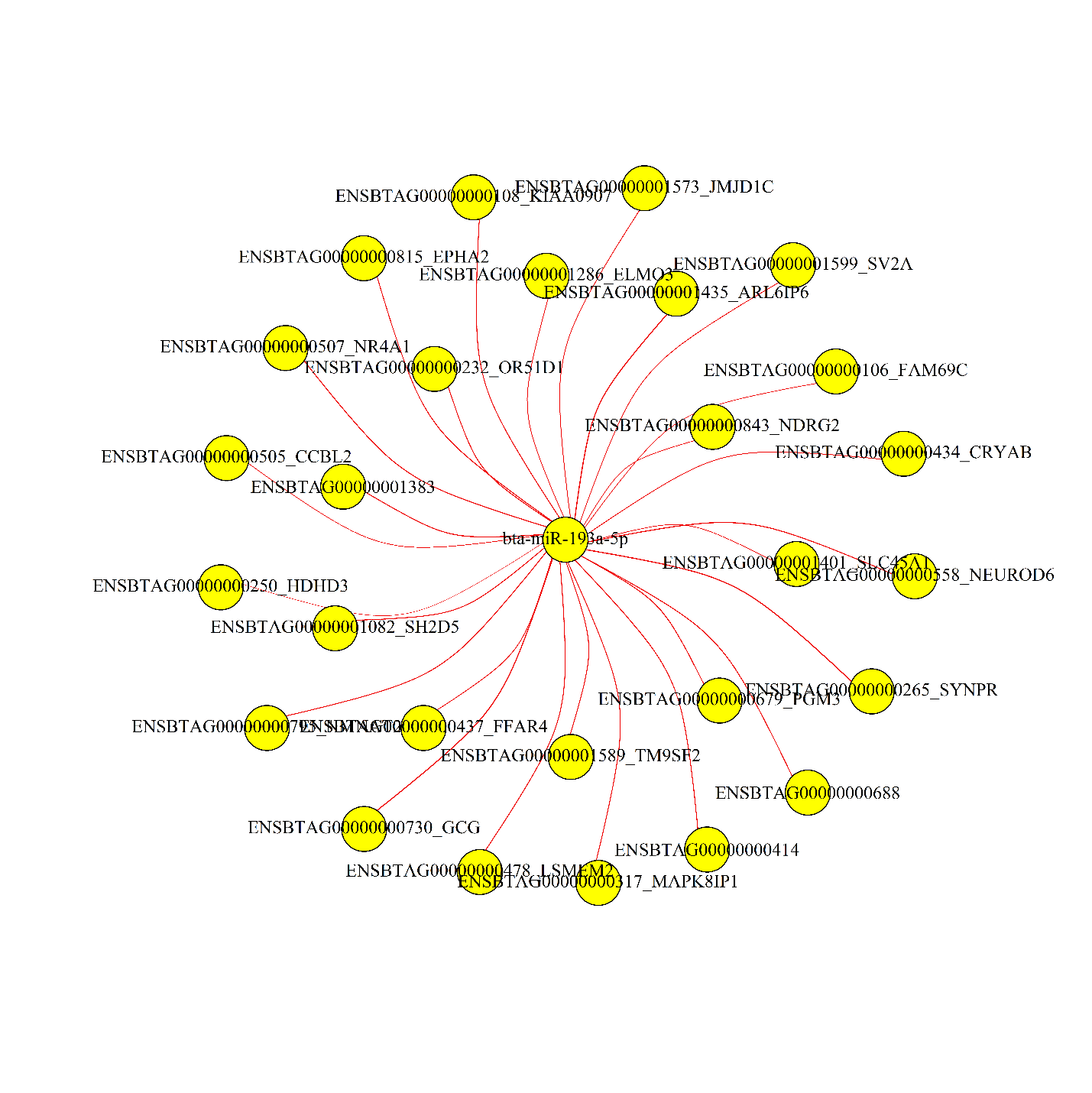


| **Correlation** | ***P* value** | **Correlated** | **With** |
| --- | --- | --- | --- |
| -0.44 | 0.0083 | bta-miR-193a-5p | ENSBTAG00000000106_FAM69C |
| -0.36 | 0.029 | bta-miR-193a-5p | ENSBTAG00000000108_KIAA0907 |
| -0.38 | 0.0206 | bta-miR-193a-5p | ENSBTAG00000000232_OR51D1 |
| -0.51 | 0.002 | bta-miR-193a-5p | ENSBTAG00000000250_HDHD3 |
| -0.33 | 0.0468 | bta-miR-193a-5p | ENSBTAG00000000265_SYNPR |
| -0.38 | 0.0206 | bta-miR-193a-5p | ENSBTAG00000000317_MAPK8IP1 |
| -0.38 | 0.0206 | bta-miR-193a-5p | ENSBTAG00000000414 |
| -0.4 | 0.0145 | bta-miR-193a-5p | ENSBTAG00000000434_CRYAB |
| -0.4 | 0.0145 | bta-miR-193a-5p | ENSBTAG00000000437_FFAR4 |
| -0.38 | 0.0206 | bta-miR-193a-5p | ENSBTAG00000000478_LSMEM2 |
| -0.45 | 0.0068 | bta-miR-193a-5p | ENSBTAG00000000505_CCBL2 |
| -0.34 | 0.04 | bta-miR-193a-5p | ENSBTAG00000000507_NR4A1 |
| -0.34 | 0.04 | bta-miR-193a-5p | ENSBTAG00000000558_NEUROD6 |
| -0.34 | 0.04 | bta-miR-193a-5p | ENSBTAG00000000679_PGM3 |
| -0.36 | 0.029 | bta-miR-193a-5p | ENSBTAG00000000688 |
| -0.33 | 0.0468 | bta-miR-193a-5p | ENSBTAG00000000730_GCG |
| -0.38 | 0.0206 | bta-miR-193a-5p | ENSBTAG00000000795_NMNAT2 |
| -0.38 | 0.0206 | bta-miR-193a-5p | ENSBTAG00000000815_EPHA2 |
| -0.43 | 0.01 | bta-miR-193a-5p | ENSBTAG00000000843_NDRG2 |
| -0.38 | 0.0206 | bta-miR-193a-5p | ENSBTAG00000001082_SH2D5 |
| -0.39 | 0.0173 | bta-miR-193a-5p | ENSBTAG00000001286_ELMO3 |
| -0.34 | 0.04 | bta-miR-193a-5p | ENSBTAG00000001383 |
| -0.43 | 0.01 | bta-miR-193a-5p | ENSBTAG00000001401_SLC45A1 |
| -0.33 | 0.0468 | bta-miR-193a-5p | ENSBTAG00000001435_ARL6IP6 |
| -0.4 | 0.0145 | bta-miR-193a-5p | ENSBTAG00000001573_JMJD1C |
| -0.36 | 0.029 | bta-miR-193a-5p | ENSBTAG00000001589_TM9SF2 |
| -0.4 | 0.0145 | bta-miR-193a-5p | ENSBTAG00000001599_SV2A |

1. **bta-miR-19b.**





| **Correlation** | ***P* value** | **Correlated** | **With** |  |  |
| --- | --- | --- | --- | --- | --- |
| -0.35 | 0.0341 | bta-miR-19b | ENSBTAG00000000012_TTC33 | | |
| -0.37 | 0.0245 | bta-miR-19b | ENSBTAG00000000066_LRRN4 | | |
| -0.37 | 0.0245 | bta-miR-19b | ENSBTAG00000000074_NFIA | | |
| -0.33 | 0.0468 | bta-miR-19b | ENSBTAG00000000259_CHIA | | |
| -0.35 | 0.0341 | bta-miR-19b | ENSBTAG00000000305_LMO4 | | |
| -0.33 | 0.0468 | bta-miR-19b | ENSBTAG00000000306_ITK | | |
| -0.33 | 0.0468 | bta-miR-19b | ENSBTAG00000000611_C16orf90 | | |
| -0.33 | 0.0468 | bta-miR-19b | ENSBTAG00000000688 | | |
| -0.35 | 0.0341 | bta-miR-19b | ENSBTAG00000001290_TUBD1 | | |
| -0.33 | 0.0468 | bta-miR-19b | ENSBTAG00000001324_SLCO2A1 | | |
| -0.33 | 0.0468 | bta-miR-19b | ENSBTAG00000001343_DEPDC1 | | |
| -0.35 | 0.0341 | bta-miR-19b | ENSBTAG00000001450_APBB3 | | |
| -0.42 | 0.0121 | bta-miR-19b | ENSBTAG00000001451_A4GNT | | |
| -0.39 | 0.0173 | bta-miR-19b | ENSBTAG00000001518_ISG20L2 | | |
| -0.33 | 0.0468 | bta-miR-19b | ENSBTAG00000001803_FHL5 | | |
| -0.37 | 0.0245 | bta-miR-19b | ENSBTAG00000001809_DUSP21 | | |

1. **bta-miR-200b**





| **Correlation** | ***P* value** | **Correlated** | **With** |
| --- | --- | --- | --- |
| -0.36 | 0.029 | bta-miR-200b | ENSBTAG00000000489_WDR54 |
| -0.34 | 0.04 | bta-miR-200b | ENSBTAG00000000797_MRPL9 |
| -0.34 | 0.04 | bta-miR-200b | ENSBTAG00000000925_GLT8D2 |

1. **bta-miR-222**


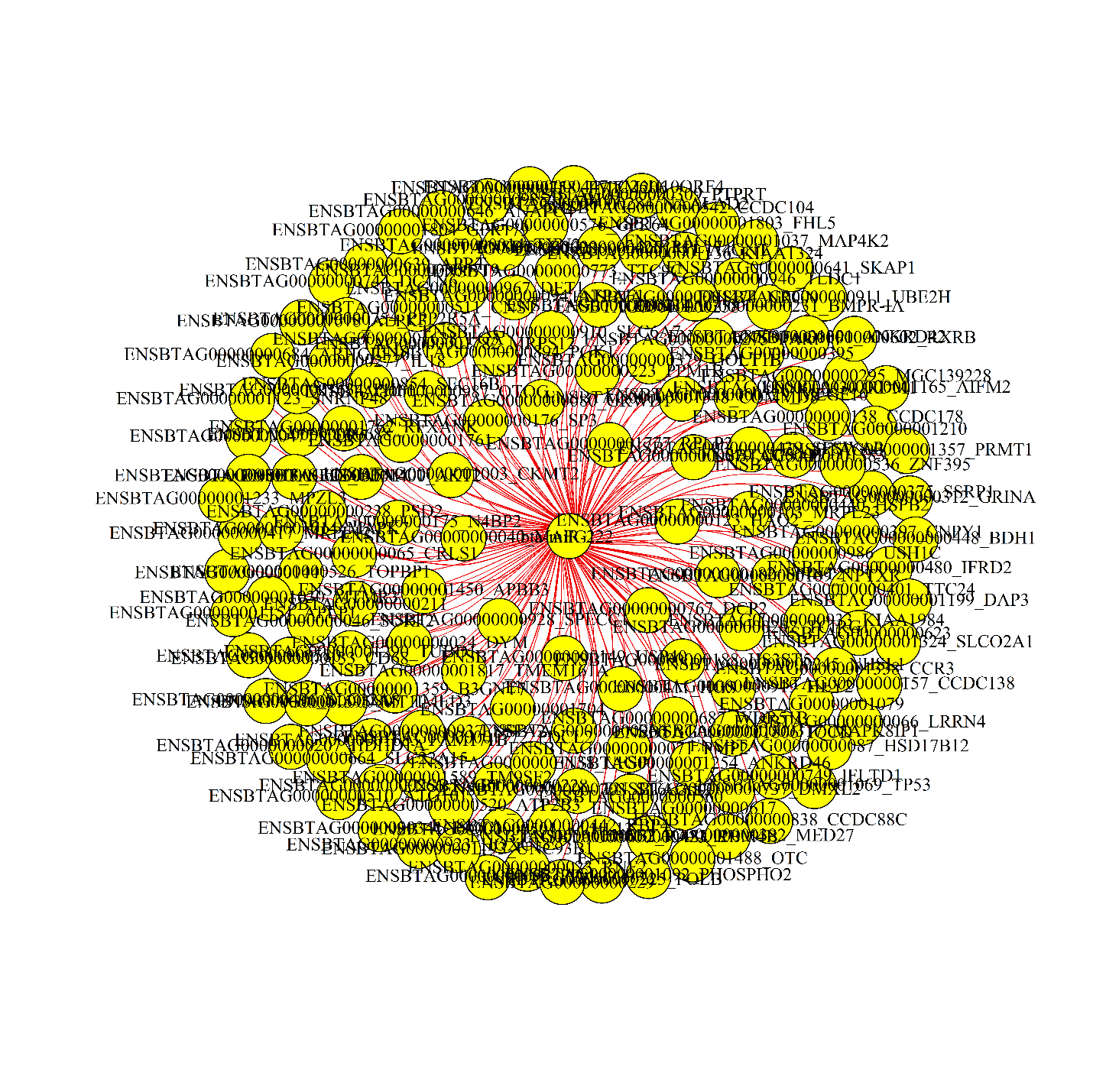


| **Correlation** | ***P* value** | **Correlated** | **With** |
| --- | --- | --- | --- |
| -0.5 | 0.0027 | bta-miR-222 | ENSBTAG00000000014_TXN2 |
| -0.35 | 0.0369 | bta-miR-222 | ENSBTAG00000000023_RNF2 |
| -0.41 | 0.0132 | bta-miR-222 | ENSBTAG00000000024_DYM |
| -0.36 | 0.0313 | bta-miR-222 | ENSBTAG00000000030_RDM1 |
| -0.42 | 0.011 | bta-miR-222 | ENSBTAG00000000040_MAFG |
| -0.42 | 0.011 | bta-miR-222 | ENSBTAG00000000046_SURF2 |
| -0.34 | 0.0432 | bta-miR-222 | ENSBTAG00000000062_RASL12 |
| -0.34 | 0.0432 | bta-miR-222 | ENSBTAG00000000065_CRLS1 |
| -0.35 | 0.0369 | bta-miR-222 | ENSBTAG00000000066_LRRN4 |
| -0.41 | 0.0132 | bta-miR-222 | ENSBTAG00000000071_PMP2 |
| -0.38 | 0.0224 | bta-miR-222 | ENSBTAG00000000079_CCSAP |
| -0.39 | 0.0189 | bta-miR-222 | ENSBTAG00000000080_GRWD1 |
| -0.34 | 0.0432 | bta-miR-222 | ENSBTAG00000000087_HSD17B12 |
| -0.34 | 0.0432 | bta-miR-222 | ENSBTAG00000000094_ATPAF1 |
| -0.48 | 0.0041 | bta-miR-222 | ENSBTAG00000000095_PD-L1 |
| -0.36 | 0.0313 | bta-miR-222 | ENSBTAG00000000103_MRPL23 |
| -0.51 | 0.0022 | bta-miR-222 | ENSBTAG00000000109_NPTXR |
| -0.34 | 0.0432 | bta-miR-222 | ENSBTAG00000000123_HAO2 |
| -0.45 | 0.0061 | bta-miR-222 | ENSBTAG00000000133_CD68 |
| -0.42 | 0.011 | bta-miR-222 | ENSBTAG00000000138_CCDC178 |
| -0.39 | 0.0189 | bta-miR-222 | ENSBTAG00000000144 |
| -0.4 | 0.0158 | bta-miR-222 | ENSBTAG00000000149_USP40 |
| -0.34 | 0.0432 | bta-miR-222 | ENSBTAG00000000157_CCDC138 |
| -0.45 | 0.0061 | bta-miR-222 | ENSBTAG00000000175_N4BP2 |
| -0.34 | 0.0432 | bta-miR-222 | ENSBTAG00000000176_SP3 |
| -0.38 | 0.0224 | bta-miR-222 | ENSBTAG00000000182_SPINT2 |
| -0.36 | 0.0313 | bta-miR-222 | ENSBTAG00000000188_HS3ST5 |
| -0.4 | 0.0158 | bta-miR-222 | ENSBTAG00000000207_HDHD1A |
| -0.39 | 0.0189 | bta-miR-222 | ENSBTAG00000000211 |
| -0.35 | 0.0369 | bta-miR-222 | ENSBTAG00000000214 |
| -0.36 | 0.0313 | bta-miR-222 | ENSBTAG00000000223_PPM1B |
| -0.34 | 0.0432 | bta-miR-222 | ENSBTAG00000000225_POLB |
| -0.5 | 0.0027 | bta-miR-222 | ENSBTAG00000000228 |
| -0.35 | 0.0369 | bta-miR-222 | ENSBTAG00000000229 |
| -0.4 | 0.0158 | bta-miR-222 | ENSBTAG00000000231_BMPR-IA |
| -0.41 | 0.0132 | bta-miR-222 | ENSBTAG00000000238_PSD2 |
| -0.53 | 0.0014 | bta-miR-222 | ENSBTAG00000000245_NHSL1 |
| -0.39 | 0.0189 | bta-miR-222 | ENSBTAG00000000258 |
| -0.37 | 0.0266 | bta-miR-222 | ENSBTAG00000000266_NAB1 |
| -0.34 | 0.0432 | bta-miR-222 | ENSBTAG00000000275_PARP11 |
| -0.36 | 0.0313 | bta-miR-222 | ENSBTAG00000000277_IL18 |
| -0.44 | 0.0075 | bta-miR-222 | ENSBTAG00000000284_NAALAD2 |
| -0.38 | 0.0224 | bta-miR-222 | ENSBTAG00000000292_TCIRG1 |
| -0.36 | 0.0313 | bta-miR-222 | ENSBTAG00000000295_MGC139228 |
| -0.35 | 0.0369 | bta-miR-222 | ENSBTAG00000000309_PTPRT |
| -0.43 | 0.0091 | bta-miR-222 | ENSBTAG00000000312_GRINA |
| -0.39 | 0.0189 | bta-miR-222 | ENSBTAG00000000317_MAPK8IP1 |
| -0.39 | 0.0189 | bta-miR-222 | ENSBTAG00000000329_MEGF10 |
| -0.4 | 0.0158 | bta-miR-222 | ENSBTAG00000000341_ISOC1 |
| -0.34 | 0.0432 | bta-miR-222 | ENSBTAG00000000375_SSRP1 |
| -0.34 | 0.0432 | bta-miR-222 | ENSBTAG00000000379_GOLT1B |
| -0.36 | 0.0313 | bta-miR-222 | ENSBTAG00000000382_MED27 |
| -0.36 | 0.0313 | bta-miR-222 | ENSBTAG00000000395 |
| -0.45 | 0.0061 | bta-miR-222 | ENSBTAG00000000397_CNPY1 |
| -0.39 | 0.0189 | bta-miR-222 | ENSBTAG00000000401_TTC24 |
| -0.34 | 0.0432 | bta-miR-222 | ENSBTAG00000000411_HGS |
| -0.39 | 0.0189 | bta-miR-222 | ENSBTAG00000000417_MRPL12 |
| -0.42 | 0.011 | bta-miR-222 | ENSBTAG00000000423_RBM4B |
| -0.35 | 0.0369 | bta-miR-222 | ENSBTAG00000000425_RPL17 |
| -0.34 | 0.0432 | bta-miR-222 | ENSBTAG00000000439_SFSWAP |
| -0.41 | 0.0132 | bta-miR-222 | ENSBTAG00000000440_HSPB2 |
| -0.41 | 0.0132 | bta-miR-222 | ENSBTAG00000000442_RBP4 |
| -0.42 | 0.011 | bta-miR-222 | ENSBTAG00000000447_C26H10ORF4 |
| -0.37 | 0.0266 | bta-miR-222 | ENSBTAG00000000448_BDH1 |
| -0.35 | 0.0369 | bta-miR-222 | ENSBTAG00000000477_TLR5 |
| -0.38 | 0.0224 | bta-miR-222 | ENSBTAG00000000480_IFRD2 |
| -0.45 | 0.0061 | bta-miR-222 | ENSBTAG00000000496_SLC12A8 |
| -0.38 | 0.0224 | bta-miR-222 | ENSBTAG00000000501_KLHL13 |
| -0.44 | 0.0075 | bta-miR-222 | ENSBTAG00000000510_ATG101 |
| -0.38 | 0.0224 | bta-miR-222 | ENSBTAG00000000511_CNST |
| -0.34 | 0.0432 | bta-miR-222 | ENSBTAG00000000520_ATP2B3 |
| -0.44 | 0.0075 | bta-miR-222 | ENSBTAG00000000526_TOPBP1 |
| -0.35 | 0.0354 | bta-miR-222 | ENSBTAG00000000536_ZNF395 |
| -0.4 | 0.0158 | bta-miR-222 | ENSBTAG00000000542_CCDC104 |
| -0.37 | 0.0266 | bta-miR-222 | ENSBTAG00000000560 |
| -0.45 | 0.0092 | bta-miR-222 | ENSBTAG00000000576_GPR64 |
| -0.41 | 0.0132 | bta-miR-222 | ENSBTAG00000000580 |
| -0.35 | 0.0369 | bta-miR-222 | ENSBTAG00000000602_RXRB |
| -0.37 | 0.0266 | bta-miR-222 | ENSBTAG00000000617 |
| -0.45 | 0.0061 | bta-miR-222 | ENSBTAG00000000623 |
| -0.36 | 0.0313 | bta-miR-222 | ENSBTAG00000000628 |
| -0.41 | 0.0132 | bta-miR-222 | ENSBTAG00000000639_APRT |
| -0.42 | 0.011 | bta-miR-222 | ENSBTAG00000000641_SKAP1 |
| -0.35 | 0.0369 | bta-miR-222 | ENSBTAG00000000646_ANAPC4 |
| -0.4 | 0.0158 | bta-miR-222 | ENSBTAG00000000664_SLC22A15 |
| -0.37 | 0.0266 | bta-miR-222 | ENSBTAG00000000684_ARHGEF10L |
| -0.36 | 0.0313 | bta-miR-222 | ENSBTAG00000000687_WDR51B |
| -0.37 | 0.0266 | bta-miR-222 | ENSBTAG00000000688 |
| -0.34 | 0.0432 | bta-miR-222 | ENSBTAG00000000703_ST6GAL2 |
| -0.45 | 0.0061 | bta-miR-222 | ENSBTAG00000000719 |
| -0.37 | 0.0266 | bta-miR-222 | ENSBTAG00000000737_DMXL2 |
| -0.41 | 0.0132 | bta-miR-222 | ENSBTAG00000000744_DCTN6 |
| -0.34 | 0.0432 | bta-miR-222 | ENSBTAG00000000749_IFLTD1 |
| -0.37 | 0.0266 | bta-miR-222 | ENSBTAG00000000754_PPP2R5A |
| -0.51 | 0.0022 | bta-miR-222 | ENSBTAG00000000758_TMEM206 |
| -0.45 | 0.0061 | bta-miR-222 | ENSBTAG00000000767_DCP2 |
| -0.34 | 0.0432 | bta-miR-222 | ENSBTAG00000000773_TTC9C |
| -0.35 | 0.0369 | bta-miR-222 | ENSBTAG00000000807_TMED3 |
| -0.34 | 0.0432 | bta-miR-222 | ENSBTAG00000000838_CCDC88C |
| -0.43 | 0.0091 | bta-miR-222 | ENSBTAG00000000854_SEC16B |
| -0.48 | 0.0041 | bta-miR-222 | ENSBTAG00000000894_PGK1 |
| -0.37 | 0.0266 | bta-miR-222 | ENSBTAG00000000910_SLC6A7 |
| -0.38 | 0.0224 | bta-miR-222 | ENSBTAG00000000911_UBE2H |
| -0.34 | 0.0432 | bta-miR-222 | ENSBTAG00000000919_HEY2 |
| -0.38 | 0.0224 | bta-miR-222 | ENSBTAG00000000923_HOXC13 |
| -0.4 | 0.0158 | bta-miR-222 | ENSBTAG00000000928_SPECC1 |
| -0.38 | 0.0224 | bta-miR-222 | ENSBTAG00000000933_KIAA1984 |
| -0.37 | 0.0266 | bta-miR-222 | ENSBTAG00000000937_SSFA2 |
| -0.38 | 0.0224 | bta-miR-222 | ENSBTAG00000000946_TLDC1 |
| -0.44 | 0.0075 | bta-miR-222 | ENSBTAG00000000966 |
| -0.37 | 0.0266 | bta-miR-222 | ENSBTAG00000000967_DET1 |
| -0.34 | 0.0432 | bta-miR-222 | ENSBTAG00000000985_RAB10 |
| -0.35 | 0.0369 | bta-miR-222 | ENSBTAG00000000986_USH1C |
| -0.34 | 0.0432 | bta-miR-222 | ENSBTAG00000000987_OTOG |
| -0.38 | 0.0224 | bta-miR-222 | ENSBTAG00000001003_CKMT2 |
| -0.48 | 0.0041 | bta-miR-222 | ENSBTAG00000001013_SLC30A5 |
| -0.35 | 0.0369 | bta-miR-222 | ENSBTAG00000001014_MAPK7 |
| -0.39 | 0.0189 | bta-miR-222 | ENSBTAG00000001030_MTMR3 |
| -0.39 | 0.0189 | bta-miR-222 | ENSBTAG00000001037_MAP4K2 |
| -0.39 | 0.0189 | bta-miR-222 | ENSBTAG00000001069_TP53 |
| -0.39 | 0.0189 | bta-miR-222 | ENSBTAG00000001079 |
| -0.39 | 0.0189 | bta-miR-222 | ENSBTAG00000001092_PHOSPHO2 |
| -0.35 | 0.0369 | bta-miR-222 | ENSBTAG00000001123_SNRNP48 |
| -0.35 | 0.0369 | bta-miR-222 | ENSBTAG00000001136_KIAA1324 |
| -0.38 | 0.0224 | bta-miR-222 | ENSBTAG00000001138_UFSP1 |
| -0.42 | 0.011 | bta-miR-222 | ENSBTAG00000001151_APLP1 |
| -0.48 | 0.0041 | bta-miR-222 | ENSBTAG00000001165_AIFM2 |
| -0.38 | 0.0224 | bta-miR-222 | ENSBTAG00000001193_UNC93B1 |
| -0.35 | 0.0369 | bta-miR-222 | ENSBTAG00000001199_DAP3 |
| -0.4 | 0.0158 | bta-miR-222 | ENSBTAG00000001210 |
| -0.39 | 0.0189 | bta-miR-222 | ENSBTAG00000001233_MPZL3 |
| -0.38 | 0.0224 | bta-miR-222 | ENSBTAG00000001254_ANKRD46 |
| -0.4 | 0.0158 | bta-miR-222 | ENSBTAG00000001290_TUBD1 |
| -0.36 | 0.0313 | bta-miR-222 | ENSBTAG00000001323_CENPC1 |
| -0.38 | 0.0224 | bta-miR-222 | ENSBTAG00000001324_SLCO2A1 |
| -0.35 | 0.0369 | bta-miR-222 | ENSBTAG00000001338_CCR3 |
| -0.35 | 0.0362 | bta-miR-222 | ENSBTAG00000001348_COMMD8 |
| -0.43 | 0.0091 | bta-miR-222 | ENSBTAG00000001357_PRMT1 |
| -0.36 | 0.0313 | bta-miR-222 | ENSBTAG00000001359_B3GNT7 |
| -0.45 | 0.0061 | bta-miR-222 | ENSBTAG00000001383 |
| -0.35 | 0.0369 | bta-miR-222 | ENSBTAG00000001391_ANKRD42 |
| -0.4 | 0.0158 | bta-miR-222 | ENSBTAG00000001400_AKT2 |
| -0.4 | 0.0158 | bta-miR-222 | ENSBTAG00000001450_APBB3 |
| -0.37 | 0.0266 | bta-miR-222 | ENSBTAG00000001451_A4GNT |
| -0.4 | 0.0158 | bta-miR-222 | ENSBTAG00000001474_NRGN |
| -0.36 | 0.0313 | bta-miR-222 | ENSBTAG00000001488_OTC |
| -0.34 | 0.0432 | bta-miR-222 | ENSBTAG00000001589_TM9SF2 |
| -0.4 | 0.0158 | bta-miR-222 | ENSBTAG00000001595_MT1E |
| -0.42 | 0.011 | bta-miR-222 | ENSBTAG00000001618_ALPK3 |
| -0.46 | 0.005 | bta-miR-222 | ENSBTAG00000001634_PHF1 |
| -0.34 | 0.0432 | bta-miR-222 | ENSBTAG00000001638_FGA |
| -0.34 | 0.0432 | bta-miR-222 | ENSBTAG00000001683_FAM171B |
| -0.45 | 0.0061 | bta-miR-222 | ENSBTAG00000001704 |
| -0.42 | 0.011 | bta-miR-222 | ENSBTAG00000001761 |
| -0.48 | 0.0041 | bta-miR-222 | ENSBTAG00000001762_RFXANK |
| -0.44 | 0.0075 | bta-miR-222 | ENSBTAG00000001772_PDCL2 |
| -0.35 | 0.0369 | bta-miR-222 | ENSBTAG00000001777_RPLP2 |
| -0.37 | 0.0266 | bta-miR-222 | ENSBTAG00000001782_MRPS12 |
| -0.38 | 0.0224 | bta-miR-222 | ENSBTAG00000001803_FHL5 |
| -0.38 | 0.0224 | bta-miR-222 | ENSBTAG00000001804_GPR179 |
| -0.45 | 0.0061 | bta-miR-222 | ENSBTAG00000001806_IQCD |
| -0.41 | 0.0132 | bta-miR-222 | ENSBTAG00000001817_TMEM161A |

1. **bta-miR-30b-5p**





| **Correlation** | ***P* value** | **Correlated** | **With** |
| --- | --- | --- | --- |
| -0.33 | 0.0468 | bta-miR-30b-5p | ENSBTAG00000001778_NFKBIB |

1. **bta-miR-30c**


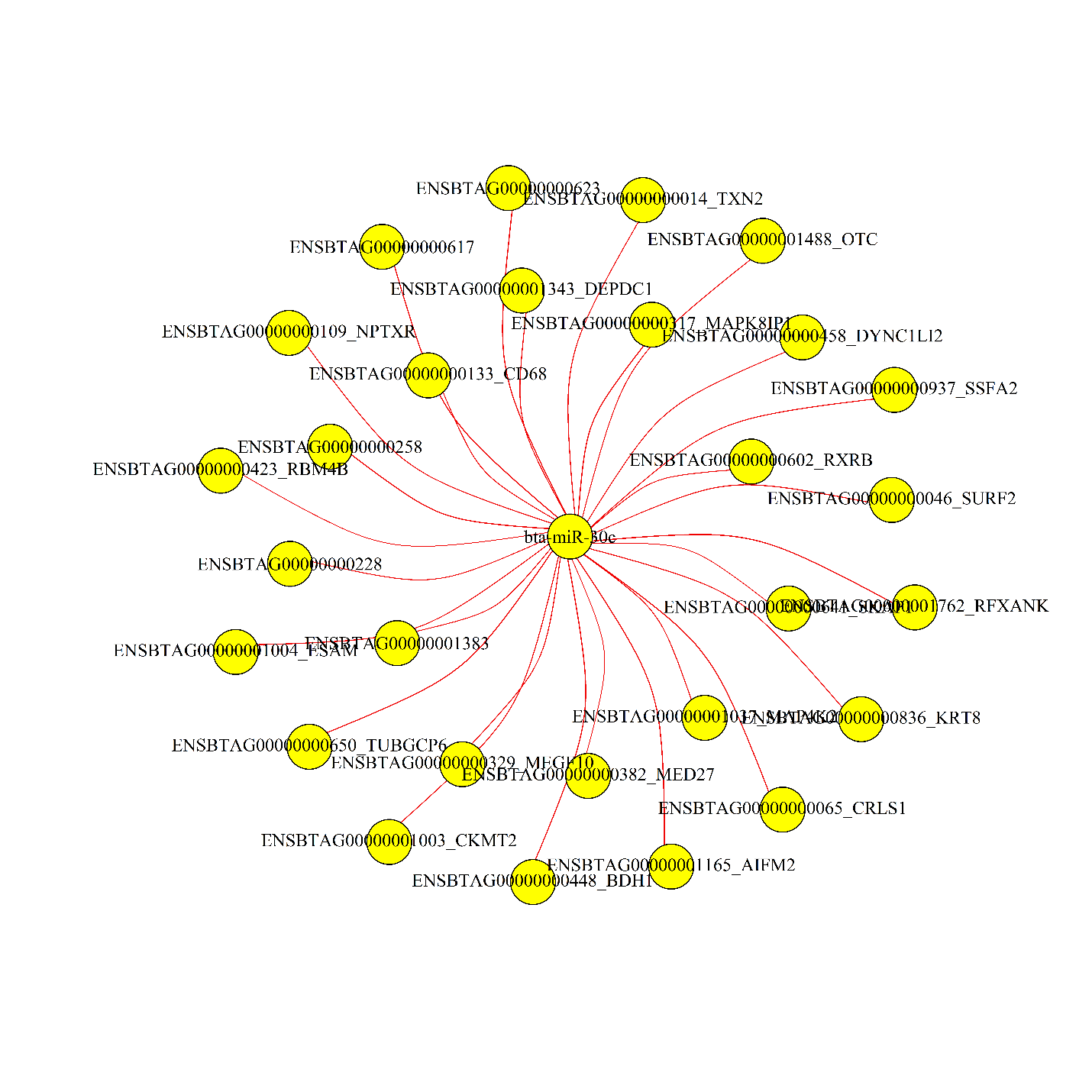


| **Correlation** | ***P* value** | **Correlated** | **With** |
| --- | --- | --- | --- |
| -0.35 | 0.0341 | bta-miR-30c | ENSBTAG00000000014_TXN2 |
| -0.35 | 0.0341 | bta-miR-30c | ENSBTAG00000000046_SURF2 |
| -0.33 | 0.0468 | bta-miR-30c | ENSBTAG00000000065_CRLS1 |
| -0.35 | 0.0341 | bta-miR-30c | ENSBTAG00000000109_NPTXR |
| -0.37 | 0.0245 | bta-miR-30c | ENSBTAG00000000133_CD68 |
| -0.37 | 0.0245 | bta-miR-30c | ENSBTAG00000000228 |
| -0.33 | 0.0468 | bta-miR-30c | ENSBTAG00000000258 |
| -0.33 | 0.0468 | bta-miR-30c | ENSBTAG00000000317_MAPK8IP1 |
| -0.35 | 0.0341 | bta-miR-30c | ENSBTAG00000000329_MEGF10 |
| -0.39 | 0.0173 | bta-miR-30c | ENSBTAG00000000382_MED27 |
| -0.39 | 0.0173 | bta-miR-30c | ENSBTAG00000000423_RBM4B |
| -0.33 | 0.0468 | bta-miR-30c | ENSBTAG00000000448_BDH1 |
| -0.35 | 0.0341 | bta-miR-30c | ENSBTAG00000000458_DYNC1LI2 |
| -0.36 | 0.029 | bta-miR-30c | ENSBTAG00000000602_RXRB |
| -0.33 | 0.0468 | bta-miR-30c | ENSBTAG00000000617 |
| -0.33 | 0.0468 | bta-miR-30c | ENSBTAG00000000623 |
| -0.39 | 0.0173 | bta-miR-30c | ENSBTAG00000000641_SKAP1 |
| -0.33 | 0.0468 | bta-miR-30c | ENSBTAG00000000650_TUBGCP6 |
| -0.34 | 0.04 | bta-miR-30c | ENSBTAG00000000836_KRT8 |
| -0.35 | 0.0341 | bta-miR-30c | ENSBTAG00000000937_SSFA2 |
| -0.35 | 0.0341 | bta-miR-30c | ENSBTAG00000001003_CKMT2 |
| -0.35 | 0.0341 | bta-miR-30c | ENSBTAG00000001004_ESAM |
| -0.35 | 0.0341 | bta-miR-30c | ENSBTAG00000001037_MAP4K2 |
| -0.33 | 0.0468 | bta-miR-30c | ENSBTAG00000001165_AIFM2 |
| -0.35 | 0.0341 | bta-miR-30c | ENSBTAG00000001343_DEPDC1 |
| -0.37 | 0.0245 | bta-miR-30c | ENSBTAG00000001383 |
| -0.37 | 0.0245 | bta-miR-30c | ENSBTAG00000001488_OTC |
| -0.33 | 0.0468 | bta-miR-30c | ENSBTAG00000001762_RFXANK |

1. **bta-miR-30d**





| **Correlation** | ***P* value** | **Correlated** | **With** |
| --- | --- | --- | --- |
| -0.35 | 0.0341 | bta-miR-30d | ENSBTAG00000000437_FFAR4 |

1. **bta-miR-320a**





| **Correlation** | ***P* value** | **Correlated** | **With** |
| --- | --- | --- | --- |
| -0.35 | 0.04761 | bta-miR-320a | ENSBTAG00000001078_SRL |
| -0.35 | 0.0354 | bta-miR-320a | ENSBTAG0000001576_TMEM140 |
| -0.34 | 0.0257 | bta-miR-320a | ENSBTAG00000001289_MRPL18 |

1. **bta-miR-328**





| **Correlation** | ***P* value** | **Correlated** | **With** |
| --- | --- | --- | --- |
| -0.38 | 0.0302 | bta-miR-328 | ENSBTAG00000000916_TCTE3 |

1. **bta-miR-378**


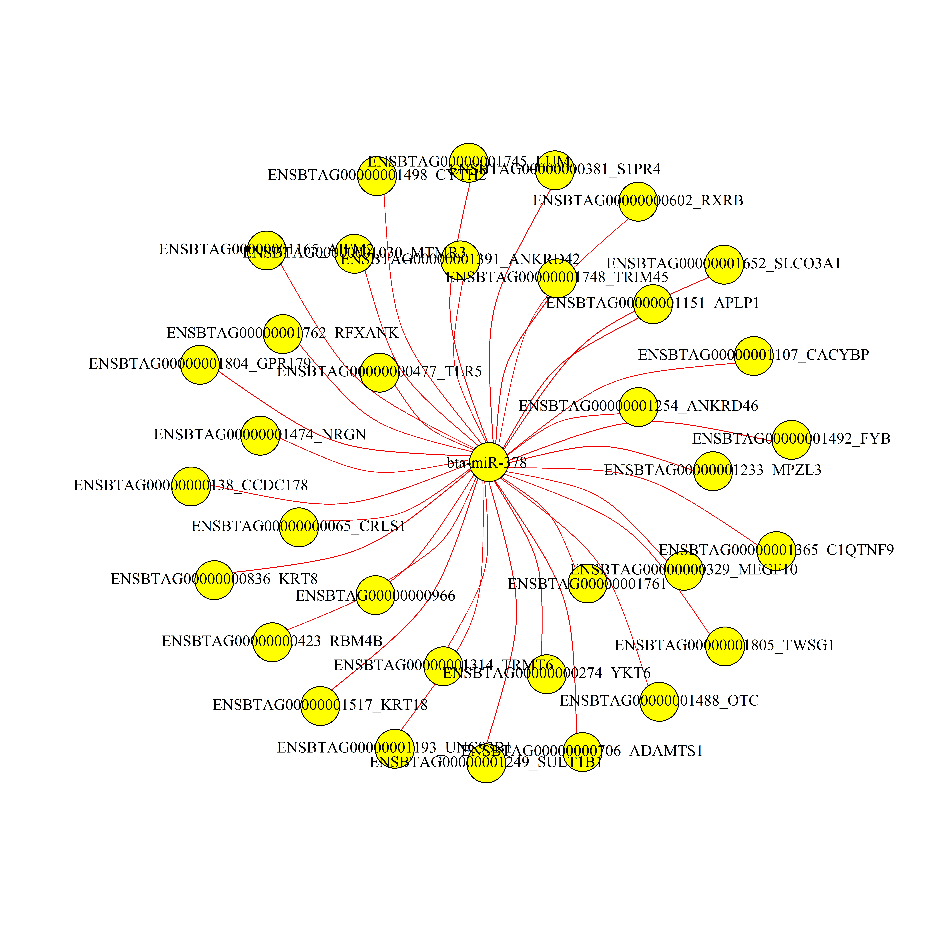


| **Correlation** | ***P* value** | **Correlated** | **With** |
| --- | --- | --- | --- |
| -0.36 | 0.029 | bta-miR-378 | ENSBTAG00000000065_CRLS1 |
| -0.34 | 0.04 | bta-miR-378 | ENSBTAG00000000138_CCDC178 |
| -0.33 | 0.0468 | bta-miR-378 | ENSBTAG00000000274_YKT6 |
| -0.38 | 0.0206 | bta-miR-378 | ENSBTAG00000000329_MEGF10 |
| -0.33 | 0.0468 | bta-miR-378 | ENSBTAG00000000381_S1PR4 |
| -0.36 | 0.029 | bta-miR-378 | ENSBTAG00000000423_RBM4B |
| -0.34 | 0.04 | bta-miR-378 | ENSBTAG00000000477_TLR5 |
| -0.39 | 0.0173 | bta-miR-378 | ENSBTAG00000000602_RXRB |
| -0.33 | 0.046 | bta-miR-378 | ENSBTAG00000000706_ADAMTS1 |
| -0.33 | 0.0468 | bta-miR-378 | ENSBTAG00000000836_KRT8 |
| -0.34 | 0.04 | bta-miR-378 | ENSBTAG00000000966 |
| -0.34 | 0.04 | bta-miR-378 | ENSBTAG00000001030_MTMR3 |
| -0.34 | 0.04 | bta-miR-378 | ENSBTAG00000001107_CACYBP |
| -0.33 | 0.0468 | bta-miR-378 | ENSBTAG00000001151_APLP1 |
| -0.34 | 0.04 | bta-miR-378 | ENSBTAG00000001165_AIFM2 |
| -0.38 | 0.0206 | bta-miR-378 | ENSBTAG00000001193_UNC93B1 |
| -0.33 | 0.0468 | bta-miR-378 | ENSBTAG00000001233_MPZL3 |
| -0.33 | 0.0468 | bta-miR-378 | ENSBTAG00000001249_SULT1B1 |
| -0.34 | 0.04 | bta-miR-378 | ENSBTAG00000001254_ANKRD46 |
| -0.34 | 0.04 | bta-miR-378 | ENSBTAG00000001314_TRMT6 |
| -0.34 | 0.04 | bta-miR-378 | ENSBTAG00000001365_C1QTNF9 |
| -0.37 | 0.0245 | bta-miR-378 | ENSBTAG00000001391_ANKRD42 |
| -0.36 | 0.029 | bta-miR-378 | ENSBTAG00000001474_NRGN |
| -0.36 | 0.029 | bta-miR-378 | ENSBTAG00000001488_OTC |
| -0.33 | 0.0468 | bta-miR-378 | ENSBTAG00000001492_FYB |
| -0.36 | 0.029 | bta-miR-378 | ENSBTAG00000001498_CYTH2 |
| -0.33 | 0.0468 | bta-miR-378 | ENSBTAG00000001517_KRT18 |
| -0.33 | 0.0468 | bta-miR-378 | ENSBTAG00000001652_SLCO3A1 |
| -0.33 | 0.0468 | bta-miR-378 | ENSBTAG00000001745_LUM |
| -0.33 | 0.0468 | bta-miR-378 | ENSBTAG00000001748_TRIM45 |
| -0.35 | 0.0341 | bta-miR-378 | ENSBTAG00000001761 |
| -0.36 | 0.029 | bta-miR-378 | ENSBTAG00000001762_RFXANK |
| -0.33 | 0.0468 | bta-miR-378 | ENSBTAG00000001804_GPR179 |
| -0.36 | 0.029 | bta-miR-378 | ENSBTAG00000001805_TWSG1 |

1. **bta-miR-423-5p**





| **Correlation** | ***P* value** | **Correlated** | **With** |
| --- | --- | --- | --- |
| -0.33 | 0.0468 | bta-miR-423-5p | ENSBTAG00000000097_EFCAB14 |
| -0.34 | 0.04 | bta-miR-423-5p | ENSBTAG00000000309_PTPRT |
| -0.34 | 0.04 | bta-miR-423-5p | ENSBTAG00000000310_MFAP5 |
| -0.37 | 0.0386 | bta-miR-423-5p | ENSBTAG00000000521_PLEKHA8 |
| -0.39 | 0.0173 | bta-miR-423-5p | ENSBTAG00000000805_IQCF1 |
| -0.34 | 0.04 | bta-miR-423-5p | ENSBTAG00000001301_LRRC32 |
| -0.37 | 0.0245 | bta-miR-423-5p | ENSBTAG00000001571_LRRD1 |
| -0.35 | 0.0341 | bta-miR-423-5p | ENSBTAG00000001580_CLGN |
| -0.36 | 0.029 | bta-miR-423-5p | ENSBTAG00000001635_CUTA |
| -0.34 | 0.04 | bta-miR-423-5p | ENSBTAG00000001668_WNT7A |

1. **bta-miR-488**





| **Correlation** | ***P* value** | **Correlated** | **With** |
| --- | --- | --- | --- |
| -0.38 | 0.0351 | bta-miR-488 | ENSBTAG00000000109_NPTXR |
| -0.38 | 0.0351 | bta-miR-488 | ENSBTAG00000000245_NHSL1 |
| -0.37 | 0.042 | bta-miR-488 | ENSBTAG00000000758_TMEM206 |
| -0.37 | 0.042 | bta-miR-488 | ENSBTAG00000000894_PGK1 |
| -0.37 | 0.042 | bta-miR-488 | ENSBTAG00000001324_SLCO2A1 |
| -0.37 | 0.042 | bta-miR-488 | ENSBTAG00000001634_PHF1 |
| -0.37 | 0.042 | bta-miR-488 | ENSBTAG00000001704 |

1. **bta-miR-544a**





| **Correlation** | ***P* value** | **Correlated** | **With** |
| --- | --- | --- | --- |
| -0.37 | 0.042 | bta-miR-544a | ENSBTAG00000000109_NPTXR |

1. **bta-miR-631**





| **Correlation** | ***P* value** | **Correlated** | **With** |
| --- | --- | --- | --- |
| -0.33 | 0.0468 | bta-miR-631 | ENSBTAG00000000258 |
| -0.33 | 0.0468 | bta-miR-631 | ENSBTAG00000000382_MED27 |
| -0.35 | 0.0341 | bta-miR-631 | ENSBTAG00000000458_DYNC1LI2 |
| -0.33 | 0.0468 | bta-miR-631 | ENSBTAG00000000526_TOPBP1 |

1. **bta-miR-761**





| **Correlation** | ***P* value** | **Correlated** | **With** |
| --- | --- | --- | --- |
| -0.37 | 0.042 | bta-miR-761 | ENSBTAG00000001704 |

1. **bta-miR-874**


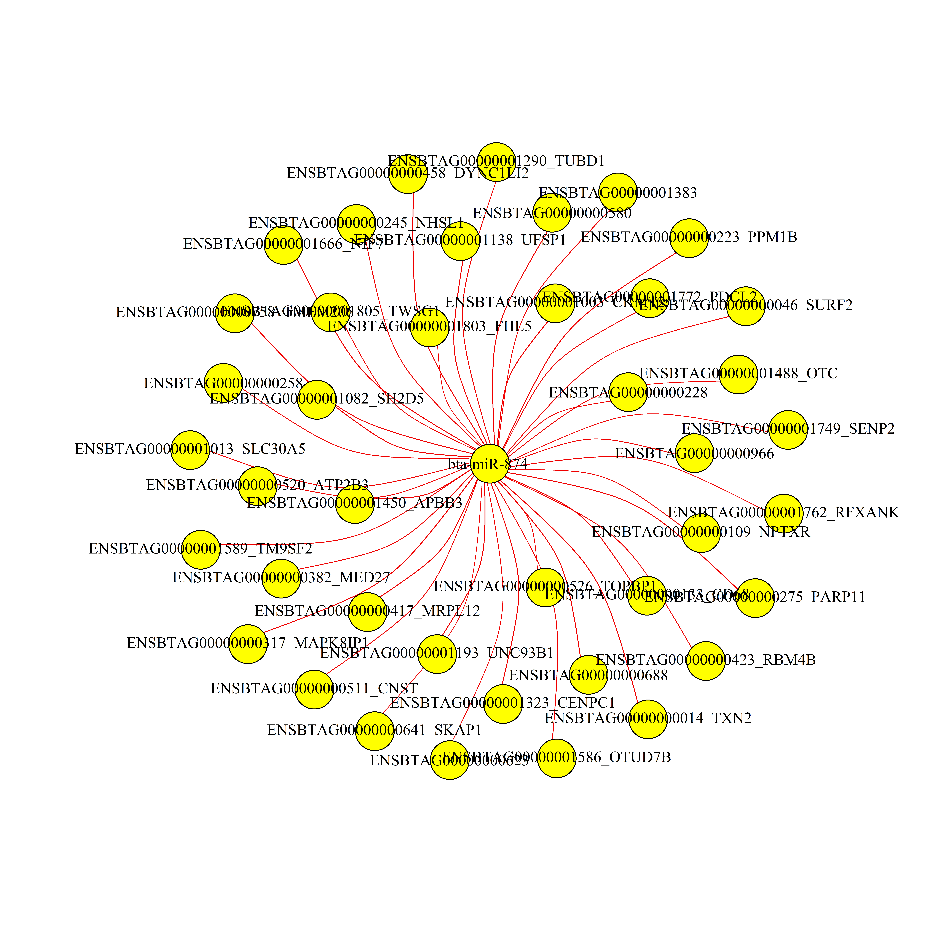


| **Correlation** | ***P* value** | **Correlated** | **With** |
| --- | --- | --- | --- |
| -0.33 | 0.0468 | bta-miR-874 | ENSBTAG00000000014_TXN2 |
| -0.35 | 0.0341 | bta-miR-874 | ENSBTAG00000000046_SURF2 |
| -0.37 | 0.0245 | bta-miR-874 | ENSBTAG00000000109_NPTXR |
| -0.33 | 0.0468 | bta-miR-874 | ENSBTAG00000000133_CD68 |
| -0.33 | 0.0468 | bta-miR-874 | ENSBTAG00000000223_PPM1B |
| -0.37 | 0.0245 | bta-miR-874 | ENSBTAG00000000228 |
| -0.35 | 0.0341 | bta-miR-874 | ENSBTAG00000000245_NHSL1 |
| -0.35 | 0.0341 | bta-miR-874 | ENSBTAG00000000258 |
| -0.33 | 0.0468 | bta-miR-874 | ENSBTAG00000000275_PARP11 |
| -0.33 | 0.0468 | bta-miR-874 | ENSBTAG00000000317_MAPK8IP1 |
| -0.35 | 0.0341 | bta-miR-874 | ENSBTAG00000000382_MED27 |
| -0.33 | 0.0468 | bta-miR-874 | ENSBTAG00000000417_MRPL12 |
| -0.35 | 0.0341 | bta-miR-874 | ENSBTAG00000000423_RBM4B |
| -0.33 | 0.0468 | bta-miR-874 | ENSBTAG00000000458_DYNC1LI2 |
| -0.35 | 0.0341 | bta-miR-874 | ENSBTAG00000000511_CNST |
| -0.35 | 0.0341 | bta-miR-874 | ENSBTAG00000000520_ATP2B3 |
| -0.39 | 0.0173 | bta-miR-874 | ENSBTAG00000000526_TOPBP1 |
| -0.33 | 0.0468 | bta-miR-874 | ENSBTAG00000000580 |
| -0.37 | 0.0245 | bta-miR-874 | ENSBTAG00000000623 |
| -0.39 | 0.0173 | bta-miR-874 | ENSBTAG00000000641_SKAP1 |
| -0.33 | 0.0468 | bta-miR-874 | ENSBTAG00000000688 |
| -0.33 | 0.0468 | bta-miR-874 | ENSBTAG00000000758_TMEM206 |
| -0.35 | 0.0341 | bta-miR-874 | ENSBTAG00000000966 |
| -0.33 | 0.0468 | bta-miR-874 | ENSBTAG00000001003_CKMT2 |
| -0.35 | 0.0341 | bta-miR-874 | ENSBTAG00000001013_SLC30A5 |
| -0.33 | 0.0468 | bta-miR-874 | ENSBTAG00000001082_SH2D5 |
| -0.33 | 0.0468 | bta-miR-874 | ENSBTAG00000001138_UFSP1 |
| -0.33 | 0.0468 | bta-miR-874 | ENSBTAG00000001193_UNC93B1 |
| -0.35 | 0.0341 | bta-miR-874 | ENSBTAG00000001290_TUBD1 |
| -0.33 | 0.0468 | bta-miR-874 | ENSBTAG00000001323_CENPC1 |
| -0.37 | 0.0245 | bta-miR-874 | ENSBTAG00000001383 |
| -0.33 | 0.0468 | bta-miR-874 | ENSBTAG00000001450_APBB3 |
| -0.37 | 0.0245 | bta-miR-874 | ENSBTAG00000001488_OTC |
| -0.35 | 0.0341 | bta-miR-874 | ENSBTAG00000001586_OTUD7B |
| -0.35 | 0.0341 | bta-miR-874 | ENSBTAG00000001589_TM9SF2 |
| -0.33 | 0.0468 | bta-miR-874 | ENSBTAG00000001666_NIP7 |
| -0.37 | 0.0245 | bta-miR-874 | ENSBTAG00000001749_SENP2 |
| -0.35 | 0.0341 | bta-miR-874 | ENSBTAG00000001762_RFXANK |
| -0.35 | 0.0341 | bta-miR-874 | ENSBTAG00000001772_PDCL2 |
| -0.39 | 0.0173 | bta-miR-874 | ENSBTAG00000001803_FHL5 |
| -0.37 | 0.0245 | bta-miR-874 | ENSBTAG00000001805_TWSG1 |

1. **bta-miR-93**


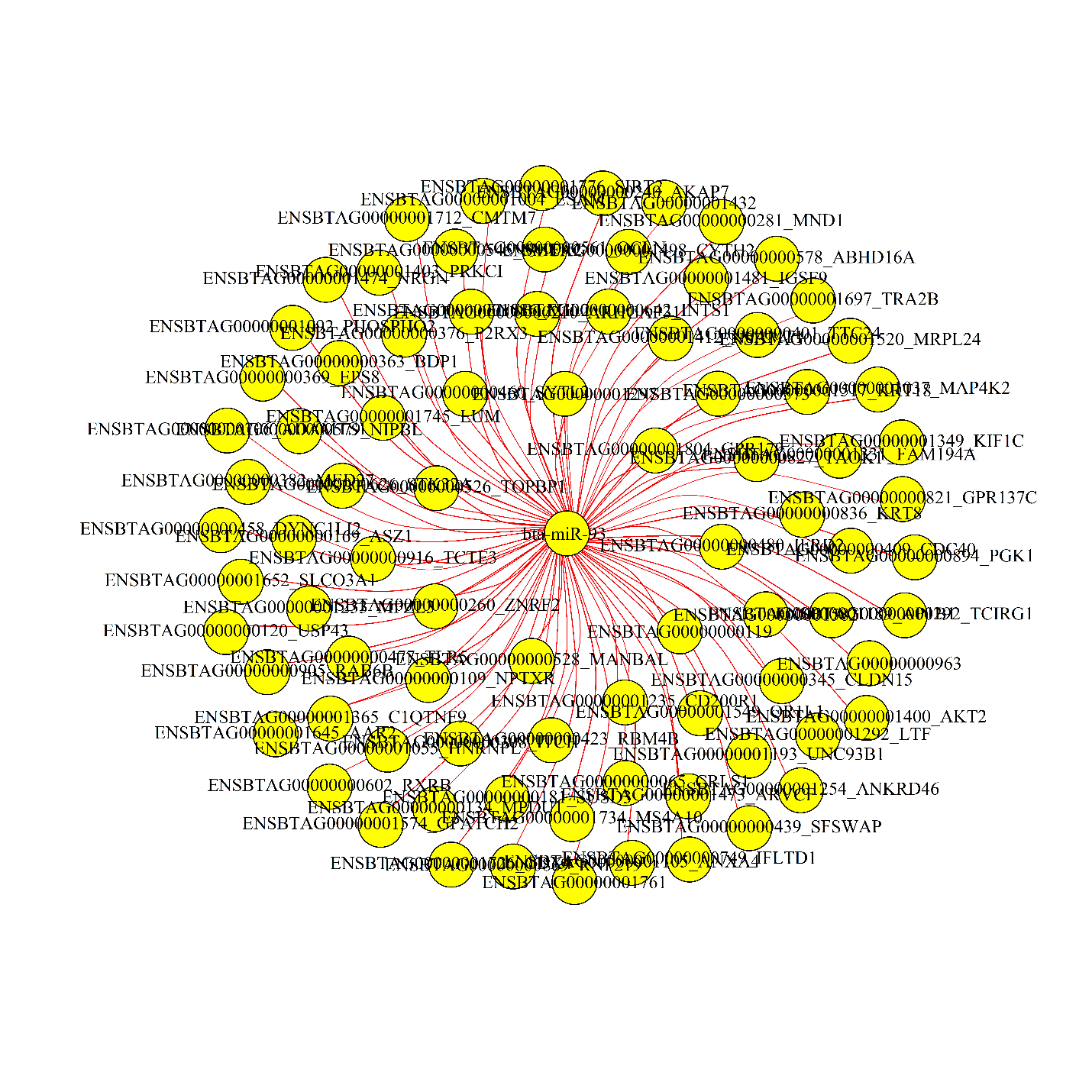


| **Correlation** | ***P* value** | **Correlated** | **With** |
| --- | --- | --- | --- |
| -0.44 | 0.0083 | bta-miR-93 | ENSBTAG00000000065_CRLS1 |
| -0.33 | 0.0468 | bta-miR-93 | ENSBTAG00000000109_NPTXR |
| -0.37 | 0.0245 | bta-miR-93 | ENSBTAG00000000119 |
| -0.34 | 0.04 | bta-miR-93 | ENSBTAG00000000120_USP43 |
| -0.4 | 0.0145 | bta-miR-93 | ENSBTAG00000000134_MPDU1 |
| -0.45 | 0.0068 | bta-miR-93 | ENSBTAG00000000169_ASZ1 |
| -0.36 | 0.029 | bta-miR-93 | ENSBTAG00000000181_SUSD3 |
| -0.36 | 0.029 | bta-miR-93 | ENSBTAG00000000210_ARHGAP31 |
| -0.33 | 0.0468 | bta-miR-93 | ENSBTAG00000000240_AKAP7 |
| -0.34 | 0.04 | bta-miR-93 | ENSBTAG00000000260_ZNRF2 |
| -0.38 | 0.0206 | bta-miR-93 | ENSBTAG00000000281_MND1 |
| -0.33 | 0.0468 | bta-miR-93 | ENSBTAG00000000292_TCIRG1 |
| -0.38 | 0.0206 | bta-miR-93 | ENSBTAG00000000308_ITCH |
| -0.33 | 0.0468 | bta-miR-93 | ENSBTAG00000000345_CLDN15 |
| -0.33 | 0.0468 | bta-miR-93 | ENSBTAG00000000363_BDP1 |
| -0.38 | 0.0206 | bta-miR-93 | ENSBTAG00000000369_EPS8 |
| -0.39 | 0.0173 | bta-miR-93 | ENSBTAG00000000376_P2RX3 |
| -0.35 | 0.0341 | bta-miR-93 | ENSBTAG00000000382_MED27 |
| -0.42 | 0.0121 | bta-miR-93 | ENSBTAG00000000401_TTC24 |
| -0.45 | 0.0068 | bta-miR-93 | ENSBTAG00000000409_CDC40 |
| -0.39 | 0.0173 | bta-miR-93 | ENSBTAG00000000423_RBM4B |
| -0.37 | 0.0245 | bta-miR-93 | ENSBTAG00000000439_SFSWAP |
| -0.33 | 0.0468 | bta-miR-93 | ENSBTAG00000000458_DYNC1LI2 |
| -0.33 | 0.0468 | bta-miR-93 | ENSBTAG00000000460_SYTL2 |
| -0.33 | 0.0468 | bta-miR-93 | ENSBTAG00000000477_TLR5 |
| -0.37 | 0.0245 | bta-miR-93 | ENSBTAG00000000480_IFRD2 |
| -0.33 | 0.0468 | bta-miR-93 | ENSBTAG00000000526_TOPBP1 |
| -0.36 | 0.029 | bta-miR-93 | ENSBTAG00000000528_MANBAL |
| -0.38 | 0.0206 | bta-miR-93 | ENSBTAG00000000545_SMEK2 |
| -0.38 | 0.0206 | bta-miR-93 | ENSBTAG00000000561_OCLN |
| -0.33 | 0.0468 | bta-miR-93 | ENSBTAG00000000573 |
| -0.36 | 0.029 | bta-miR-93 | ENSBTAG00000000578_ABHD16A |
| -0.34 | 0.0438 | bta-miR-93 | ENSBTAG00000000579_NIPBL |
| -0.4 | 0.0145 | bta-miR-93 | ENSBTAG00000000602_RXRB |
| -0.34 | 0.04 | bta-miR-93 | ENSBTAG00000000626_STK32A |
| -0.43 | 0.01 | bta-miR-93 | ENSBTAG00000000642_INTS1 |
| -0.4 | 0.0169 | bta-miR-93 | ENSBTAG00000000706_ADAMTS1 |
| -0.35 | 0.0341 | bta-miR-93 | ENSBTAG00000000749_IFLTD1 |
| -0.45 | 0.0068 | bta-miR-93 | ENSBTAG00000000821_GPR137C |
| -0.37 | 0.0245 | bta-miR-93 | ENSBTAG00000000827_TAOK1 |
| -0.45 | 0.0068 | bta-miR-93 | ENSBTAG00000000836_KRT8 |
| -0.35 | 0.0341 | bta-miR-93 | ENSBTAG00000000869_RNF219 |
| -0.33 | 0.0468 | bta-miR-93 | ENSBTAG00000000894_PGK1 |
| -0.37 | 0.0245 | bta-miR-93 | ENSBTAG00000000905_RAB6B |
| -0.33 | 0.0468 | bta-miR-93 | ENSBTAG00000000916_TCTE3 |
| -0.37 | 0.0245 | bta-miR-93 | ENSBTAG00000000963 |
| -0.39 | 0.0173 | bta-miR-93 | ENSBTAG00000001004_ESAM |
| -0.33 | 0.0468 | bta-miR-93 | ENSBTAG00000001037_MAP4K2 |
| -0.36 | 0.029 | bta-miR-93 | ENSBTAG00000001055_HNRNPL |
| -0.35 | 0.0341 | bta-miR-93 | ENSBTAG00000001086_FHL2 |
| -0.33 | 0.0468 | bta-miR-93 | ENSBTAG00000001092_PHOSPHO2 |
| -0.34 | 0.04 | bta-miR-93 | ENSBTAG00000001105_ANXA4 |
| -0.33 | 0.0468 | bta-miR-93 | ENSBTAG00000001189_AP1B1 |
| -0.33 | 0.0468 | bta-miR-93 | ENSBTAG00000001193_UNC93B1 |
| -0.38 | 0.0206 | bta-miR-93 | ENSBTAG00000001233_MPZL3 |
| -0.34 | 0.04 | bta-miR-93 | ENSBTAG00000001235_CD200R1 |
| -0.37 | 0.0245 | bta-miR-93 | ENSBTAG00000001254_ANKRD46 |
| -0.39 | 0.0173 | bta-miR-93 | ENSBTAG00000001292_LTF |
| -0.33 | 0.0468 | bta-miR-93 | ENSBTAG00000001297 |
| -0.37 | 0.0245 | bta-miR-93 | ENSBTAG00000001331_FAM194A |
| -0.39 | 0.0173 | bta-miR-93 | ENSBTAG00000001349_KIF1C |
| -0.44 | 0.0083 | bta-miR-93 | ENSBTAG00000001365_C1QTNF9 |
| -0.35 | 0.0341 | bta-miR-93 | ENSBTAG00000001400_AKT2 |
| -0.37 | 0.0245 | bta-miR-93 | ENSBTAG00000001403_PRKCI |
| -0.36 | 0.029 | bta-miR-93 | ENSBTAG00000001412_N6AMT1 |
| -0.44 | 0.0083 | bta-miR-93 | ENSBTAG00000001432 |
| -0.38 | 0.0206 | bta-miR-93 | ENSBTAG00000001473_ARVCF |
| -0.35 | 0.0341 | bta-miR-93 | ENSBTAG00000001474_NRGN |
| -0.38 | 0.0206 | bta-miR-93 | ENSBTAG00000001481_IGSF9 |
| -0.37 | 0.0245 | bta-miR-93 | ENSBTAG00000001498_CYTH2 |
| -0.4 | 0.0145 | bta-miR-93 | ENSBTAG00000001517_KRT18 |
| -0.33 | 0.0468 | bta-miR-93 | ENSBTAG00000001520_MRPL24 |
| -0.35 | 0.0341 | bta-miR-93 | ENSBTAG00000001549_OR1L1 |
| -0.34 | 0.04 | bta-miR-93 | ENSBTAG00000001574_GPATCH2 |
| -0.4 | 0.02 | bta-miR-93 | ENSBTAG00000001582 |
| -0.35 | 0.0341 | bta-miR-93 | ENSBTAG00000001645_AAR2 |
| -0.34 | 0.04 | bta-miR-93 | ENSBTAG00000001652_SLCO3A1 |
| -0.44 | 0.0083 | bta-miR-93 | ENSBTAG00000001697_TRA2B |
| -0.37 | 0.0245 | bta-miR-93 | ENSBTAG00000001712_CMTM7 |
| -0.37 | 0.0245 | bta-miR-93 | ENSBTAG00000001726_CDX4 |
| -0.33 | 0.0468 | bta-miR-93 | ENSBTAG00000001734_MS4A10 |
| -0.34 | 0.04 | bta-miR-93 | ENSBTAG00000001745_LUM |
| -0.38 | 0.0206 | bta-miR-93 | ENSBTAG00000001761 |
| -0.37 | 0.0245 | bta-miR-93 | ENSBTAG00000001776_SIRT2 |
| -0.34 | 0.04 | bta-miR-93 | ENSBTAG00000001804_GPR179 |

1. **RNT43 snoRNA**





| **Correlation** | ***P* value** | **Correlated** | **With** |
| --- | --- | --- | --- |
| -0.37 | 0.042 | RNT43 snoRNA | ENSBTAG00000001324_SLCO2A1 |
