# Supplementary material for: Steroidal Regulation of Oviductal microRNAs Is Associated with microRNA-Processing in Beef Cows
Source: Int J Mol Sci. 2021 Jan 19;22(2):953. doi: 10.3390/ijms22020953 (PMC7835783; doi:10.3390/ijms22020953)
Supplement: Supplementary file 1 [file ijms-22-00953-s001.zip › Supplementary Material 5.docx]

**Supplementary Material 5.** Alignment of the bovine microRNAs sequence with the human microRNA sequence and the primer sequence. The alignment was performed using the multiple sequence alignment tool of CLUSTAL 0 (1.2.3)

| **microRNA** | **Alignment result** |
| --- | --- |
| bta‐let‐7a‐5p | hsa‐let‐7a‐5p UGAGGUAGUAGGUUGUAUAGUU  bta‐let‐7a‐5p UGAGGUAGUAGGUUGUAUAGUU  primer TGAGGTAGTAGGTTGTATAGTT  **** ** *** * * ** |
| bta‐let‐7b | bta‐let‐7b UGAGGUAGUAGGUUGUGUGGUU  hsa‐let‐7b UGAGGUAGUAGGUUGUGUGGUU  primer TGAGGTAGTAGGTTGTGTGGTT  **** ** *** * * ** |
| bta‐let‐7e | bta‐let‐7e UGAGGUAGGAGGUUGUAUAGU  hsa‐let‐7e UGAGGUAGGAGGUUGUAUAGU  primer TGAGGTAGGAGGTTGTATAGT  **** ****** * * ** |
| bta‐mir‐10b | hsa‐mir‐10b UAGAACCGAAUUUGUGUGGUAUC  bta‐mir‐10b UACCCUGUAGAACCGAAUUUGUG  primer TACCCTGTAGAACCGAATTTGTG  **** * ********* * * |
| bta‐mir‐15a | bta‐mir‐15a UAGCAGCACAUAAUGGUUUGU  hsa‐mir‐15a UAGCAGCACAUAAUGGUUUGU  primer TAGCAGCACATAATGGTTTGT  ********* ** ** * |
| bta‐mir‐24‐1 | bta‐mir‐24‐1 UGGCUCAGUUCAGCAGGAACAG  bta‐mir‐24‐2 UGGCUCAGUUCAGCAGGAACAG  hsa‐mir‐24‐2 UGGCUCAGUUCAGCAGGAACAG  hsa‐mir‐24‐1 UGGCUCAGUUCAGCAGGAACAG  primer TGGCTCAGTTCAGCAGGAACAG  *** *** ************ |
| bta‐mir‐29a | hsa‐mir‐29a CUAGCACCAUCUGAAAUCGGUUA  bta‐mir‐29a CUAGCACCAUCUGAAAUCGGUUA  primer CTAGCACCATCTGAAATCGGTTA  * ******* * **** *** * |
| bta‐miR‐29b | bta‐miR‐29b UAGCACCAUUUGAAAUCAGUGUU  hsa‐mir‐29b‐1 UAGCACCAUUUGAAAUCAGUGUU  primer TAGCACCATTTGAAATCAGTGTT  ******* **** *** * |
| bta‐mir‐29c | hsa‐mir‐29c UAGCACCAUUUGAAAUCGGUUA  bta‐mir‐29c UAGCACCAUUUGAAAUCGGUUA  primer TAGCACCATTTGAAATCGGTTA  ******* **** *** * |
| bta‐mir‐30b | bta‐mir‐30b UGUAAACAUCCUACACUCAGCU  hsa‐mir‐30b UGUAAACAUCCUACACUCAGCU  PRIMER TGTAAACATCCTACACTCAGCT  * ***** ** **** **** |
| bta‐mir‐30d | hsa‐mir‐30d UGUAAACAUCCCCGACUGGAAGCU  bta‐mir‐30d UGUAAACAUCCCCGACUGGAAGCU  primer TGTAAACATCCCCGACTGGAAGCT  * ***** ******* ****** |
| bta‐mir‐34a | hsa‐mir‐34a UGGCAGUGUCUUAGCUGGUUGU  bta‐mir‐34a UGGCAGUGUCUUAGCUGGUUGU  primer TGGCAGTGTCTTAGCTGGTTGT  ***** * * *** ** * |
| bta‐mir‐92a | hsa‐mir‐92a‐1 UAUUGCACUUGUCCCGGCCUGU  hsa‐mir‐92a‐2 UAUUGCACUUGUCCCGGCCUGU  bta‐mir‐92a‐2 UAUUGCACUUGUCCCGGCCUGU  bta‐mir‐92a‐1 UAUUGCACUUGUCCCGGCCUGU  primer TATTGCACTTGTCCCGGCCTGT  * **** * ******* * |
| bta‐mir‐93 | hsa‐mir‐93 CAAAGUGCUGUUCGUGCAGGUA  bta‐mir‐93 CAAAGUGCUGUUCGUGCAGGUA  primer CAAAGTGCTGTTCGTGCAGGTA  ***** ** * ** ***** * |
| bta‐miR‐101 | hsa‐mir‐101‐1 UACAGUACUGUGAUAACUGAAGGAUGGCA  bta‐miR‐101 UACAGUACUGUGAUAACUGAA  primer TACAGTACTGTGATAACTGAA  **** ** * ** *** *** |
| bta‐miR‐103 | bta‐miR‐103 AGCAGCAUUGUACAGGGCUAUGA  hsa‐miR‐103a‐3p AGCAGCAUUGUACAGGGCUAUGA  primer AGCAGCATTGTACAGGGCTATGA  ******* * ******* * ** |
| bta‐miR‐199a‐5p | hsa‐miR‐199a‐5p CCCAGUGUUCAGACUACCUGUUC  bta‐miR‐199a‐5p CCCAGUGUUCAGACUACCUGUU  primer CCCAGTGTTCAGACTACCTGTT‐  ***** * ***** *** * |
| bta‐mir‐199b | hsa‐mir‐199b CCCAGUGUUUAGACUAUCUGUUC  bta‐mir‐199b CCCAGUGUUUAGACUAUCUGUUC  primer CCCAGTGTTTAGACTATCTGTTC  ***** * **** * * * * |
| bta‐mir‐222 | hsa‐mir‐222 AGCUACAUCUGGCUACUGGGU  bta‐mir‐222 AGCUACAUCUGGCUACUGGGU  primer AGCTACATCTGGCTACTGGGT  *** *** * *** ** *** |
| bta‐mir‐296 | hsa‐mir‐296 GAGGGCCCCCCCUCAAUCCU  bta‐mir‐296 GAGGGCCCCCC‐CCAAUCCU  primer GAGGGCCCCCC‐CCAATCCT  *********** *** ** |
| bta‐mir‐320a | hsa‐mir‐320a AAAAG CUGGGUUGAGAGGGC  bta‐mir‐320a‐2 AAAAG CUGGGUUGAGAGGGC  bta‐mir‐320a‐1 AAAAG CUGGGUUGAGAGGGC  PRIMER AAAAG CTGGGTTGAGAGGGC  ****** *** ********** |
| bta‐mir‐323 | hsa‐mir‐323b GCCCAAUACACGGUCGACCUCU  hsa‐mir‐323a GCACAUUACACGGUCGACCUCU  bta‐mir‐323 GCACAUUACACGGUCGACCUCU  primer GCACATTACACGGTCGACCTCT  **.** ****** ***** * |
| bta‐mir‐375 | bta‐mir‐375 UUUUGUUCGUUCGGCUCGCGUGA  hsa‐mir‐375 UUUUGUUCGUUCGGCUCGCGUGA  primer TTTTGTTCGTTCGGCTCGCGTGA  * ** **** **** ** |
| bta‐miR‐423‐5p | bta‐miR‐423‐5p UGAGGGGCAGAGAGCGAGACUUU  hsa‐miR‐423‐5p UGAGGGGCAGAGAGCGAGACUUU  primer TGAGGGGCAGAGAGCGAGACTTT  ******************* |
| bta‐mir‐494 | bta‐mir‐494 UGAAACAUACACGGGAAACCUCUUUUUUAGUAUCAA  hsa‐mir‐494 UGAAACAUACACGGGAAACCUCUUUUUUAGUAUC‐  primer TGAAACATACACGGGAAACCTC  ****** ************ * |
| bta‐mir‐664b | hsa‐mir‐664b UAUUCAUUUGCCUCCCAGCCUAC  bta‐mir‐664b UAUUCAUUUAUCUCCCAGCCUAC  Primer TATTCATTTATCTCCCAGCCTAC  * ** . * ******* ** |
| bta‐mir‐665 | hsa‐mir‐665 ACCAGGAGGCUGAGGCCCC  bta‐mir‐665 ACCAGUAGGCCGAGGCCCC  primer ACCAGTAGGCCGAGGCCCC  ***** **** ******** |
